# Mechanisms underlying modulation of human GlyRα3 by Zn^2+^ and pH

**DOI:** 10.1126/sciadv.adr5920

**Published:** 2024-12-18

**Authors:** Kayla Kindig, Eric Gibbs, David Seiferth, Philip C. Biggin, Sudha Chakrapani

**Affiliations:** ^1^ Department of Physiology and Biophysics, School of Medicine, Case Western Reserve University, Cleveland, OH 44106-4970, USA.; ^2^ Cleveland Center for Membrane and Structural Biology, School of Medicine, Case Western Reserve University, Cleveland, OH 44106-4970, USA.; ^3^ Department of Pharmacology, School of Medicine, Case Western Reserve University, Cleveland, OH 44106-4970, USA.; ^4^ Structural Bioinformatics and Computational Biochemistry, Department of Biochemistry, University of Oxford, Oxford OX1 3QU, UK.

## Abstract

Glycine receptors (GlyRs) regulate motor control and pain processing in the central nervous system through inhibitory synaptic signaling. The subtype GlyRα3 expressed in nociceptive sensory neurons of the spinal dorsal horn is a key regulator of physiological pain perception. Disruption of spinal glycinergic inhibition is associated with chronic inflammatory pain states, making GlyRα3 an attractive target for pain treatment. GlyRα3 activity is modulated by numerous endogenous and exogenous ligands that consequently affect pain sensitization. To understand the mechanism of two such endogenous modulators, Zn^2+^ and protons, we have used cryo–electron microscopy to determine structures of full-length human GlyRα3 in various functional states. Whereas acidic pH reduces peak glycine response, Zn^2+^ displays biphasic modulation in a concentration-dependent manner. Our findings reveal the effector sites and also capture intermediate conformations in the gating cycle. Combined with molecular dynamics simulations and electrophysiology, this work provides important insights into GlyRα3 activation and regulation.

## Teaser

Cryo-EM, electrophysiology, and MD simulations reveal the basis for pH sensitivity and biphasic Zn^2+^ modulation of GlyRα3.

## INTRODUCTION

Glycine is the main inhibitory neurotransmitter of the spinal cord and brainstem (*1*), in contrast to the dominance of GABAergic transmission in most of the brain. Glycine receptors (GlyRs) are pentameric ligand-gated ion channels (pLGICs) and are permeable to chloride ions, inhibiting the cells on which they are expressed via hyperpolarization or shunting inhibition. GlyRs control the excitability of motor and sensory circuits and thereby regulate neuronal development and neurophysiological processes such as respiration, vision (*2*), hearing (*3*), and pain perception (*4*, *5*). Dysregulation of GlyRs influences a wide range of conditions including chronic pain, autism (*6*), temporal lobe epilepsy (*7*), and hyperekplexia (*8*–*10*). The human GlyR α isoforms (α1 to 3) are ~90% similar in sequence but have differences in expression pattern that contribute to their distinct roles (*11*, *12*). GlyRα3 is localized primarily to lamina II of the spinal cord dorsal horn where nociceptive afferents terminate. One mechanism of prostaglandin-mediated inflammatory pain is PKA (cyclic adenosine monophosphate–dependent protein kinase)–dependent phosphorylation of Ser^346^ in the intracellular domain (ICD) of GlyRα3, which leads to a reduction in whole-cell glycinergic currents and disinhibition of pain-sensing neurons (*4*). Positive allosteric modulators (PAMs) of GlyRs, such as cannabinoids, can be used to induce analgesia in mouse models of inflammatory pain (*5**,* *13**,* *14*). Given the tissue-specific expression of various GlyR subtypes and their unique roles played in motor versus sensory function, there is a need for subtype-specific modulators. Understanding the GlyRα3 structure and how it may be modulated is highly relevant for the design of better analgesic drugs with fewer off-target effects.

Zinc (Zn^2+^) is an essential trace mineral that plays a crucial role in many biological processes. In the central nervous system (CNS), glutamatergic synaptic vesicles contain millimolar concentrations of Zn^2+^ and, upon release, can potentially raise the local extracellular concentration from nanomolar levels up to 100 to 300 μM (*15*–*17*). Whereas Zn^2+^ is necessary for proper CNS function, contributing to the development of the postsynaptic density (*18*) and neuroplasticity via long-term potentiation (*19*) and depression (*20*), elevated levels of intracellular Zn^2+^ are associated with ischemic injury and lead to neuronal cell death through numerous signaling pathways (*21**,* *22*). Zn^2+^ is also thought to be involved in nociception as neuropathic pain correlates with a reduction in Zn^2+^ in the synaptic vesicles of dorsal horn spinal neurons (*23*) and injection of ZnCl_2_ decreases thermal hyperalgesia in rats that have been subjected to sciatic nerve injury (*24*). Zn^2+^ acts as a neuromodulator affecting the activity of several synaptic receptors including NMDARs (*N*-methyl-d-aspartate receptors), GABA_A_Rs (γ-aminobutyric acid type A receptors), and GlyRs (*17**,* *25*–*28*). The effect of Zn^2+^ on GABA_A_Rs is inhibitory, but its effect on GlyRs is more complex. GlyRs experience a biphasic modulation where nanomolar and low micromolar concentrations of Zn^2+^ are potentiating, whereas higher micromolar and millimolar concentrations are inhibitory (*29*–*31*), with individual sensitivities varying in a subtype-specific manner (*32**,* *33*). This indicates the existence of both a high-affinity and low-affinity Zn^2+^ binding site for GlyRs, each with its own modulatory mechanism. Past studies have highlighted the importance of Zn^2+^ signaling in glycinergic neurotransmission (*34**,* *35*). Notably, GlyRα1 mutations that abolish Zn^2+^ potentiation are associated with a startle phenotype (*28**,* *36*).

Extracellular pH in the nervous system fluctuates with synaptic activity as protons are released from synaptic vesicles (*37**,* *38*). Under pathophysiological conditions such as ischemia (*39**,* *40*), epileptic seizure (*41**,* *42*), and spinal inflammation (*43**,* *44*), the extracellular environment can become even more acidic (by ~1 pH unit). Extracellular acidosis alters neurotransmission by modulating many receptors and channels, including voltage-gated ion channels, glutamate receptors, GABA_A_Rs, and GlyRs (*45*–*49*). Particularly, in the brainstem and spinal cord, acidification of extracellular pH is associated with impairment of glycinergic responses. Electrophysiological recordings from GlyRα1 show that, at pH 6.4, the glycine dose-response curve is right shifted, with a decrease in open channel probability, reduced activation, and slowed decay kinetics (*50*). In addition to reducing glycine sensitivity, acidic pH also reduces the maximal responses of partial agonists. Histidine typically has an acidic p*K*_a_ (where *K*_a_ is the acid dissociation constant) within the physiological range (*51**,* *52*), and thus extensive work has been done to examine the role of each extracellular histidine in the inhibition of GlyRs by protons (*53*). There are five extracellular histidines in the human GlyRα1 sequence, four of which are conserved in human GlyRα3 and GlyRα2 subunits. Mutation of His^109^ and His^215^ to alanine is sufficient to eliminate H^+^ inhibition in GlyRα1 at pH 6.4 (*54*).

Whereas there is ample functional evidence for Zn^2+^ and H^+^ modulation of GlyRs, a clear mechanistic picture is currently lacking. The first high-resolution glimpses of human GlyRα3 were revealed through x-ray crystallography of the channel in complex with strychnine and in the presence of glycine and PAMs (*55*–*57*). In the past few years, there has been tremendous progress in the GlyR field, propelled by insights from cryo–electron microscopy (cryo-EM) structures of various GlyR subtypes (GlyRα1, GlyRα2β, and GlyRα1β) in multiple conformational states (*58*–*64*) combined with data from protein dynamic measurements (*65*) and molecular dynamics (MD) simulations (*65*–*69*). However, advancements in the structural characterization of GlyRα3 have been comparatively slow. As a result, many fundamental questions regarding the mechanisms underlying GlyRα3 activation and modulation remain unanswered. In this study, we probe the molecular basis for GlyR regulation by these two critical synaptic factors (Zn^2+^ and H^+^) using a combination of structural, functional, and computational methods.

Here, we present cryo-EM structures of full-length human GlyRα3 solved in a peptidisc system and captured in both resting and activated states. We first look at the effect of different concentrations of glycine (submaximal and saturating levels) on the distribution of the functional states, and then we examine the effect of Zn^2+^ and acidic pH on this distribution. We find that there is a shift toward activated states under potentiating conditions (low Zn^2+^). Under inhibitory conditions (high Zn^2+^ and low pH), resting and intermediate states are more populated. At high concentrations, Zn^2+^ coordination is clearly visible in both binding sites, involving previously identified residues. In addition, both the low-affinity and high-affinity zinc-binding sites engage residues in the interaction network that have not been described in prior functional studies. We find that acidic pH alters the closed conformation of the receptor and enables us to solve a previously unidentified state. Our structures are corroborated by electrophysiology and MD simulations, enabling a comprehensive view of GlyRα3 function and modulation.

## RESULTS

### Cryo-EM state distribution captures glycine sensitivity of GlyRα3

**Fig. 1.**
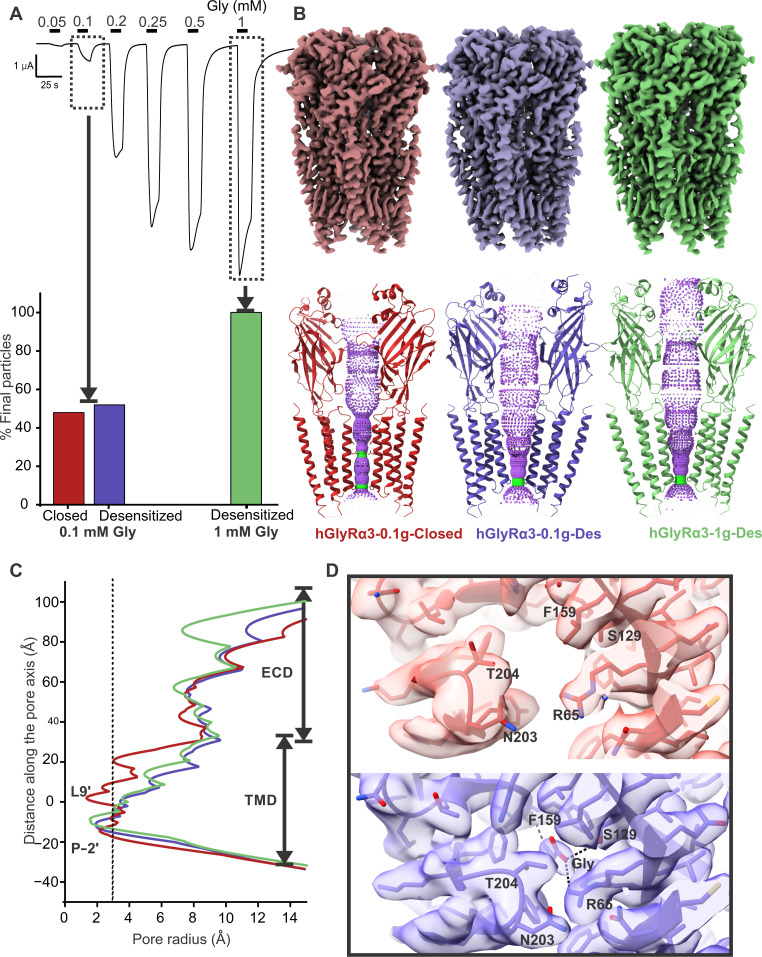
Evaluation of GlyRα3 agonism by glycine. (**A**) (Top) TEVC current trace showing response of GlyRα3 to various concentrations of glycine. Corresponding cryo-EM datasets were collected at the glycine concentrations indicated by boxes and arrows. (Bottom) Particle state distributions for cryo-EM datasets solved at low and high concentrations of glycine. (**B**) Side view of closed (left, red) and desensitized (middle, blue) state maps from the 0.1 mM glycine dataset and desensitized map from the 1 mM glycine dataset (right, green). (**C**) Plot of the pore radius for all three conformational states. Radius of a hydrated chloride ion is indicated by a dotted line. (**D**) Close-up of the glycine binding pocket in the closed state (top, red) and desensitized state (bottom, blue) for the 0.1 mM glycine condition. The ligand pocket does not have a density for glycine in the closed state. In the desensitized state, there is unambiguous density for glycine, and the molecule makes contacts with several key residues in the pocket.

Despite a high sequence similarity between GlyRα1 and GlyRα3 subtypes and absolute conservation of the binding site, GlyRα3 is less sensitive to glycine (*70*–*72*). In addition, glycine analogs such as l-alanine, β-alanine, l-serine, and taurine are significantly less effective on GlyRα3 than α1 or α2 subtypes. Differences in activation between receptor subtypes indicate distinct mechanisms that may underlie subtype-specific allosteric modulation. To understand these mechanistic differences, we initiated structural and functional studies of full-length human GlyRα3. The gene encoding GlyRα3 (*GLRA3*) was cloned into a pCS2+ plasmid for functional measurement by two-electrode voltage clamp (TEVC) in *Xenopus laevis* oocytes (data S1). Robust glycine responses were observed with a median effective concentration (EC_50_) of 213 ± 13 μM, compared to 111 ± 15 μM for GlyRα1 (fig. S1). We used a codon-optimized *GLRA3* sequence, with a thrombin cleavage site and an 8x C-terminal histidine tag, cloned into a pFastBac1 plasmid (data S2) to generate a baculovirus for expression in *Spodoptera frugiperda* (Sf9) cells. For cryo-EM studies, a detergent-solubilized protein from Sf9 cells was exchanged into peptidisc on an affinity column prior to elution (fig. S1) (*73**,* *74*). To capture the functional states, we first imaged GlyRα3 incubated with glycine at a concentration below the EC_50_. At a 100 μM glycine concentration, the final set of particles sorted to two distinct conformational states of roughly equal proportion (Fig. 1, A and B, and fig. S2). Both classes were of comparable resolution (2.5 to 2.6 Å) with high map quality through the extracellular domain (ECD) and the transmembrane domain (TMD) (fig. S3). As with other GlyR structures, the GlyRα3 ICD between Lys^312^ and Arg^385^ is largely unstructured and omitted from the model. Model building and structural comparison of the two states allowed unambiguous assignment of these to a resting/closed (hGlyRα3-0.1g-Closed, 2.5 Å) and desensitized conformation (hGlyRα3-0.1g-Des, 2.6 Å). The hGlyRα3-0.1g-Closed state features an empty neurotransmitter-binding pocket at the subunit interface and an open configuration of the capping loop C. Along the pore-lining M2 helices, narrow constrictions are observed at the Leu^261^ (Leu9′) and Pro^250^ (Pro-2′) positions, which form the activation and desensitization gates of the channel, respectively (Fig. 1, B and C). In contrast, the hGlyRα3-0.1g-Des state has a clear density for glycine in the binding pocket, and loop C is positioned inward in a closed configuration (Fig. 1D). The glycine molecule is in a network of hydrogen bond interactions—the carboxylate end interacts with Arg^65^ and Ser^129^ on the complementary subunit and the amino end interacts with the main chain of Phe^159^ on the principal subunit.

In the pore of the desensitized state, the M2 helices are expanded out at the extracellular end and constricted at the Pro-2′, leading to a cone-shaped permeation pathway. The radius of the pore at the Pro-2′ is ~1.9 Å, which is smaller than the radius of a solvated chloride ion (2.26 Å) and closer to its Pauling radius (1.81 Å) (*75*). A constriction of this magnitude is nonconducting, as reported in our previous MD simulations with GlyRα1 (*59**,* *76*). Both the closed and desensitized conformations are broadly similar to the corresponding states observed in the hGlyRα3 crystal structures (*55*–*57*). A comparison of the two structures shows the typical changes associated with the transition from the resting to desensitized states in pLGICs: a counterclockwise rotation of the ECD and an iris-like expansion of the TMD. As expected from the dose-response profiles of GlyRs at 100 μM glycine, whereas GlyRα1 particles sorted to only a desensitized conformation (*59*), GlyRα3 particles were found in an unliganded, closed state as well. However, with respect to glycine position or its interaction with the binding pocket, there are no noticeable differences. It is likely that the prevalence of the closed state may be determined by the effectiveness of the coupling between the ECD and TMD.

Increasing glycine to a saturating concentration (1 mM) for imaging conditions led to particles populating only the desensitized conformation (hGlyRα3-1g-Des, 2.8 Å) (Fig. 1, A and B, and figs. S4 and S5). Although the inability to resolve a state does not indicate its absence in a dataset, this difference in state distribution demonstrates the ability of cryo-EM to recapitulate the behavior of the receptor in solution, which has also been demonstrated with modulation of GlyRα1 by Δ^9^-tetrahydrocannabinol (*76*) and partial agonists (*60*). There is no substantial difference in conformation between the desensitized states determined at 100 μM and 1 mM glycine. When processed without imposing C5 symmetry, we did see some indication of states with partial glycine occupancy via the sorting of multiple desensitized states where the only difference was the strength of the glycine densities (fig. S4). Symmetry expansion allowed for the classification of subunits with and without a distinct glycine density, although both subunits were still in the desensitized conformation.

A comparison of the hGlyRα3-0.1g-Closed and hGlyRα3-0.1g-Des states with the GlyRα3 crystal structures in strychnine and glycine-bound conformations, respectively, shows similar structural changes associated with activation. However, in the closed states, the packing of the TM helices differs, with the M2 helices placed closer to the pore axis in the hGlyRα3-0.1g-Closed structure, creating a tighter constriction around the Pro-2′ position (fig. S6). These differences could arise from the peptidisc versus detergent environment, combined with the effects of ICD truncation and crystallization conditions.

### Zn^2+^ exerts a biphasic effect on GlyRα3

**Fig. 2.**
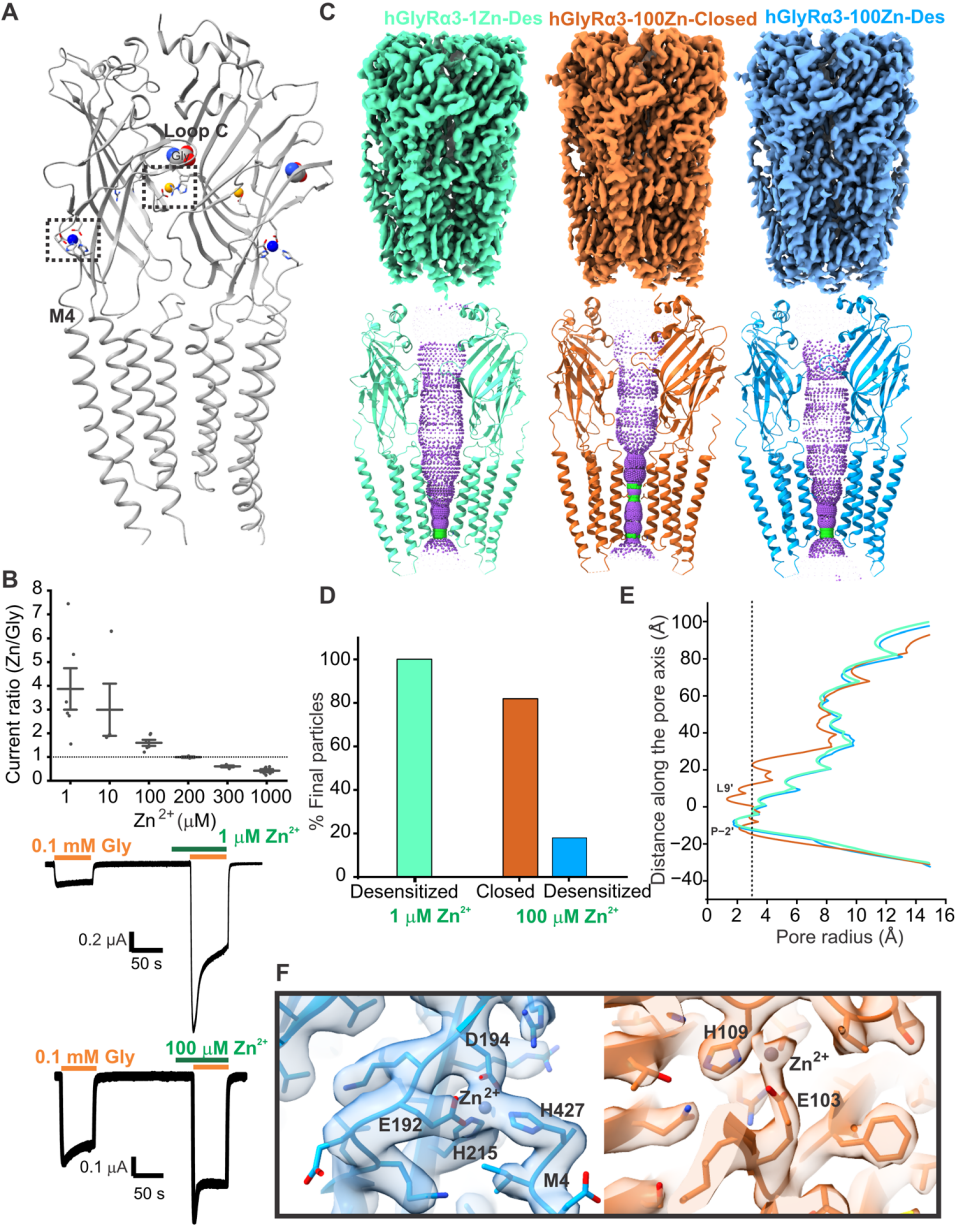
Zn^2+^ is a biphasic modulator of GlyRα3. (**A**) Model of two adjacent subunits of GlyRα3 to illustrate the position of the zinc interaction sites. The zinc ion is enlarged and shown in blue for the potentiating site and orange for the inhibitory site. Glycine is depicted in sphere representation. Boxes enclose the regions that are enlarged in (F). (**B**) Ratio of zinc-modulated glycine currents to glycine-only currents at different concentrations of Zn^2+^; dotted line indicates no modulation. Mean change is 3.9 ± 0.9 (*n* = 6) for 1 μM, 3 ± 1.1 (*n* = 4) for 10 μM, 1.6 ± 0.1 (*n* = 6) for 100 μM, 1 ± 0.03 (*n* = 3) for 200 μM, 0.6 ± 0.04 (*n* = 4) for 300 μM, and 0.4 ± 0.06 (*n* = 6) for 1 mM Zn^2+^. Representative TEVC traces of glycine-activated currents and coapplication (plus 30-s preapplication) of 1 μM (top trace) and 100 μM (bottom trace) zinc chloride. (**C**) Maps and hole profiles of the 1 μM Zn^2+^ desensitized state, the 100 μM Zn^2+^ closed state, and the 100 μM Zn^2+^ desensitized state. (**D**) Particle distribution of states for cryo-EM datasets collected with 0.1 mM glycine and either 1 or 100 μM Zn^2+^. (**E**) Plot of the pore radius for all three zinc-bound states. Radius of a hydrated chloride ion is indicated by a dotted line. (**F**) Close-up of the Zn^2+^ potentiating site in the 100 μM Zn^2+^ desensitized model/map (left, blue) and the inhibitory site in the 100 μM Zn^2+^ closed model/map (right, orange).

Prior functional studies indicate that the potentiating binding site for Zn^2+^ is composed of residues from the β9 and β10 strands, whereas the inhibitory binding site is positioned behind the glycine binding pocket (Fig. 2A), and we sought to visualize Zn^2+^ in these sites by solving the structure at a low and high Zn^2+^ concentration. We found that the glycine response in GlyRα3 is potentiated by Zn^2+^ at concentrations below 200 μM and inhibited at concentrations above 200 μM for 30-s preapplication in *X. laevis* oocytes (Fig. 2B). The effect of Zn^2+^ is diminished at a saturating glycine concentration (1 mM). We obtained cryo-EM structures of GlyRα3 preincubated with 1 μM ZnCl_2_ and 100 μM glycine, under conditions similar to those of 100 μM glycine alone. Analysis of the particles from the 1 μM Zn^2+^ dataset via three-dimensional (3D) classification allowed us to resolve a single conformation corresponding to that of the desensitized state (hGlyRα3-1Zn-Des, 2.9 Å) (Fig. 2, C to E, and figs. S7 and S8). Some particles were identified as corresponding to the closed state, but the number was insufficient to achieve a high-resolution structure (fig. S7). A shift in the particle distribution from the closed to desensitized state is consistent with the potentiating effect of 1 μM Zn^2+^. Whereas the overall conformation of hGlyRα3-1Zn-Des is similar to the desensitized state in the presence of 100 μM glycine alone (hGlyRα3-0.1g-Des), there is additional density corresponding to Zn^2+^ coordination noticeable in the previously predicted high-affinity potentiating site (fig. S9, B and C). A Zn^2+^ ion is coordinated at the ECD-TMD interface by negatively charged residues Glu^192^ and Asp^194^ (on the β9 strand) and His^215^ (on the β10 strand). This interaction was also observed in the GlyRα3 crystal structure (*57*). In addition to these residues, the Zn^2+^ ion appears to interact with His^427^ in the C-terminal end of M4 (post-M4 region). The continuity of the map beyond His^427^ is not well resolved, suggesting flexibility in this region. Notably, the map quality of the Zn^2+^ potentiating site is poorer for the 1 μM Zn^2+^ condition compared to that at 100 μM Zn^2+^ (fig. S9), which we attribute to partial occupancy of this site at the lower concentration. For clarity, figures show the binding sites as seen in the 100 μM Zn^2+^ structures. Earlier studies using mutagenesis and electrophysiological analysis in human embryonic kidney (HEK) cells determined that residues necessary for Zn^2+^ potentiation in GlyRα1 include Glu^192^, Asp^194^, and His^215^. A D194A or E192A mutation alone is sufficient to completely eliminate enhancement from Zn^2+^ (*32*). In addition, the D80A mutation that creates a hyperplexia-like motor phenotype in mice drastically reduces the potentiation of GlyRα1 by Zn^2+^ (*28**,* *77*). Because the D80A mutation does not eliminate Zn^2+^ potentiation of currents induced by the partial agonist taurine (*30*), the effect of the mutation is thought to be of an allosteric nature. Consistent with this idea, we see no additional Zn^2+^ density in the vicinity of Asp^80^. Similarly, whereas the hyperekplexia mutation W170S (*36*) and a T151A mutation (*32*) also attenuate Zn^2+^ potentiation of GlyRα1, we see no evidence for a direct interaction between these residues and Zn^2+^.

**Fig. 3.**
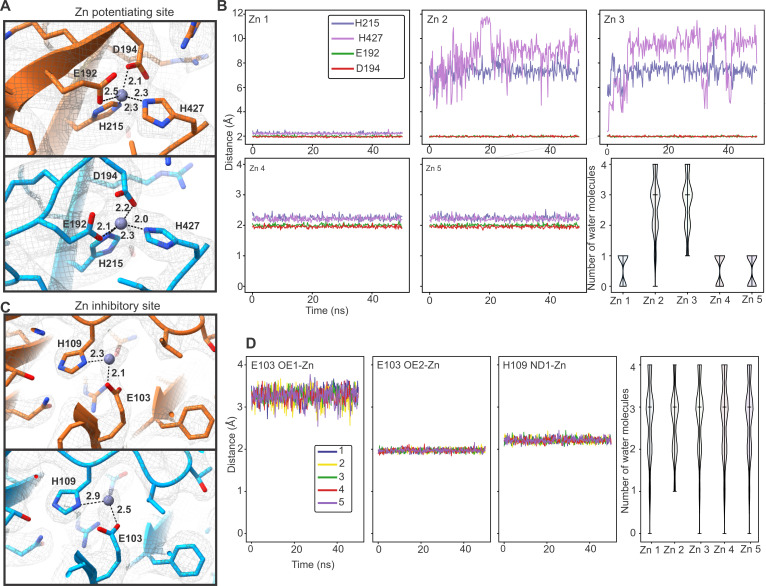
Examination of the low-affinity and high-affinity Zn^2+^ binding sites by MD simulations. (**A**) Zn^2+^ is present in the potentiating site in both the apo (top, orange) and desensitized (bottom, blue) state at a concentration of 100 μM Zn^2+^. Important residues for Zn^2+^ coordination are labeled, and the minimum distance to the zinc ion is indicated. (**B**) Zn^2+^ remains stably bound in the potentiation site during MD simulations. We evaluate the distance between Zn^2+^ and Glu^192^, Asp^194^, His^215^, and His^427^. Both histidines are neutral with the proton sitting on the δ nitrogen. The plot shows the distance between Zn^2+^ and the ϵ nitrogen on the histidines or the closest oxygen in the respective Glu or Asp residues. The number of water molecules within 2.15 Å of Zn^2+^ is plotted for each of the subunits. (**C**) Zn^2+^ is present in the inhibitory site in both the apo and desensitized state in the presence of 100 μM Zn^2+^. (**D**) Zn^2+^ remains stably bound in the inhibitory site during MD simulations. His^109^ is neutral with the proton sitting on the ϵ nitrogen. The distance between Zn^2+^ and the δ nitrogen of His^109^ or the oxygen (OE1 and OE2) of Glu^103^ is shown. We report the minimal, maximal, and mean number of water molecules within 2.15 Å of Zn^2+^.

Structural analysis of GlyRα3 in the presence of 100 μM glycine and 100 μM Zn^2+^ resulted in a closed (hGlyRα3-100Zn-Closed, 2.2 Å) and desensitized state (hGlyRα3-100Zn-Des, 2.5 Å) but with five times as many particles in the closed conformation (Fig. 2, C and D, and figs. S10 and S11). This concentration of Zn^2+^ is below our experimentally determined value for Zn^2+^ inhibition in oocytes, but the shift in states suggests inhibition of the purified receptor in solution. This discrepancy may be due to a number of factors, including a lack of cellular components to buffer Zn^2+^ or increased local Zn^2+^ concentration due to the presence of the histidine tag. The resolution of the hGlyRα3-100Zn structures is sufficient to visualize Zn^2+^ coordination in the high-affinity and low-affinity binding sites (Fig. 2F), which are visible in both the closed and desensitized states (Fig. 3, A and C). As seen in the 1 μM Zn^2+^ condition, (hGlyRα3-1Zn-Des), the potentiating site is coordinated by Glu^192^, Asp^194^, His^215^, and His^427^. However, the distance between Zn^2+^ and Glu^192^, as well as Zn^2+^ and His^427^, is shorter in the desensitized state compared to the closed state, suggesting a stronger coordination in this conformation. The twisting movement of the β9 and β10 strands (and associated inward movement of loop C) during glycine activation brings residues Glu^192^, Asp^194^, and His^215^ in an orientation conducive to Zn^2+^ coordination, consistent with the potentiating effects of this site (Fig. 3A). The low-affinity, inhibitory site is located inside the vestibule of the ECD. Although Zn^2+^ binding to the inhibitory site has never been observed in a GlyR structure, two residues thought to coordinate Zn^2+^ in GlyRα1 are His^109^ and His^107^. An H109F mutation in GlyRα1 has been shown to completely abolish Zn^2+^ inhibition, whereas a mutation of the neighboring His^107^ to asparagine greatly increased the median inhibitory concentration (IC_50_) of Zn^2+^ from 20 μM to greater than 3 mM (*32*). There was thought to be a third residue involved in inhibitory Zn^2+^ coordination because the dominant coordination geometry of Zn^2+^ by proteins is tetrahedral, and consequently, the binding sites typically incorporate at least three amino acid side chains and water (*78*–*80*). Although there are proteins that may be able to use only two residues along with water molecules (*81*), and this could explain why the GlyRα1 Zn^2+^ inhibition site has a relatively low binding affinity, it does not explain how Zn^2+^ is able to inhibit GlyRα3 at micromolar concentrations when it does not have a histidine at position 107. In wild-type (WT) GlyRα3, the position 107 is naturally occupied by asparagine, thereby contributing to an overall lower sensitivity of GlyRα3 to Zn^2+^ inhibition, but the IC_50_ of GlyRα3 has been determined to be around 150 μM in HEK cells, with the shift from potentiation to inhibition observable starting around 70 μM Zn^2+^ (*33*). The additional Zn^2+^-coordinating residue has been proposed to be Thr^112^, Thr^133^, or Glu^110^ (*33**,* *80*) as mutation of each results in attenuated inhibition, although this is possibly due to an allosteric effect. In addition to the proposed σ bond with the His^109^ nitrogen, we observe that, in the hGlyRα3-100Zn maps, Zn^2+^ is coordinated by an electrostatic interaction with Glu^103^ from the adjacent subunit (Fig. 2A). The residues involved in inhibitory Zn^2+^ coordination experience only a minor rearrangement in the transition from the closed to the desensitized state, with Glu^103^ shifting slightly away from His^109^ (Fig. 3C). The extension of Glu^103^ at the inhibitory site and the corresponding inhibitory Zn^2+^ density are not seen in the low Zn^2+^ condition (hGlyRα3-1Zn-Des) (fig. S9, B and C). Whereas His^107^ does play a role in coordinating Zn^2+^ for GlyRα1, there are additional residues involved that allow GlyRα3 to still be inhibited by micromolar concentrations of Zn^2+^. Specifically, we propose that Glu^103^ seems to be vital for Zn^2+^ coordination in the inhibitory site of GlyRα3.

**Fig. 4.**
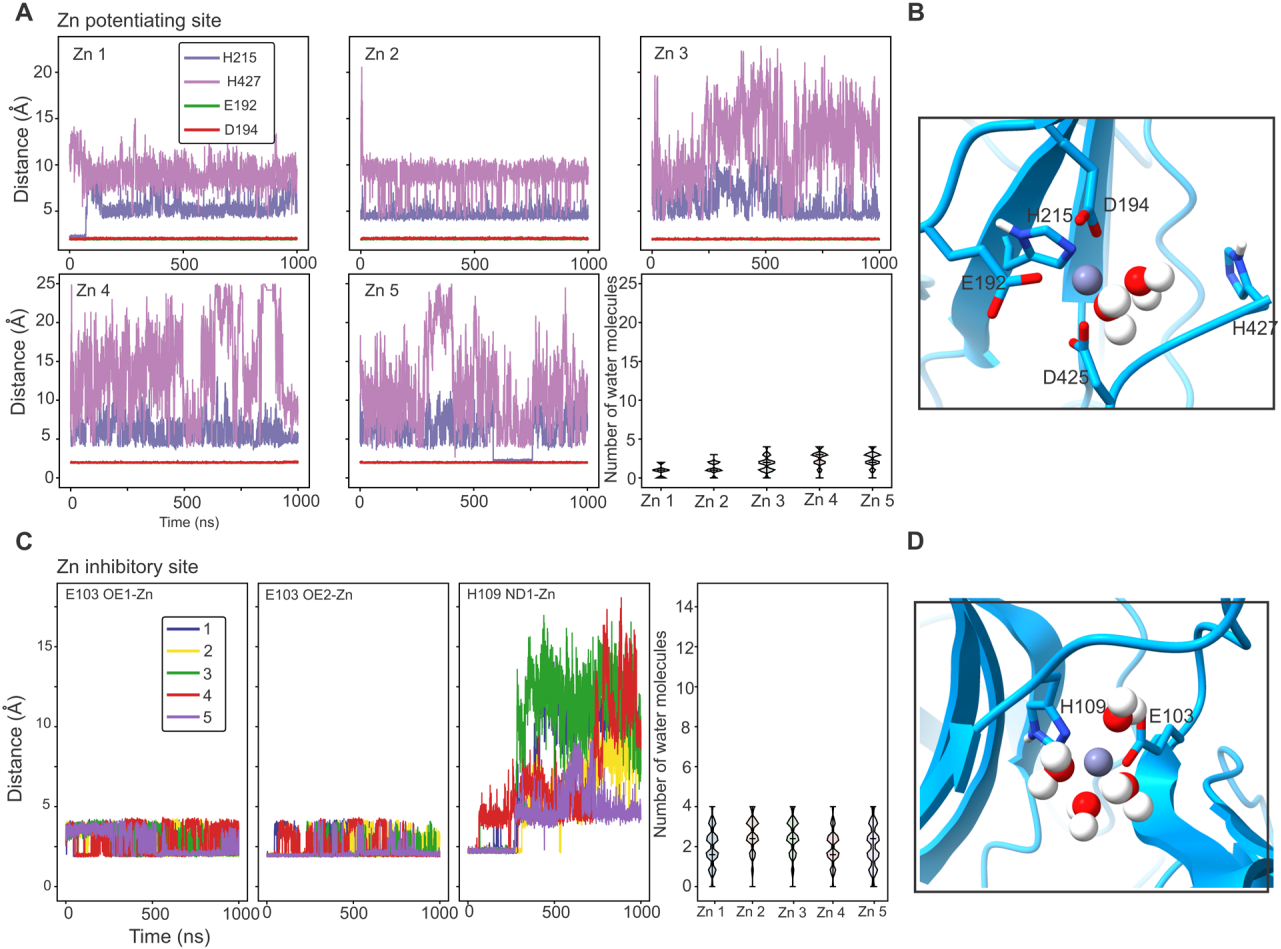
Assessment of stability of the Zn^2+^ coordination sites by MD simulations. We report the distance between Zn^2+^ and residues of the binding site as well as the minimal, maximal, and mean number of water molecules within 2.15 Å of Zn^2+^. (**A**) Zn^2+^ remains stably bound in the potentiation site during MD simulations. We evaluate the distance between Zn^2+^ and Glu^192^, Asp^194^, His^215^, and His^427^. Both histidines are neutral with the proton sitting on the δ nitrogen. We report the distance between Zn^2+^ and the ϵ nitrogen and the closest oxygen in the respective Glu or Asp residues. Glu^192^ and Asp^194^ coordinate Zn^2+^ in all five binding sites during the unrestrained 1000-ns–long MD simulation. His^215^ and His^427^ coordinate Zn^2+^ in only a few frames, whereas in most frames, Zn^2+^ interacts with two water molecules instead. (**B**) Simulation snapshot from the potentiation site of one subunit showing the final position of the Zn^2+^ coordination residues and water molecules within 2.15 Å. (**C**) Zn^2+^ remains stably bound in the inhibitory site during MD simulations. His^109^ is neutral with the proton sitting on the ϵ nitrogen, and the plot shows the distance between Zn^2+^ and the δ nitrogen of His^109^ and the oxygen (OE1 and OE2) of Glu^103^ to Zn^2+^. (**D**) Simulation snapshot from the inhibition site of one subunit showing the final position of the Zn^2+^ coordination residues and water molecules within 2.15 Å.

To assess the stability of the Zn^2+^ coordination sites, the hGlyRα3-100Zn-Des structure was embedded within a hydrated 1-palmitoyl-2-oleoyl-*sn*-glycero-3-phosphoethanolamine (POPE) lipid bilayer for MD simulations. Backbone restraints were applied for the first 50 ns to preserve the overall experimentally determined conformational state. This was then followed by 1000 ns runs without any restraints to allow full relaxation of the system. Zn^2+^ remains stably bound in both the potentiation site (Figs. 3B and 4A) and in the inhibition site (Figs. 3D and 4C) during the course of the simulations. Within the short MD simulations with backbone restraints, all four residues of the potentiating site, Glu^192^, Asp^194^, His^215^, and His^427^, coordinated Zn^2+^ tightly in three of the five runs. In the other two, the coordination of Zn^2+^ by His^215^ and His^427^ is lost and replaced by two water molecules (Fig. 3B). In the longer, unrestrained simulations, Zn^2+^ remains stably bound in all five of the subunits, primarily through association with Glu^192^ and Asp^194^ (Fig. 4A). The C-terminal end of the M4 helix appears to be highly flexible—the interaction with His^427^ occurs only transiently, and Zn^2+^ is coordinated by two water molecules via Asp^425^ on M4 (Fig. 4B). Similarly, at the inhibitory site, Zn^2+^ is stably coordinated through both the 50-ns backbone-restrained and 1000-ns unrestrained runs. The main interacting residues are Glu^103^ and His^109^, along with several water molecules. In the longer simulations, Zn^2+^ is stably associated with Glu^103^ but fluctuates in coordination with His^109^ and water molecules (Figs. 3D and 4, C and D).

**Fig. 5.**
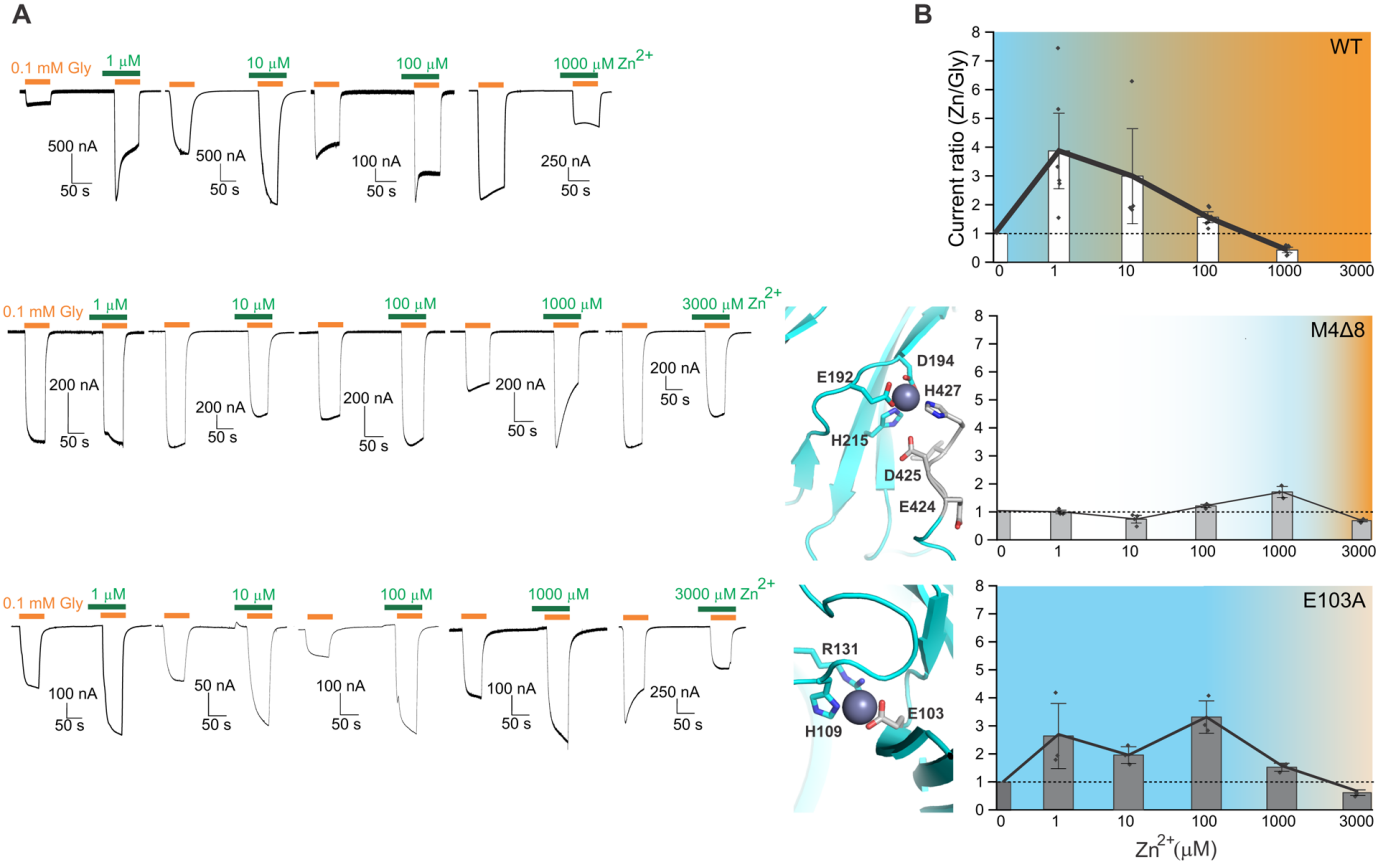
Mutations in the zinc coordination sites shift the balance between inhibition and potentiation. (**A**) Representative TEVC traces of glycine-activated and zinc-modulated currents from WT (top), M4Δ8 mutant (middle), and E103A mutant (bottom) GlyRα3. (**B**) Ratio of zinc modulated glycine currents to glycine only currents for WT GlyRα3 and the mutants in (A). Color corresponds to the degree of modulation, with blue and orange indicating potentiation and inhibition, respectively. Data from WT are the same as in Fig. 2. Current ratio for M4Δ8 at 1, 10, 100, 1000, and 3000 μM Zn^2+^ was 1.0 ± 0.04 (*n* = 4), 0.74 ± 0.09 (*n* = 4), 1.21 ± 0.04 (*n* = 4), 1.71 ± 0.13 (*n* = 3), and 0.69 ± 0.03 (*n* = 4), respectively. Current ratio for E103A at 1, 10, 100, 1000, and 3000 μM Zn^2+^ was 2.62 ± 0.8 (*n* = 3), 1.96 ± 0.2 (*n* = 3), 3.26 ± 0.4 (*n* = 4), 1.55 ± 0.1 (*n* = 3), and 0.57 ± 0.07 (*n* = 4), respectively.

For both Zn^2+^ modulation sites, we identified participation from additional residues that were not previously described through functional data. To evaluate their role, we carried out TEVC measurements on receptors with mutations at these positions. At the potentiating site, the post-M4 region (including the His^427^) is highly flexible during the course of longer simulations, yet Zn^2+^ remains stably bound through interactions with Glu^192^, Asp^194^, and water molecules (mediated through Asp^425^). The negatively charged residues in this region (Glu^424^, Asp^425^, and Asp^431^) could participate in Zn^2+^ coordination directly or through water interactions. Therefore, we deleted the last eight residues of this region, Glu^424^ to Asp^431^, to make the M4Δ8 mutant. This mutant was described previously as having a similar response to partial agonists as GlyRα1 (*72*). We found that the M4Δ8 mutation resulted in a remarkable loss of potentiation at low Zn^2+^ concentrations (1 or 10 μM) and a small level of potentiation at 1000 μM (~171 ± 13% of the amplitude of currents induced by glycine alone) (Fig. 5). A 10 μM Zn^2+^ ion elicits a small inhibitory response (~74 ± 9%), possibly due to a shift in *K*_d_ (dissociation constant) of the potentiating site to greater than that of the inhibitory site. Conversely, the E103A mutant exhibits a loss of Zn^2+^ inhibition extending up to 1000 μM. However, the peak level of potentiation appears lower for the E103A mutant than for WT. It is worth noting that this mutation in GlyRα1 leads to a decreased sensitivity for glycine, with the EC_50_ increasing from 230 to 430 μM (*82*).

### GlyRα3 is inhibited by acidic pH

**Fig. 6.**
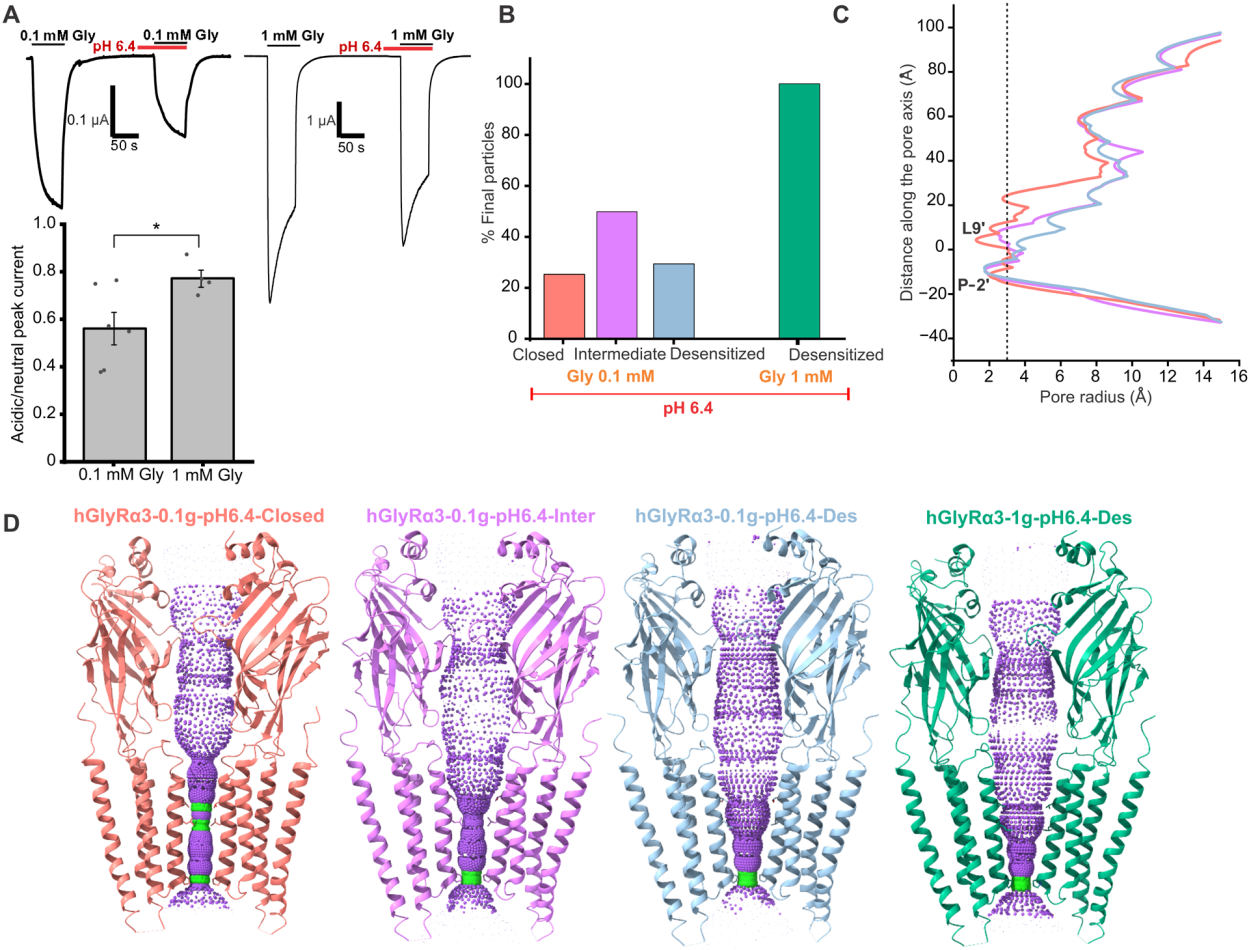
Acidic pH inhibits GlyRα3. (**A**) (Top) TEVC traces of glycine-activated currents in a physiological pH 7.5 solution and acidic pH 6.4 solution with 0.1 mM glycine (left) and 1 mM glycine (right). (Bottom) Mean ratio of glycine currents at pH 6.4 to pH 7.5 for 0.1 and 1 mM glycine. The mean 0.1 mM glycine current ratio is 0.56 ± 0.07 (*n* = 6), and the mean 1 mM glycine current ratio is 0.79 ± 0.04 (*n* = 4); *P = 0.037, *t* test. (**B**) Particle distribution of states for cryo-EM datasets collected at pH 6.4 with 0.1 and 1 mM glycine. (**C**) Plot of the pore radius for all four pH 6.4 maps. The radius of a hydrated chloride ion is indicated by a dotted line. (**D**) Hole profiles of the 0.1g-pH6.4 and 1g-pH6.4 states.

**Fig. 7.**
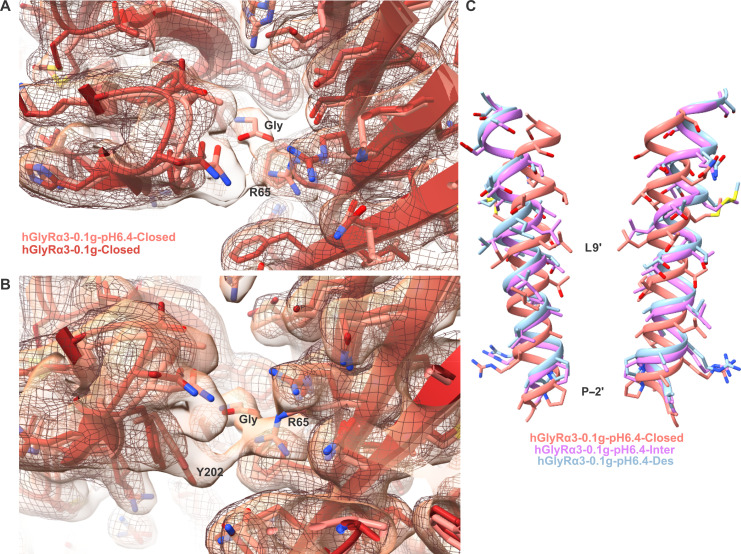
Agonist binding site and pore of 0.1 mM glycine pH 6.4 states. (**A**) Overlay of the glycine binding site of the 0.1 mM glycine apo state (red model, mesh map) and the 0.1 mM glycine pH 6.4 closed state (pink model, translucent map). There are two conformations of the Arg^65^ side chain in the pH 6.4 model. (**B**) Alternate view of (A), tilted up to emphasize the alternate conformation of Arg^65^ and the movement of Tyr^202^ toward the glycine. (**C**) Pore-lining M2 helices of two subunits showing the position of residues in the three 0.1g-pH6.4 states, with the activation and desensitization gates labeled.

GlyRα3 experiences substantial inhibition at acidic pH when activated with submaximal concentrations of glycine but is less affected at saturating concentrations of glycine (Fig. 6A). Consistent with the electrophysiology data, the particles subjected to cryo-EM analysis with 1 mM glycine at pH 6.4 resolved to a single conformation corresponding to that of the desensitized state (hGlyRα3-1g-pH6.4-Des, 2.8 Å) (figs. S12 and S13). This conformation is essentially identical to that determined for the 1 mM glycine at pH 8.0 condition (Fig. 6, B and D, and fig. S12). Conversely, at pH 6.4 with 100 μM glycine, we were able to resolve three states: desensitized, closed, and intermediate (Fig. 6 and figs. S14 and S15). The desensitized state (hGlyRα3-0.1g-pH6.4-Des, 2.2 Å) resembles all the other desensitized states described previously. The closed state (hGlyRα3-0.1g-pH6.4-Closed, 2.2 Å) differs from the other closed states in three ways, all related to the glycine binding site. Instead of an empty binding pocket, there is a strong glycine density (Fig. 7, A and B). In addition, the position of loop C is slightly closed, although not to the degree of the desensitized state. Last, there are two densities visible for Arg^65^, indicating that the Arg^65^ side chain alternates between two conformations—an upward conformation consistent with the apo state and a downward conformation typically seen in the activated state, which places it in position to form a hydrogen bond with the agonist glycine. With respect to residues that have been identified as necessary for the pH sensitivity of GlyRα3, we saw water densities in the vicinity of His^109^, Thr^133^, and Thr^112^ in the closed and desensitized states but no obvious change in the interaction network. His^215^ interacts with a post-M4 residue in all states, although the side chains are not well resolved due to the flexibility of this region. The most populated state classified at pH 6.4 with 100 μM glycine is a unique intermediate state (hGlyRα3-0.1g-pH6.4-Inter, 3.1 Å). In the binding pocket, the intermediate state is virtually identical to the desensitized state. However, along the pore, the intermediate state has an unexpected constriction at the Leu9′, although it is not as narrow as in the closed state (Fig. 6, C and D, and 7C).

To evaluate the flexibility of hGlyRα3-0.1g-pH6.4-Inter and verify that it was not simply a mixture of states or suboptimal particles, we performed 3D variability analysis (3DVA) using CryoSPARC (*83*). We examined three modes of movement of the receptor and divided the particles into 20 clusters to look for possible substates. We found clusters that appeared to resemble the closed, intermediate, and desensitized states (fig. S16 and movie S1). Upon refinement, the closed-like state had a slightly different position of loop C and the Leu9′ side chain was not visible, despite being visible in the intermediate-like cluster refinement, which was of similar quality. The desensitized-like state had a slight movement of the M2 helix and repositioning of loops in the ECD. Overall, these cluster substate maps were of lower resolution than that of the original intermediate state and were not considered informative enough to necessitate building separate models. These results do suggest heterogeneity in the intermediate state particles, but the consensus refinement is the highest resolution and therefore appears to be the best possible representation. We saw clusters that could have been an open/conducting state based on the expansion of the pore and the position of the Leu9′ side-chain density, but the subsequent refinements were also of relatively low resolution and more closely resembled the desensitized or intermediate states than a true open state. It is curious that none of the conditions investigated in this work resulted in an open conformation. This may be due to the transient nature of the open state, which would result in too few particles for a high-resolution structure to be achieved, or an asymmetry at Pro-2′ that causes it to become buried in another state such as hGlyRα3-0.1g-pH6.4-Inter. We cannot rule out the possibility that our reconstitution method is biasing the distribution toward a desensitized conformation, as it has been shown that factors such as scaffolding length and lipid composition of nanodiscs influences the degree of receptor activation (*84**,* *85*).

**Fig. 8.**
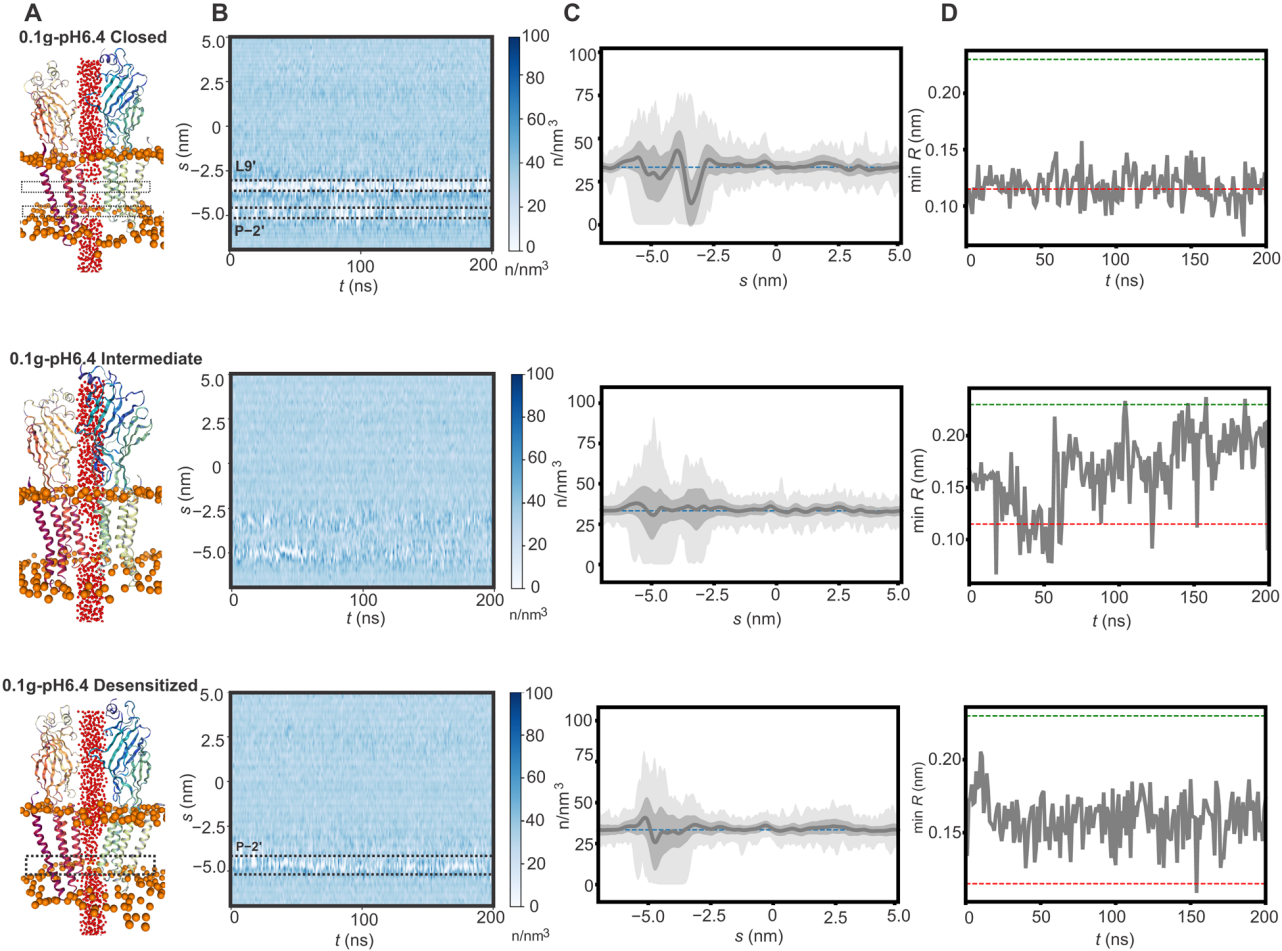
MD simulations to investigate the pore hydration profile of hGlyRα3-0.1g-pH6.4 states. (**A**) Snapshots from MD simulations for the closed, intermediate, and desensitized conformations (blue cartoon). Phosphate head groups of lipids are shown in orange, and water-oxygens are shown in red. (**B**) Time series of the water density along the pore axis (*s*) are plotted for a simulation time of 200 ns for each corresponding structure. (**C**) Each discrete water molecule position is associated with a Gaussian distribution, and the sum of all Gaussians yields the density function of water along the channel center line. The mean water density along the pore axis along with the corresponding SD in dark gray and minimum and maximum density in light gray (simulation average, SD, minimum, and maximum from 200 ns with a sampling interval of 0.1 ns). The dashed line represents the bulk water density of 33 nm^−3^. (**D**) Minimum pore radius over time. Red and green dashed lines represent the diameter of one or two water molecules, respectively.

Because the conformations observed at pH 6.4 were distinct from other GlyR structures, we assessed the conductance properties by MD simulations (Fig. 8A). The closed, intermediate, and desensitized states were embedded within a hydrated POPE lipid bilayer. Upon equilibration, backbone restraints were applied for 50 ns to preserve the overall experimentally determined conformational state, followed by 200 ns runs without any restraints except for His^311^ and Lys^386^ to account for the unstructured portion of the ICD. During the runs, all three pore conformations remained stable with no major changes to the overall pore radii besides fluctuations arising from side-chain movements of residues lining the pore. Water density was assessed for each of the states during the 200-ns simulations using a radius of 0.14 nm (Fig. 8B), and the time-averaged water density profiles along the channel axis were calculated (Fig. 8C). The closed conformation was clearly dewetted near Leu9′ with occasional dewetting also observed at Pro-2′ (Fig. 8, B and C, top panels). The desensitized state was fully hydrated at Leu9′ with only a narrow region of dewetting observed at Pro-2′ (Fig. 8, B and C, bottom panels). The water density profiles along the channel axis for these states are similar to those observed in other pore-wetting simulations carried out for GlyR structures of corresponding conformations (*62*). The intermediate state is hydrated throughout (Fig. 8, B and C, middle), differentiating it from the closed and desensitized states. However, the minimum pore radius for all three states during the simulations is below 2 Å, which is indicative of nonconductive conformations.

## DISCUSSION

**Fig. 9.**
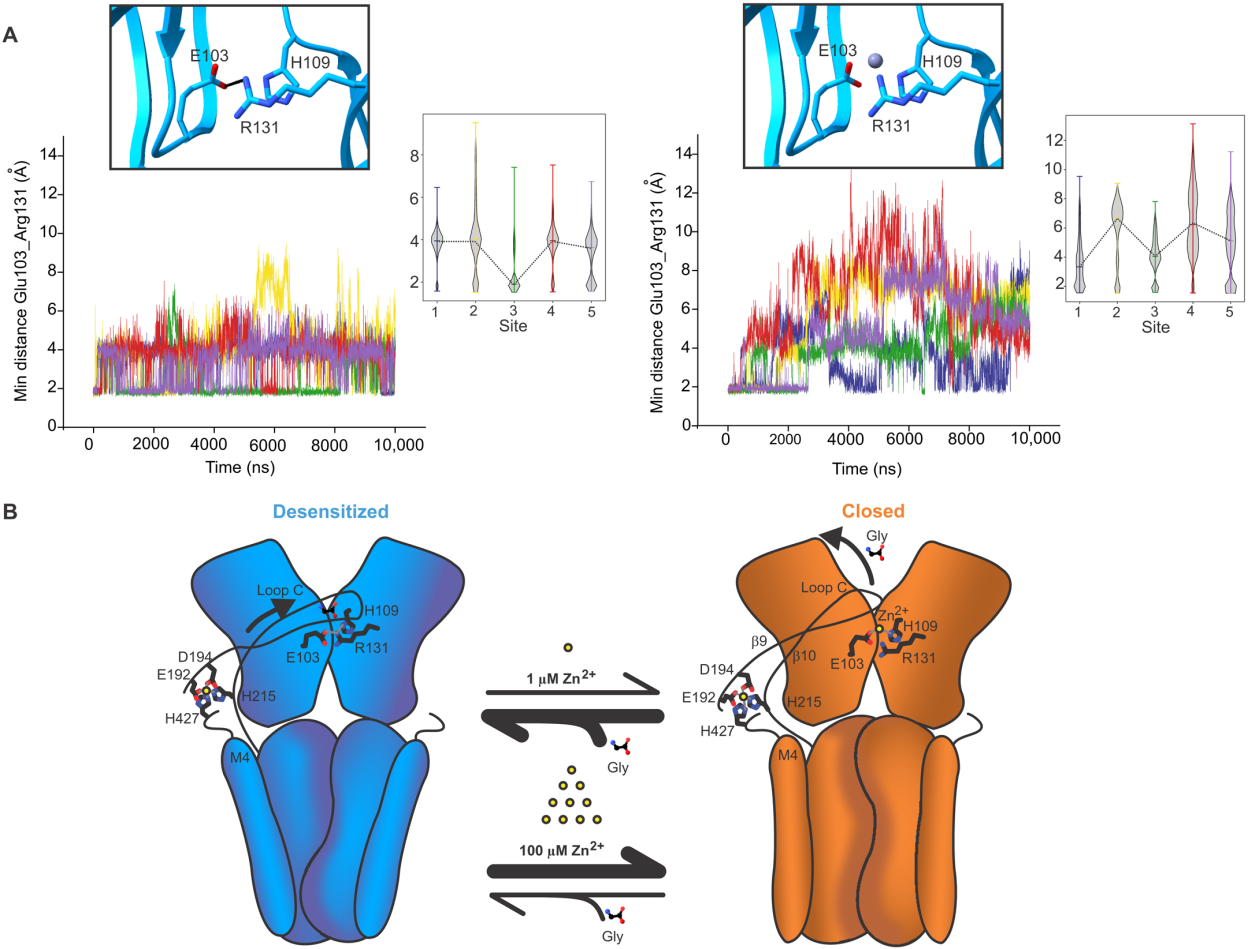
Disruption of salt bridge between Arg^131^ and Glu^103^ as a proposed mechanism of GlyRα3 modulation by Zn^2+^. (**A**) The minimal distance between Glu^103^ and Arg^131^ of neighboring subunits is plotted as a time series and aggregated (minimum, maximum, and mean). Traces for each of the subunits are shown as a different color. Zn^2+^ is removed from the inhibitory site and left intact at the potentiation site (left). Zn^2+^ is present in the inhibitory site but removed from the potentiation site (right). Inset shows the number of interacting water molecules at the inhibitory site. (**B**) (Left) At low concentrations, Zn^2+^ binds to the high-affinity, potentiating site formed by Glu^192^ and Asp^194^ on the β9 strand, His^215^ on the β10 strand, and His^427^ from the post-M4 segment. We propose that this interaction network stabilizes loop C in the glycine-bound conformation and promotes transition to the activated state. In the absence of Zn^2+^ binding at the low-affinity inhibitory site, there is a salt bridge between Glu^103^ and Arg^131^, as well as an interaction between Arg^131^ and His^109^ behind the glycine binding pocket. (Right) As the Zn^2+^ concentration increases, the occupancy of the low-affinity site increases and the coordination of Zn^2+^ by Glu^103^ and His^109^ disrupts the interaction with Arg^131^. Disruption of the E103-R131 salt bridge destabilizes glycine binding and promotes the closed/apo state.

Whereas a number of channels are modulated by Zn^2+^, the release of synaptic Zn^2+^ has been demonstrated to occur primarily from glutamatergic vesicles, suggesting that spillover to glycinergic synapses would not allow for GlyRs to experience high local Zn^2+^ concentrations. Nevertheless, there is evidence that glycine and Zn^2+^ colocalize in presynaptic terminals in the brainstem and spinal cord, allowing transient Zn^2+^ concentrations up to 10 μM (*34**,* *86**,* *87*). Therefore, under normal physiological conditions, GlyRs are mostly subjected to Zn^2+^ levels that are potentiating. Extracellular Zn^2+^ may become elevated under pathological conditions, wherein Zn^2+^-mediated GlyR inhibition through occupation of the low-affinity binding site could contribute to excitotoxic cell death mechanisms. The GlyR subunits show varying degrees of Zn^2+^ sensitivity, with GlyRα3 being the most sensitive to the potentiating effects. To some extent, this difference arises from His^107^ at the inhibitory site in GlyRα1, which is occupied by Asn^107^ in GlyRα2 and α3. An H107N mutation in GlyRα1 removes Zn^2+^ inhibition at higher concentrations (*32*), yet GlyRα3 is still inhibited by Zn^2+^ even with an asparagine side chain at this position. We find that Glu^103^ is instead directly involved in Zn^2+^ coordination along with His^109^ to allow for inhibition even without a second histidine available. Enhanced Zn^2+^ potentiation in GlyRα3 may also arise from greater stabilization of Zn^2+^ in the potentiating site. The M4 helix of GlyRα3 is two residues longer than that of GlyRα1, and a previous study has shown that transferring the C-terminal end of M4 from GlyRα1 or deletion of the last eight M4 residues imparts enhanced agonist sensitivity to GlyRα3 (*72*). It is notable that, in this chimeric channel, the efficacies of GlyR partial agonists are also greatly increased, turning them into full agonists, which highlights the role of the post-M4 region in modulating ligand activation. We posit that Zn^2+^ coordination involving the β9 strand (Glu^192^ and Asp^194^), β10 strand (His^215^), and post-M4 (via His^427^) could allow for M4 to stabilize the closed conformation of loop C after agonist binding, promoting an activated state (Fig. 9). It has previously been suggested that, based on single-channel recordings of GlyRα1, potentiating levels of Zn^2+^ decrease glycine dissociation rates (and hence increase agonist affinity and mean open times) (*77**,* *88*). We observe that, at high concentrations, Zn^2+^ additionally occupies the low-affinity site coordinated by Glu^103^ and His^109^ from adjacent subunits. The side chain of Glu^103^ typically forms a salt bridge with Arg^131^ from the neighboring subunit, and in the desensitized state, Arg^131^ forms an additional interaction with His^109^ (within the same subunit). The Glu^103^-Arg^131^-His^109^ interaction is disrupted by Zn^2+^ coordination at this site and might be the basis for the inhibitory effect of Zn^2+^ (Fig. 9A). Consistent with this idea, in the MD simulations with Zn^2+^ bound at the inhibitory site, the minimum distance between Glu^103^ and Arg^131^ from the neighboring subunit increases, indicating a weakened salt bridge interaction between the two side chains. However, in the simulations with Zn^2+^ removed from the inhibitory site, Glu^103^ and Arg^131^ remain in close contact (Fig. 9A). The critical role of the Glu^103^-Arg^131^ interaction in GlyR function is underscored by the loss of function phenotype of E103K for GlyRα1, which causes hyperekplexia (*82*). In addition, the R131A mutation completely abolishes Zn^2+^ inhibition even at very high concentrations of 1000 μM. This mutation also turns GlyRα1 into a Zn-activated channel with an EC_50_ of ~1 μM (*89*). Our future studies will focus on investigating the effects of Zn^2+^ modulation on GlyRs with extensive MD simulations and also looking at different models of Zn^2+^ (*79*) to characterize properties such as binding affinity extending beyond the simple analysis performed here.

Understanding the structural changes experienced by GlyRα3 under acidic conditions provides insight into the receptor’s behavior in pathological pain states. In contrast to what was seen with the inhibitory 100 μM Zn^2+^ dataset, the particles of the acidic pH dataset populated distinct conformational states. It is worth noting that, at pH 6.4 and 0.1 mM glycine, the closed state was not the most populated state. Instead it was the intermediate (likely nonconducting) conformation. In addition, we found that glycine was present in the closed state when solved at pH 6.4 with 0.1 mM glycine, which was different from all other datasets. This could be attributable to a reduced sensitivity of the receptor for glycine, as agonist-bound closed states of GlyRs have been found for the partial agonist taurine (*60*), although a previous study reports no significant change in the Hill coefficient of GlyRα1 at pH 5.5 (*47*). Perhaps the presence of glycine in the closed state is due to an increase in activation time (*50*) at acidic pH. Another unique aspect of the acidic pH condition was the presence of the intermediate state, which resembles the desensitized state at the level of the agonist binding site but has a pore that is intermediate between the closed and desensitized states. Despite being the most prevalent state in the dataset, the 0.1g-pH6.4-Inter state was of the lowest resolution, suggesting a high degree of flexibility. The 3DVA revealed movements in the TMD and ECD corresponding to the changes that occur when the receptor transitions from an inactivated to activated state. There is evidence to suggest that the dissociation rate of protons from GlyRs is faster than that of glycine (*47*), so while protonation may precede glycine binding and prevent its transition into an open state, receptors locked in a nonconducting or semiactivated state may be able to rapidly sample an activated state due to the presence of glycine and the relatively transient interaction with protons.

Proper nervous system function requires a delicate balance of excitatory and inhibitory signaling, through receptors that themselves experience complex positive and negative regulation. Here, we have demonstrated how physiological changes in the extracellular environment can regulate GlyRα3 through subtle conformational changes. Although synaptic GlyRs should be exclusively αβ heteromers given that synaptic localization requires the scaffolding protein gephyrin, which only interacts with the β subunit (*90**,* *91*), homomeric GlyRs may play a role in tonic modulation of membrane potential extrasynaptically (*92*–*94*) and regulation of neurotransmitter release presynaptically (*7*). Regardless, GlyRs that contain α3 subunits contribute to pain perception, and studying modulation mechanisms may prove useful for the development of analgesic drugs. Because of the high homology between α subunits, finding highly selective PAMs has remained an ongoing challenge (*5**,* *95**,* *96*). Future exploration of pharmacological methods of GlyRα3 modulation through cryo-EM may allow for the determination of structural motifs that can be exploited in drug design.

## METHODS

### Electrophysiological recordings by TEVC

The pCS2+-α3 constructs for expression in *X. laevis* oocytes were cloned and synthesized by GenScript (table S1). DNA was linearized using the Sap I restriction enzyme, which was incubated at 37°C for 3 hours. The mMessage mMachine SP6 kit (Ambion) was used to transcribe the RNA from the linearized DNA, as per the manufacturer’s instructions. The RNA was then purified with an RNeasy kit (Qiagen). *X. laevis* oocytes (stages V to VI) were injected with 2 to 48 ng of RNA, and currents were measured 1 to 3 days after injection. Water-injected oocytes were used as a negative control to assess oocyte health and verify that no endogenous currents were present. The oocytes used in this study were purchased from Ecocyte and also provided courtesy of W. F. Boron. Female *X. laevis* were purchased from Nasco. We adhered to all relevant ethical regulations for animal testing and research. Animal experimental procedures were approved by the Institutional Animal Care and Use Committee of Case Western Reserve University (CWRU). Oocytes were incubated at 18°C in a frog Ringer’s solution (96 mM NaCl, 2 mM KCl, 1 mM MgCl_2_, 1.8 mM CaCl_2_, and 20 mM Hepes) supplemented with 2.5 mM sodium pyruvate, gentamicin (50 μg/ml), and tetracycline (100 μg/ml). The pH was adjusted to 7.5, and the osmolarity of the solution was adjusted to 195 ± 5 mosmol.

TEVC experiments were performed on an Axon Instruments Axoclamp 900A. Currents were sampled and digitized at 500 Hz with an Axon Digidata 1550B and analyzed by Clampfit 10.7.0.3 (Molecular Devices). Oocytes were clamped at a holding potential of −60 mV, and solutions were exchanged using a syringe perfusion system flowing at a rate of ~10 ml/min. The ND96 solution used while recording consisted of 96 mM NaCl, 2 mM KCl, 1.8 mM CaCl_2_, 1 mM MgCl_2_, and 20 mM Hepes (neutral pH) or 20 mM MES (acidic pH). The pH was adjusted to 7.5 for all experiments not pertaining to pH modulation and pH 6.4 when testing the effects of acidity. The osmolarity was adjusted to 195 ± 5 mosmol. Glycine and zinc chloride were purchased from Sigma-Aldrich. Current traces were plotted using Origin 2023b. Statistical analysis was performed using Origin. All data are reported as means ± SE for (*n*) individual oocytes unless otherwise stated. No sample size calculation was made. All statistical tests were unpaired and two-sided. The glycine concentration–response data were fit to the following equation in Origin: *I*/*I*_max_ = [glycine]*^n^*/(EC_50_*^n^* + [glycine]*^n^*), where EC_50_ is the concentration at which the current is half-maximal and *n* is the Hill coefficient.

### Full-length GlyRα3 cloning and transfection

The full-length codon-optimized human GlyRα3 gene (*GLRA3*, UniProt ID: O75311) was synthesized and subcloned into a pFastBac1 vector by GenScript. The construct includes a thrombin cleavage sequence (LVPRGS) and a C-terminal 8x histidine tag (table S2). For making a recombinant baculovirus, *S. frugiperda* ExpiSf9 cells grown in an ExpiSf CD Medium (Gibco, A35243) were transfected with recombinant GlyRα3 bacmid using ExpiFectamine (Gibco) according to the product manual. After 3 to 5 days, the GlyRα3 P0 baculovirus was collected and cell debris was removed by centrifugation at 300*g* for 5 min. The Sf9 cells used for virus propagation were cultured in an Sf-900 II SFM medium (Gibco, 11496015) and infected with P0 virus at an multiplicity of infection (MOI) of around 0.1 virions per cell when the cell density was ~2 × 10^6^/ml. The resulting P1 virus was then harvested after 48 hours via centrifugation at 1000*g* for 5 min. All Sf9 cells were cultured without antibiotics or CO_2_ exchange at 28°C.

### GlyRα3 expression, purification, and peptidisc incorporation

For GlyRα3 protein expression, Sf9 cells were infected with P1 virus at a cell density of 1.8 × 10^6^ to 2.2 × 10^6^/ml, MOI ≤ 0.1 virions per cell. After 48 hours, the cells were centrifuged at 8000g for 10 min at 4°C and the cell pellet was resuspended in a lysis buffer [50 mM tris-HCl and 36.5 mM sucrose (pH 8.0)] supplemented with 0.1% protease inhibitor cocktail and DNAse (0.05 mg/ml; Sigma-Aldrich). Sonication was used to lyse cells, and unlysed cells were removed by centrifugation at 3000*g* for 10 min. The supernatant containing lysed cells was spun down in an ultracentrifuge at 167,000*g* for 30 min, and the pellet containing the cell membrane fraction was resuspended in a buffer containing 20 mM Hepes and 150 mM NaCl at pH 8.0 (buffer A) supplemented with 10% glycerol. Membranes were flash frozen in liquid nitrogen before solubilization. An anti-histidine tag antibody (Thermo Fisher Scientific, MA1-21315) was used to confirm GlyRα3 expression by Western blot.

Membranes were solubilized using 10 mM *n*-dodecyl-β-d-maltopyranoside (DDM, Anatrace) with asolectin (0.05 mg/ml; soybean polar extract, Avanti Polar Lipids) and 0.1% cholesteryl hemisuccinate (CHS) (Anatrace) for 2 hours at 4°C. The solubilized protein was isolated from membranes by centrifugation at 167,000*g* for 15 min. The supernatant was then incubated with TALON resin that had been preequilibrated with buffer A, 1 mM DDM, asolectin (0.05 mg/ml), and 0.01% CHS for 45 min at 4°C. After incubation with beads, the flow-through was drained and the beads were washed with 10 column volumes of a low-detergent buffer containing 0.5 mM DDM, asolectin (0.05 mg/ml), and 0.01% CHS. Commercially purchased NSP peptide (Peptidisc Lab) was solubilized in 20 mM Tris buffer to a concentration of 1 mg/ml and pH adjusted to ~8.0. The TALON resin was incubated with 3 bed volumes of peptidisc (1 mg/ml) at 4°C for 30 min. Excess NSP was drained, and then beads were washed with 5 column volumes of buffer A and 50 mM imidazole without any detergent or lipids. Protein was eluted in buffer A with 250 mM imidazole. The eluted protein was concentrated using a 50-kDa Millipore filter (Amicon), and the concentrated protein was passed through a 0.22-μm centrifuge filter before size exclusion chromatography. Size exclusion chromatography of the protein was performed using a Superose 6 Increase column (GE HealthCare) equilibrated with buffer A without detergent. For the pH 6.4 samples, the Superose 6 column was equilibrated with buffer A adjusted to pH 6.4. Fractions corresponding to the mass of pentameric GlyRα3 at ~15 ml were collected and concentrated for cryo-EM.

### Preparation of the sample for cryo-EM imaging and parameters for data acquisition

GlyRα3 in peptidisc was concentrated to 0.3 to 1 mg/ml for cryo-EM grid preparation. For all datasets, glycine was added 10 min before freezing. Zinc chloride was added immediately after glycine. Cu 300 mesh Quantifoil R 1.2/1.3 grids (Quantifoil MicroTools) were glow discharged at 25 to 35 mA for 1 min (Emitech K100X, Quorum) for grids prepared at CWRU. For 1 mM glycine grids prepared at the New York Structural Biology Center (NYSBC), grids were plasma cleaned using H_2_ and O_2_ at 50 W for 30 s (Solarus II, Gatan). Protein sample was double blotted on the grid, 3.5 μl per blot. Blot time was 3.5 to 4.5 s, and wait time was 10 s. Grids were blotted and plunge frozen into liquid ethane using a Vitrobot Mark IV (FEI). Imaging of grids was performed using a 300-keV FEI Titan Krios microscope equipped with a Gatan K3 direct electron detector camera and Gatan Imaging Filter for all datasets except 1g-pH6.4, for which a 200-keV Glacios microscope equipped with a Falcon 4 camera was used. Data collection was set up with the EPU (CWRU), Leginon (NYSBC), or SerialEM [CWRU and Pacific Northwest Center for Cryo-EM (PNCC)] software. Data acquisition parameters are subsequently listed for each dataset individually.

hGlyRα3-0.1g: 10,986 super-resolution movies were collected at ×81,000 magnification, which gives a physical pixel size of 1.1 Å/pixel (CWRU, Krios). Movies were collected with 50 frames at a total dose of 62 e^−^/Å^2^. Defocus range was −0.75 to −1.50 μm.

hGlyRα3-1g: 6434 super-resolution movies were collected at ×81,000 magnification, which gives a physical pixel size of 1.069 Å/pixel (NYSBC, Krios #6). Movies were collected with 50 frames at a total dose of 65 e^−^/Å^2^. Defocus range was −0.75 to −1.75 μm.

hGlyRα3-100Zn: 6948 super-resolution movies were collected at ×105,000 magnification, which gives a physical pixel size of 0.84 Å/pixel (CWRU, Krios). Movies were collected with 50 frames at a total dose of 61 e^−^/Å^2^. Defocus range was −0.75 to −1.50 μm.

hGlyRα3-1Zn: 8458 super-resolution movies were collected at ×105,000 magnification, which gives a physical pixel size of 0.8266 Å/pixel (PNCC, Krios #2). Movies were collected with 50 frames at a total dose of 60 e^−^/Å^2^. Defocus range was −0.8 to −2.5 μm.

hGlyRα3-0.1g-pH6.4: 17,714 super-resolution movies were collected at ×105,000 magnification, which gives a physical pixel size of 0.84 Å/pixel (CWRU, Krios). Movies were collected with 50 frames at a total dose of 62 e^−^/Å^2^. Defocus range was −0.75 to −2.0 μm.

hGlyRα3-1g-pH6.4: 2461 super-resolution movies were collected at ×130,000 magnification, which gives a physical pixel size of 0.91 Å/pixel (CWRU, Glacios). Movies were collected with 1498 electron-event representation (EER) frames at a total dose of 60 e^−^/Å^2^. Defocus range was −0.8 to −2.2 μm.

### Image processing

Beam-induced motion was corrected using MotionCor2 (v1.4.2) with a B-factor of 150 pixels. Super-resolution images were binned (2 × 2) in Fourier space. Data processing was conducted in RELION 3.1 & 4.0. The Gctf software was used for contrast transfer function (CTF) estimation. A similar approach was followed for processing of all datasets. Micrographs were curated based on the following criteria: estimated defocus value, astigmatism, estimated maximum resolution, and CTF figure of merit. The best quality micrographs were then used for template-based picking. An initial template was generated from 2D classes of a preliminary GlyRα3 dataset. After autopicking, particles were subjected to 2D classification to remove low-quality particles and false picks. After two to three rounds of 2D classification, an initial map was generated via 3D autorefinement, using a 20 Å low-pass–filtered GlyRα1 map as an initial template, and then later using GlyRα3 maps from prior datasets. The 3D refinement underwent 3D classification with image alignment to sort out broken or poorly aligned particles, and the best classes were refined again and subjected to sharpening/postprocessing, CTF refinement, and Bayesian polishing. Parameters for Bayesian polishing were determined by training with 1000 particles. The polished particles were then used for 3D classification without image alignment (*T* = 12) to sort out different conformations of the receptor. If multiple states were found, then further rounds of 3D classification with larger *T* values were conducted for each state to probe for potential substates. The particle sets then underwent multiple rounds of CTF refinement, Bayesian polishing, and 2D classification without image alignment. All of the final maps presented were refined while imposing C5 symmetry. Refinement in C1 was performed at some point for each dataset to check for notable functional asymmetry. The 1 mM glycine dataset was initially processed in C1 and subjected to symmetry expansion to assess partial occupancy of glycine. RELION’s own implementation was used to determine local resolution of the final maps.

#### hGlyRα3-0.1g

General data processing strategy was as described above. From 10,986 super-resolution movies, 7957 micrographs were selected after CTF estimation. Initially, 3,914,212 particles were picked. After several rounds of 2D and 3D classification, a total of 99,442 particles were used for the final apo state 3D refinement and 106,791 particles for the desensitized state refinement. The final global resolution was determined to be 2.5 Å for the apo/closed and 2.6 Å for the desensitized state [Fourier shell correlation (FSC) = 0.143], with local resolutions varying from 2.3 to 3.3 Å and 2.5 to 3.3 Å, respectively.

#### hGlyRα3-1g

General data processing strategy was as described above. From 6434 super-resolution movies, 5050 micrographs were selected after CTF estimation. Initially, 2,874,967 particles were picked. After several rounds of 2D, a total of 435,078 particles were used for the 3D refinement of the desensitized state, which was the only distinct state found in this dataset. The final global resolution was determined to be 2.8 Å (FSC = 0.143), with local resolutions varying from 2.5 to 5.5 Å.

#### hGlyRα3-100Zn

General data processing strategy was as described above. From 6948 super-resolution movies, 5261 micrographs were selected after CTF estimation. Initially, 1,447,097 particles were picked. After several rounds of 2D and 3D classification, a total of 154,787 particles were used for the final apo state 3D refinement and 32,894 particles for the desensitized state refinement. The final global resolution was determined to be 2.2 Å for the closed and 2.5 Å for the desensitized state (FSC = 0.143), with local resolutions varying from 2.1 to 5 Å and 2.4 to 6 Å, respectively.

#### hGlyRα3-1Zn

General data processing strategy was as described above. From 8458 super-resolution movies, 6246 micrographs were selected after CTF estimation. Initially, 2,008,979 particles were picked. After several rounds of 2D, a total of 21,478 particles were used for the 3D refinement of the desensitized state, which was the only distinct state found in this dataset. The final global resolution was determined to be 2.9 Å (FSC = 0.143), with local resolutions varying from 2.7 to 7.4 Å.

#### hGlyRα3-0.1g-pH6.4

General data processing strategy was as described above. From 17,714 super-resolution movies, 15,072 micrographs were selected after CTF estimation. Initially, 8,344,616 particles were picked. After several rounds of 2D and 3D classification, a total of 132,119 particles were used for the final closed state 3D refinement, 165,143 particles for the desensitized state refinement, and 283,814 for the intermediate state refinement. The final global resolution was determined to be 2.2 Å for both the closed and desensitized state and 3.1 Å for the intermediate state (FSC = 0.143), with local resolutions varying from 2.1 to 4.2 Å and 2.8 to 5.2 Å, respectively.

#### hGlyRα3-1g-pH6.4

General data processing strategy was as described above. From 2461 super-resolution movies, 2020 micrographs were selected after CTF estimation. Initially, 1,281,017 particles were picked. After several rounds of 2D, a total of 100,328 particles were used for the 3D refinement of the desensitized state, which was the only distinct state found in this dataset. The final global resolution was determined to be 2.9 Å (FSC = 0.143), with local resolutions varying from 2.7 to 6.2 Å.

### 3D variability analysis

Particles used for the C5 reconstruction of the intermediate state were polished in RELION and then imported to CryoSPARC v4.4.1. The particles were downsampled and symmetry expanded before 3DVA (*83*). For the 3DVA job, the micelle was masked out and the results were filtered at 4 Å to reduce the contribution of high-frequency noise. Three modes of movement were solved. Mode 0 included expansion of the TM helices, ECD twisting, and closure of loop C (movie S1). Mode 1 showed rotation of the top portion of M2 and Leu9′. Mode 2 captured the movement of Pro-2′ into and out of the pore. Because all three modes represent changes that occur during the transition from the apo to desensitized state, all three were used to split the particles into 20 clusters. Clusters resembling the symmetric closed, intermediate, and desensitized states could all be seen, as well as clusters that were difficult to assign due to ambiguous density, asymmetry, or having features that did not precisely fall into the three main states. A representative cluster resembling each of the three main states (apo, desensitized, and intermediate) was selected and used for nonuniform refinement to assess similarity to the states that were solved using the entire dataset. Maps derived from 3DVA clusters were not subjected to further refinement or postprocessing, and models were not built as they were only used to evaluate heterogeneity in the intermediate state.

### GlyRα3 model building

Unsharpened maps were used for model building, which contain density for the full ECD, TMD, and some ICD. A truncated human GlyRα3 crystal structure (Protein Data Bank ID: 5TIO) was used as the starting model for 0.1g-Des. The 0.1g-Des model was used to build 0.1g-Apo. The initial model for each dataset is included in tables S1 to S3. The residue numbers correspond to the sequence in the UniProt database without the signal sequence (O75311). Some residues were not modeled for M4 and M3 if map density was too poor. Initial model building was done in Coot (v0.8.9.1) and WinCoot (v0.9.8.92), and then real space refinement was performed in PHENIX (phenix.real_space_refinement) to refine the model to the map using rigid body, local grid search, noncrystallographic symmetry, and gradient minimization. The models were then refined iteratively in Coot and PHENIX. The refinement statistics, the final model to map cross-correlation, and the stereochemical properties of the models, assessed by MolProbity, are included in tables S1 to S3. The HOLE program was used to calculate hole profiles. Figures were prepared using ChimeraX (UCSF, v1.11), Pymol (Schrödinger, v2.5.5), and CorelDraw (CorelDraw 2021).

### MD simulations

Cryo-EM structures of the apo, desensitized, and intermediate state were embedded within phospholipid (POPE) bilayer membranes with the CHARMM-GUI Membrane Builder (*97**,* *98*) in simulation cells (10 nm by 10 nm by 16.6 nm). The cryo-EM structures are missing a loop between TM3 and TM4 corresponding to Lys^312^-Arg^385^. Rather than build this in randomly, we elected to simply restrain the terminal residues His^311^ and Lys^386^ with 500 kJ/mol per nm^2^. Simulations were performed with GROMACS (2021) (*99*), using the TIP3P water model (*100*) and the CHARMM36m force field (*101*). The integration time step was 2 fs. Bonds were constrained through the LINCS algorithm (*102*) implemented in GROMACs (2021). A Verlet cutoff scheme was applied, and long-range electrostatic interactions were calculated using the particle mesh Ewald method (*103*). Temperature and pressure were maintained at 310 K and 1 bar during simulations, using the velocity-rescaling thermostat (*104*) in combination with a semi-isotropic Parrinello and Rahman barostat (*105*), with coupling constants of 1 and 5 ps, respectively. After energy minimization and several shorter equilibration simulations (see table S4) in the constant-temperature, constant-volume (NVT) and constant-temperature, constant-pressure (NPT) ensemble, backbone restraints were applied for 50 ns to preserve the overall experimentally determined conformational state. Steps 1 to 5 of the equilibration in table S4 were performed sequentially. The final frame of the previous step was used as the initial condition for the subsequent step. After this 50-ns restrained run, each system was simulated for a further 200 ns without any restraints, except for the restraints designed to keep the ends of the missing region at the original distances (His^311^ and Lys^386^; see step 6 in table S4). Pore water–free energy profiles were computed for alternative conformations of the protein using the Channel Annotation Package (*106*), in each case based on 200-ns equilibrium simulations without any restraints at physiological salt (150 mM NaCl) concentration. Simulation trajectories were analyzed at 100 ps intervals, with a bandwidth of 0.14 nm applied for water density estimation.

We evaluated the stability of the Zn^2+^ binding sites and the salt bridge between Glu^103^ and Arg^131^ by running unrestrained simulations for 1000 ns after the equilibration protocol (step 7 in the table). In the initial structure, there are five Zn^2+^ bound to the inhibition site and five Zn^2+^ bound to the potentiation site. We ran simulations with all 10 Zn^2+^ present, Zn^2+^ only at the potentiation site, Zn^2+^ only at the inhibition site, and with all Zn^2+^ deleted. We analyzed the salt bridge between Glu^103^ and Arg^131^, the coordination of Zn^2+^ every 200 ps, and the number of water molecules within 2.15 Å of Zn^2+^. For the potentiation site, we evaluated the distance between Zn^2+^ and Glu^192^, Asp^194^, His^215^, and His^427^. Both histidines were neutral with the proton sitting on the δ nitrogen. We report the distance between Zn^2+^ and the ϵ nitrogen in the histidines and the closest oxygen in the respective Glu or Asp residues. For the inhibitory site, His^109^ is neutral with the proton sitting on the ϵ nitrogen and we plot the distance between Zn^2+^ and the δ nitrogen of His^109^ and the oxygen (OE1 and OE2) of Glu^103^ to Zn^2+^.
