# Supplementary material for: Mechanisms underlying modulation of human GlyRα3 by Zn2+ and pH
Source: Sci Adv. 2024 Dec 18;10(51):eadr5920. doi: 10.1126/sciadv.adr5920 (PMC11654702; doi:10.1126/sciadv.adr5920)
Supplement: Supplementary file 1 — Figs. S1 to S16 Data S1 and S2 Tables S1 to S4 Legend for movie S1 [file sciadv.adr5920_sm.pdf]

Science Advances
AAAS

Supplementary Materials for

Mechanisms underlying modulation of human GlyRα3 by Zn^{2+} and pH

Kayla Kindig *et al.*

Corresponding author: Sudha Chakrapani, sudha.chakrapani@case.edu

Sci. Adv. 10, eadr5920 (2024)
DOI: 10.1126/sciadv.adr5920

The PDF file includes:

Figs. S1 to S16
Data S1 and S2
Tables S1 to S4
Legend for movie S1

Other Supplementary Material for this manuscript includes the following:

Movie S1

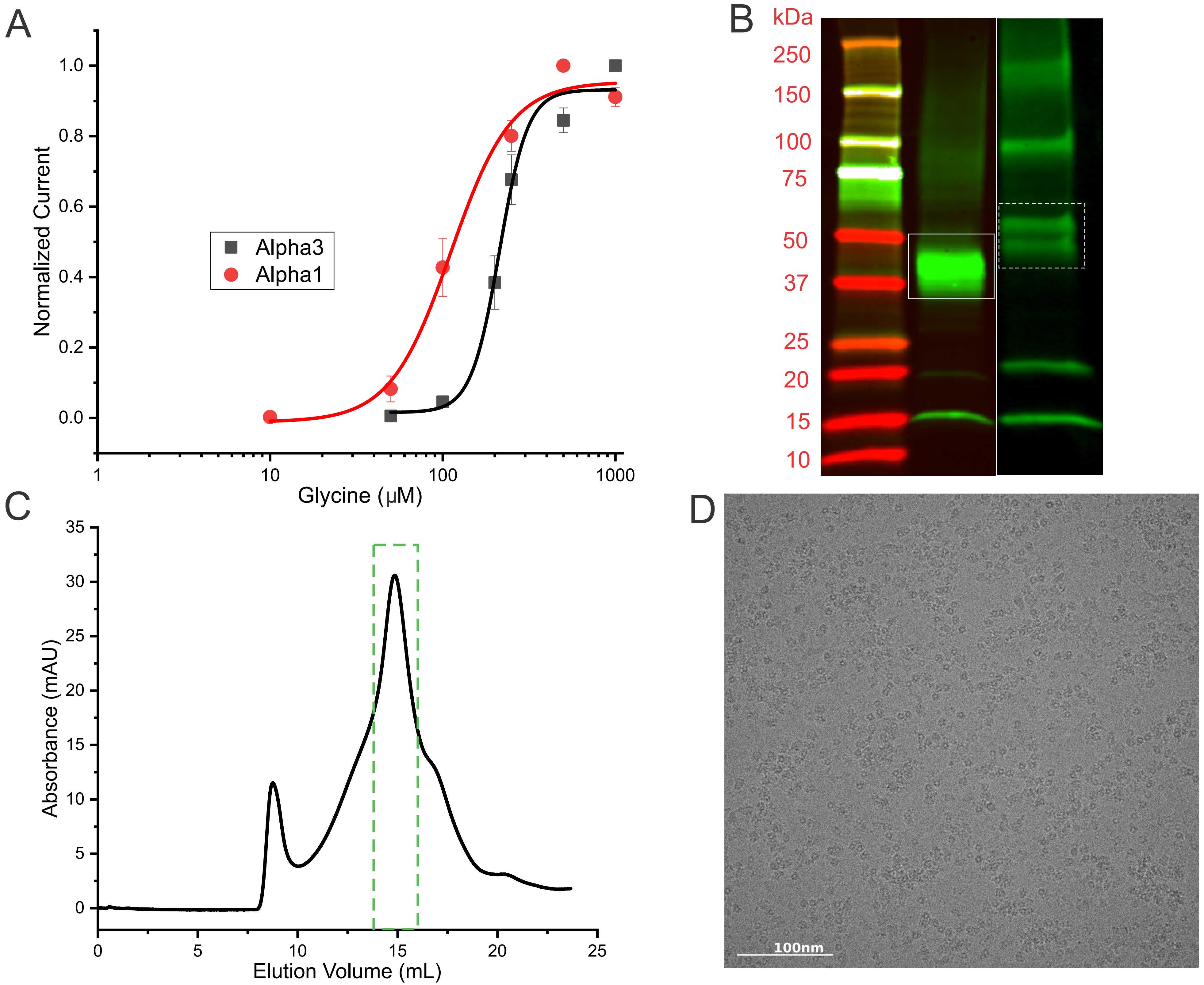
A
1.0
0.8
0.6
0.4
0.2
0.0
Normalized Current
Alpha3
Alpha1
1
10
100
1000
Glycine (μM)
B
kDa
250
150
100
75
50
37
25
20
15
10
C
35
30
25
20
15
10
5
0
Absorbance (mAU)
0
5
10
15
20
25
Elution Volume (mL)
D
100nm

Figure S1. Expression and purification of full-length human GlyRα3 for Cryo-EM anlysis. (A) Dose-response glycine curve of GlyRα3 and GlyRα1 measured by TEVC. Points are mean ± standard error from individual oocytes and normalized to the peak current. The EC_{50} for glycine is 213 ± 13 µM (n=3) for GlyRα3 and 111± 15 µM for GlyRα1 (n=7). (B) Western blot using a primary antibody against the 8x histidine tag of GlyRα3. Left lane sample is from solubilized Sf9 membranes, with a white box around the monomer band. Right lane is from affinity purified and concentrated protein in peptidisc after size exclusion chromatography, with a dotted white box around the monomer band. The appearance of a double band may be attributable to two different glycosylation states. (C) Size exlusion chromatogram after affinity purification of GlyRα3 in peptidisc. The green box indicates the pentamer peak at ~15mL elution volume, and this is what was concentrated and loaded on the third lane of the Western blot in part (A). (D) Micrograph from a Titan Krios at 81kx showing the purified GlyRα3 sample on a Quantifoil grid.

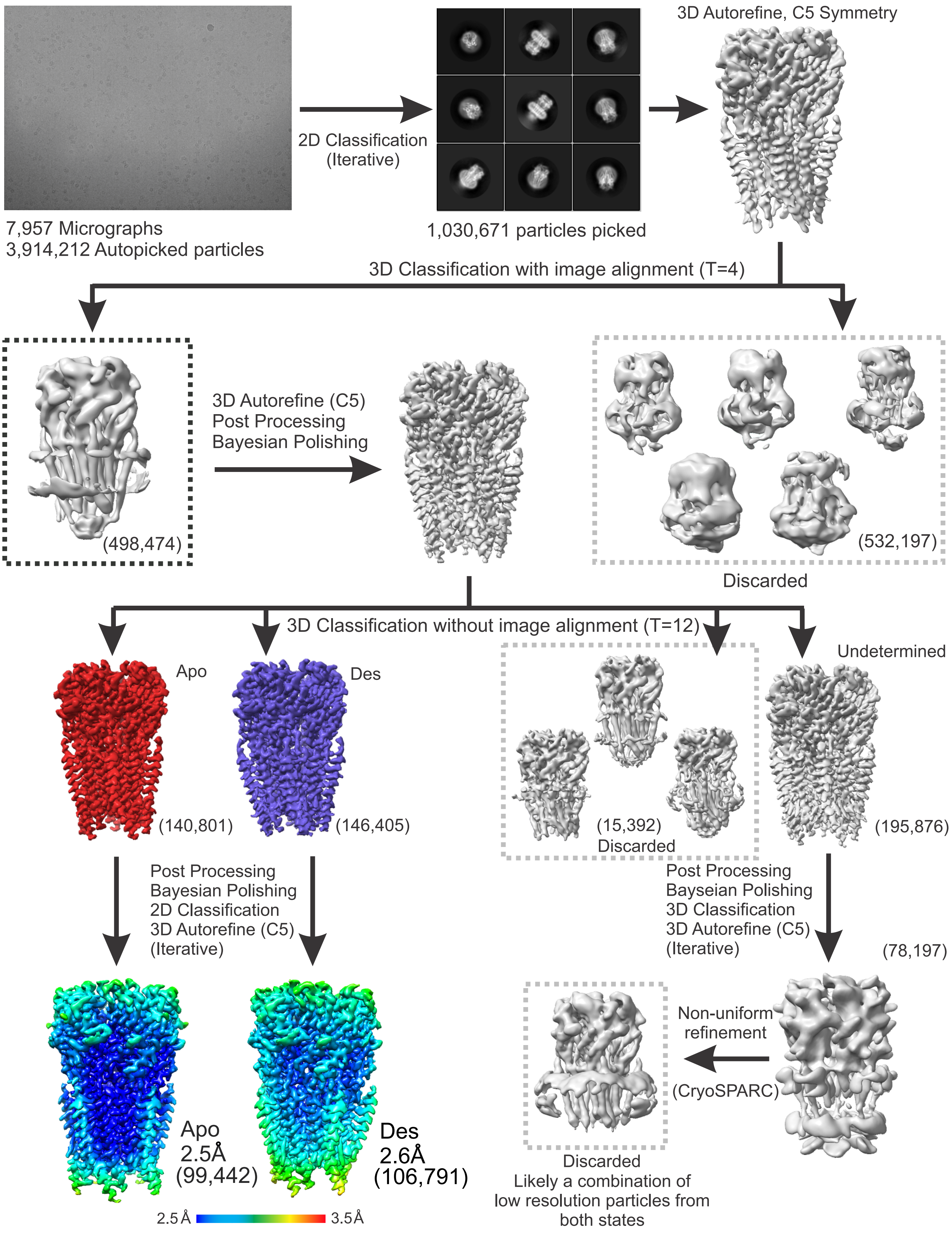

7,957 Micrographs
3,914,212 Autopicked particles
2D Classification
(Iterative)
1,030,671 particles picked
3D Autorefine, C5 Symmetry
3D Classification with image alignment (T=4)
(498,474)
3D Autorefine (C5)
Post Processing
Bayesian Polishing
(532,197)
Discarded
3D Classification without image alignment (T=12)
Apo
(140,801)
Des
(146,405)
(15,392)
Discarded
Undetermined
(195,876)
Post Processing
Bayesian Polishing
2D Classification
3D Autorefine (C5)
(Iterative)
Post Processing
Bayseian Polishing
3D Classification
3D Autorefine (C5)
(Iterative)
(78,197)
Non-uniform
refinement
(CryoSPARC)
Apo
2.5Å
(99,442)
Des
2.6Å
(106,791)
2.5 Å
3.5 Å
Discarded
Likely a combination of
low resolution particles from
both states

Figure S2. RELION processing workflow of the hGlyRα3-0.1g dataset.

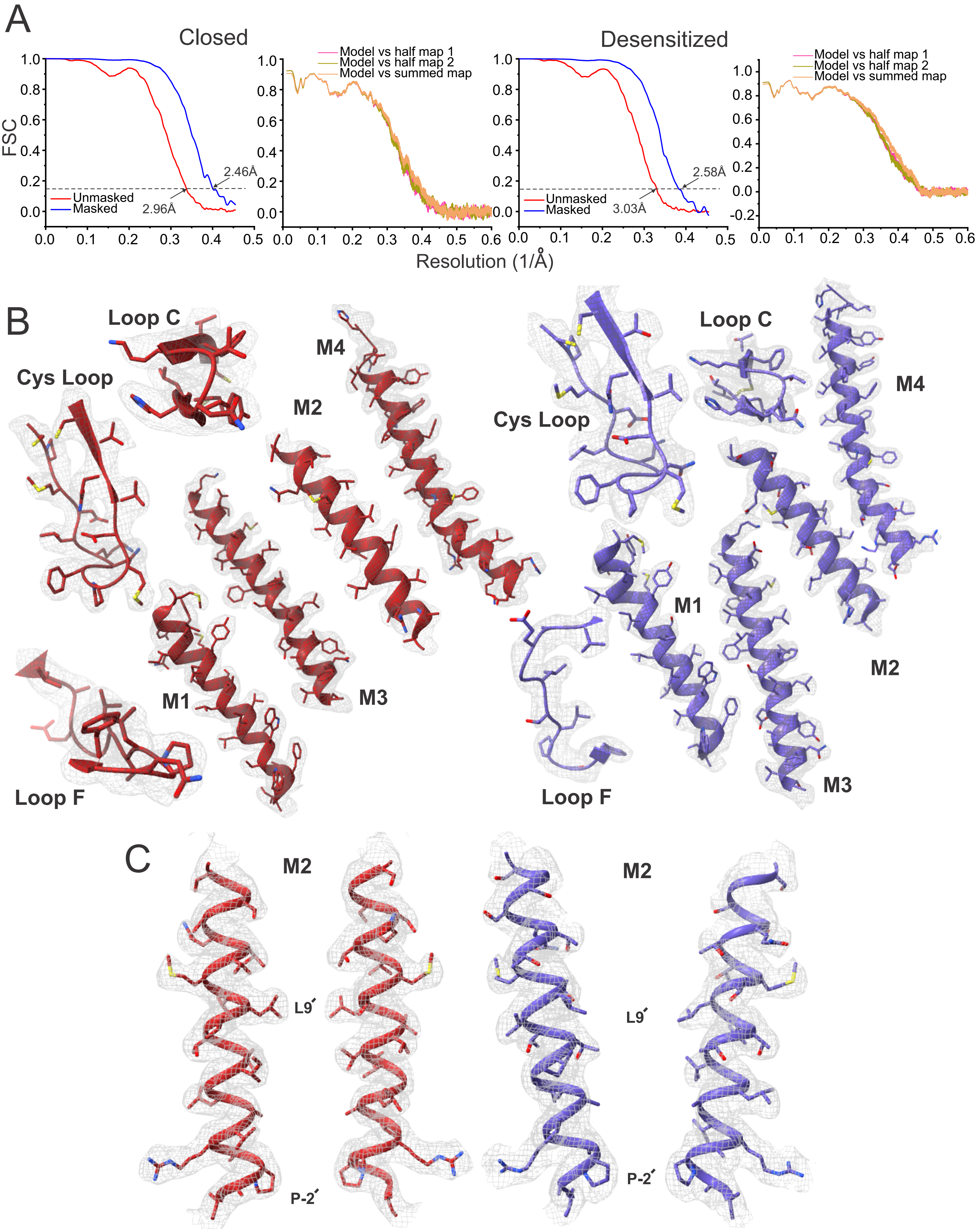

A
Closed
Desensitized
FSC
Unmasked
Masked
2.46Å
2.96Å
2.58Å
3.03Å
Model vs half map 1
Model vs half map 2
Model vs summed map
Resolution (1/Å)
B
Loop C
Cys Loop
M4
M2
M1
M3
Loop F
C
M2
L9´
P-2´

Figure S3. Cryo-EM quality assessment and map/model validation of hGlyRα3-0.1g. (A) Gold standard Fourier shell correlation (FSC) curves of the masked and unmasked maps from RELION 4.0 (*left*). An FSC of 0.143 is indicated by the dashed line. FSC curves of the model versus the summed map, model versus half map 1, and model versus half map 2 calculated using PHENIX mtriage (*right*). (B) Select model regions with the corresponding density for apo (*left*, *red*) and desensitized (*right*, *blue*) states. Threshold levels for both apo and desensitized maps: M2 and loop C = 0.018; all other regions = 0.015. (C) M2 helices and density from two opposing subunits to highlight ion permeation profile.

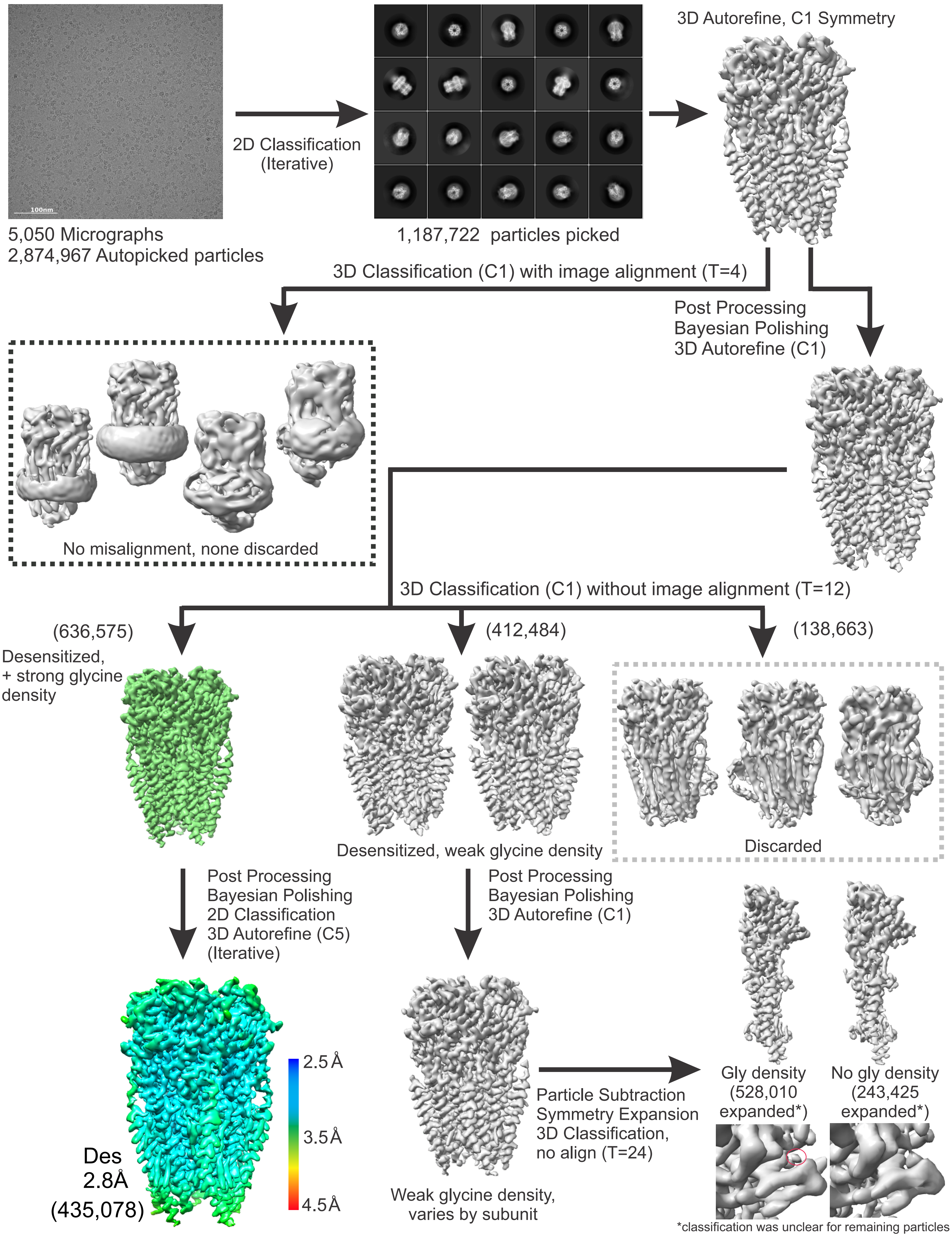
100nm
5,050 Micrographs
2,874,967 Autopicked particles
2D Classification
(Iterative)
1,187,722 particles picked
3D Autorefine, C1 Symmetry
3D Classification (C1) with image alignment (T=4)
Post Processing
Bayesian Polishing
3D Autorefine (C1)
No misalignment, none discarded
3D Classification (C1) without image alignment (T=12)
(636,575)
Desensitized,
+ strong glycine
density
(412,484)
Desensitized, weak glycine density
(138,663)
Discarded
Post Processing
Bayesian Polishing
2D Classification
3D Autorefine (C5)
(Iterative)
Post Processing
Bayesian Polishing
3D Autorefine (C1)
2.5Å
3.5Å
4.5Å
Des
2.8Å
(435,078)
Weak glycine density,
varies by subunit
Particle Subtraction
Symmetry Expansion
3D Classification,
no align (T=24)
Gly density
(528,010
expanded*)
No gly density
(243,425
expanded*)
*classification was unclear for remaining particles

Figure S4. RELION processing workflow of the hGlyRα3-1g dataset.

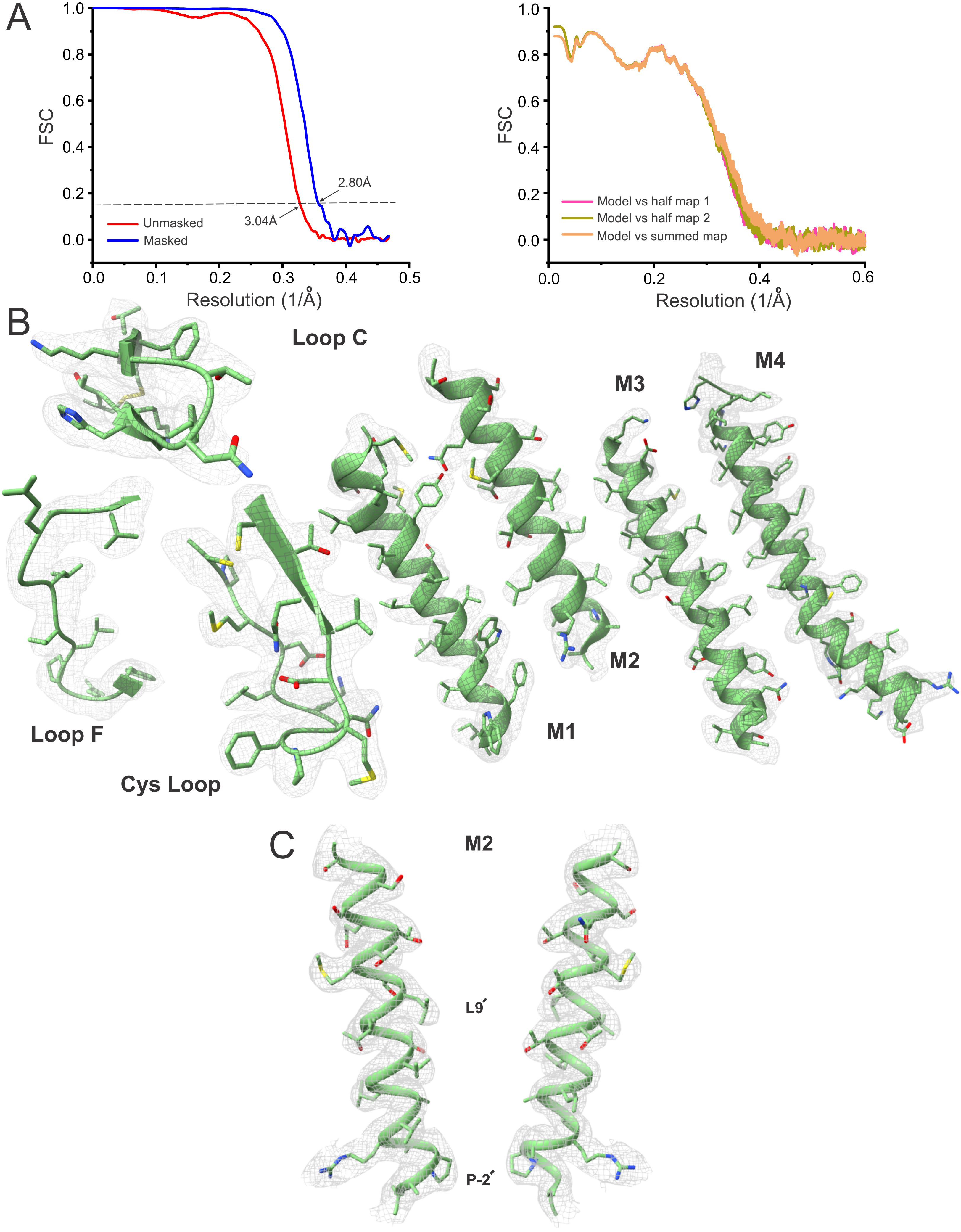
A
FSC
Resolution (1/Å)
2.80Å
3.04Å
Unmasked
Masked
Model vs half map 1
Model vs half map 2
Model vs summed map
B
Loop C
M3
M4
M2
M1
Loop F
Cys Loop
C
M2
L9´
P-2´

Figure S5. Cryo-EM quality assessment and map/model validation of hGlyRα3-1g. (A) Gold standard Fourier shell correlation (FSC) curves of the masked and unmasked maps from RELION 4.0 (*left*). An FSC of 0.143 is indicated by the dashed line. Model/map FSC curves calculated using PHENIX mtriage (*right*). (B) Select model regions with the corresponding density. Threshold levels for the map: M2 and loop C = 0.018; all other regions = 0.015. (C) M2 helices and density from two opposing subunits to highlight ion permeation profile.

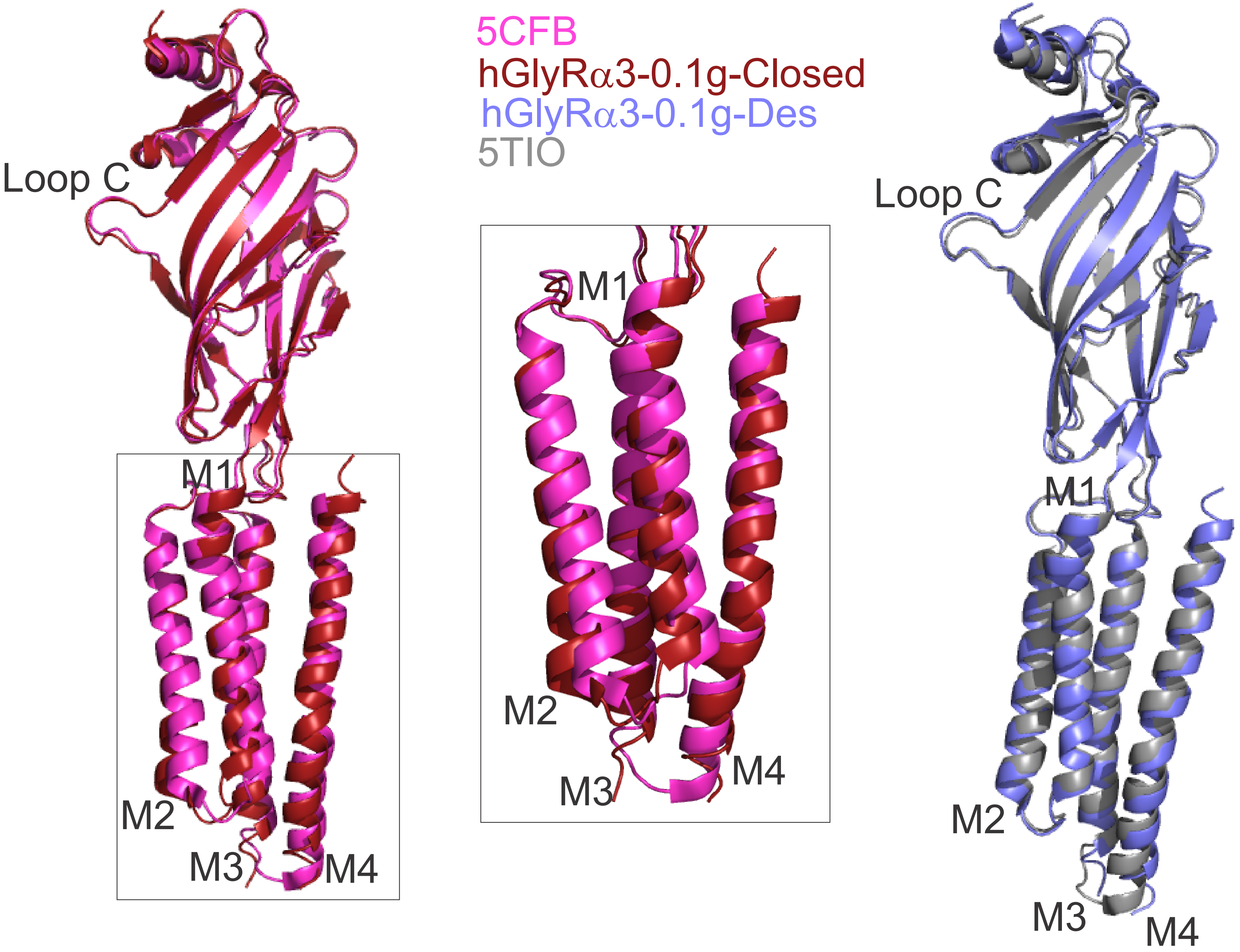

5CFB
hGlyRα3-0.1g-Closed
hGlyRα3-0.1g-Des
5TIO
Loop C
M1
M2
M3
M4
M1
M2
M3
M4
Loop C
M1
M2
M3
M4

Figure S6. A comparison of hGlyRα3-0.1g states with previously solved crystal structures. A single subunit of the hGlyRα3-0.1g-Closed state is aligned with an antagonist-bound closed state (5CFB, pink). The hGlyRα3-0.1g-Des state is aligned with a PAM-bound desensitized state (5TIO, grey). Inset shows hGlyRα3-0.1g-Closed/5CFB alignment slightly tilted with a close-up on the transmembrane domain.

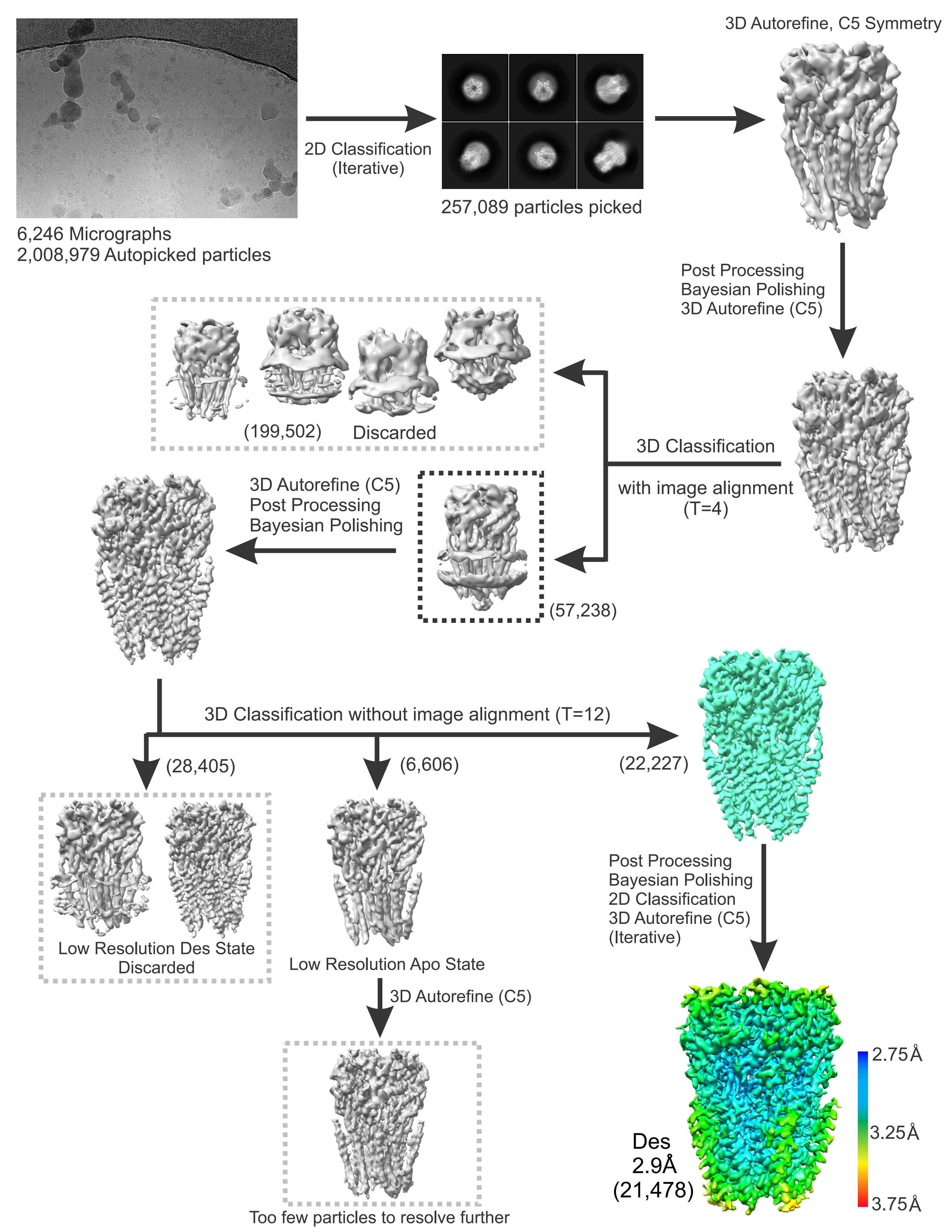
6,246 Micrographs
2,008,979 Autopicked particles
2D Classification
(Iterative)
257,089 particles picked
3D Autorefine, C5 Symmetry
Post Processing
Bayesian Polishing
3D Autorefine (C5)
(199,502)
Discarded
3D Classification
with image alignment
(T=4)
3D Autorefine (C5)
Post Processing
Bayesian Polishing
(57,238)
3D Classification without image alignment (T=12)
(28,405)
(6,606)
(22,227)
Low Resolution Des State
Discarded
Low Resolution Apo State
Post Processing
Bayesian Polishing
2D Classification
3D Autorefine (C5)
(Iterative)
3D Autorefine (C5)
Too few particles to resolve further
Des
2.9Å
(21,478)
2.75Å
3.25Å
3.75Å

Figure S7. RELION processing workflow of the hGlyRα3-1Zn dataset.

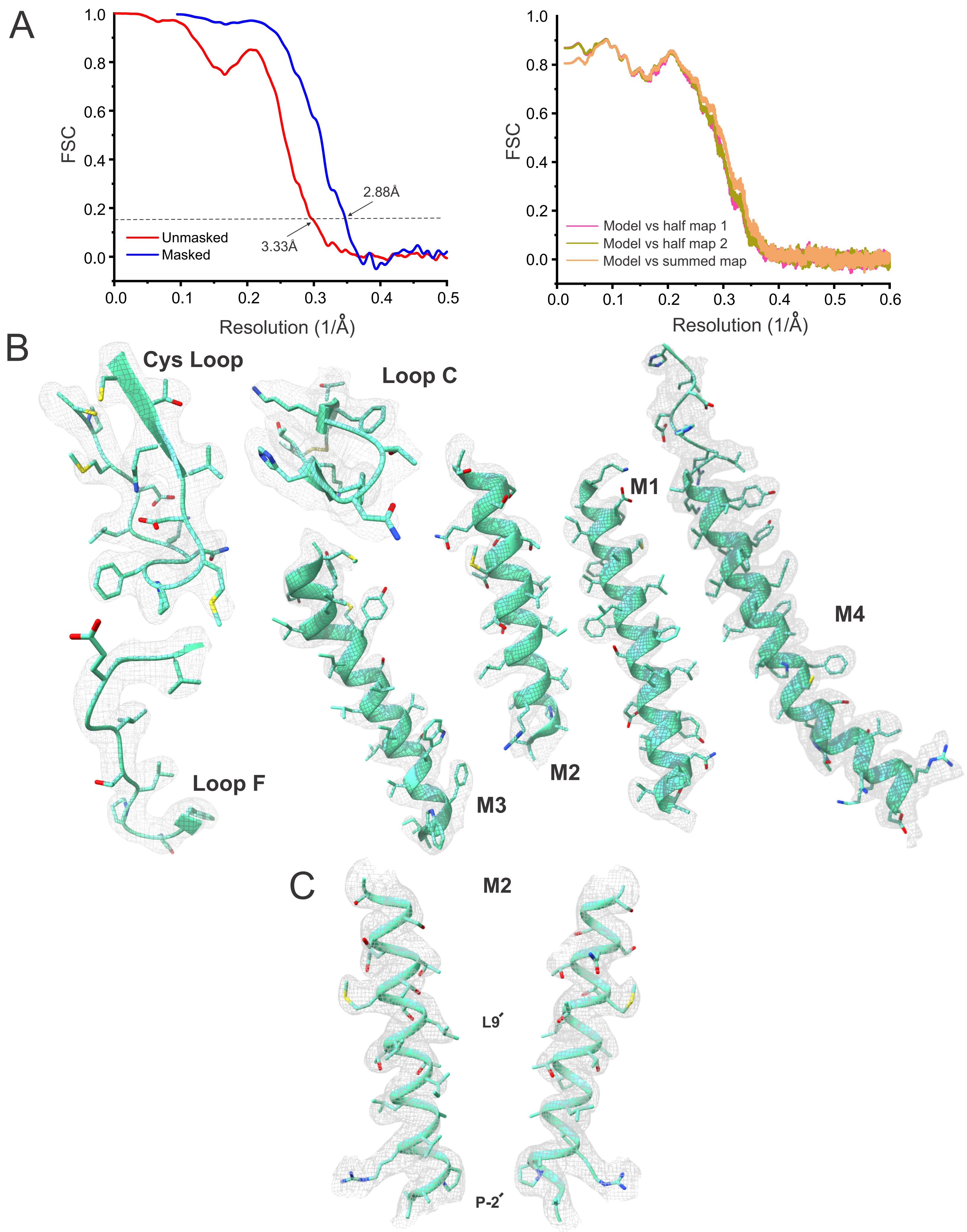
A
1.0
0.8
0.6
0.4
0.2
0.0
FSC
0.0
0.1
0.2
0.3
0.4
0.5
Resolution (1/Å)
Unmasked
Masked
2.88Å
3.33Å
1.0
0.8
0.6
0.4
0.2
0.0
FSC
0.0
0.1
0.2
0.3
0.4
0.5
0.6
Resolution (1/Å)
Model vs half map 1
Model vs half map 2
Model vs summed map
B
Cys Loop
Loop C
M1
M4
Loop F
M3
M2
C
M2
L9´
P-2´

Figure S8. Cryo-EM quality assessment and map/model validation of hGlyRα3-1Zn. (A) Gold standard Fourier shell correlation (FSC) curves of the masked and unmasked maps from RELION 4.0 (*left*). An FSC of 0.143 is indicated by the dashed line. Model/map FSC curves calculated using PHENIX mtriage (*right*). (B) Select model regions with the corresponding density. Threshold levels for map: M2, M3 = 0.01; M4 = 0.011; loop C = 0.0125; all other regions = 0.015. (C) M2 helices and density from two opposing subunits to highlight ion permeation profile.

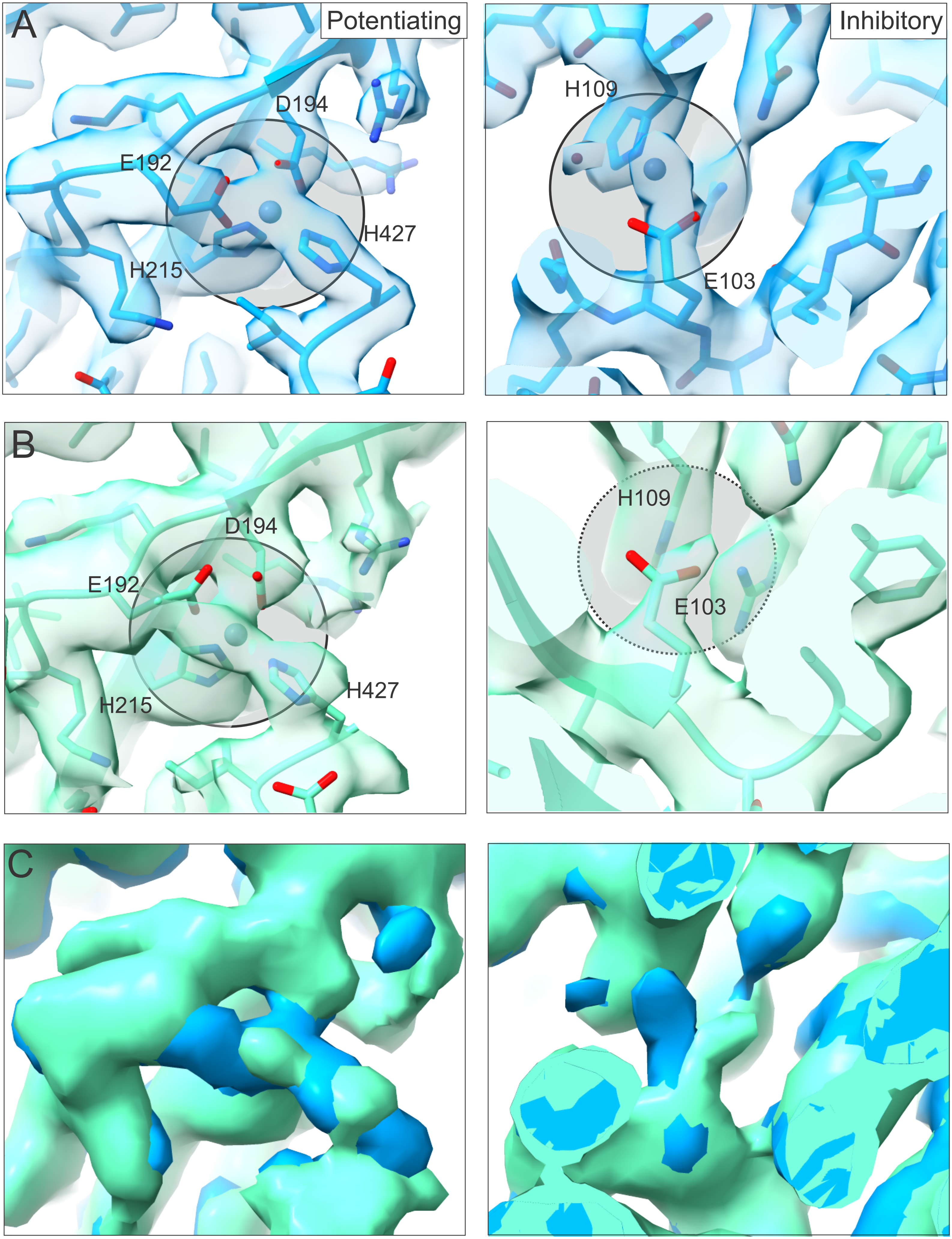
A
Potentiating
D194
E192
H427
H215
Inhibitory
H109
E103
B
D194
E192
H427
H215
H109
E103
C

Figure S9. Comparison of the Zn^{2+} binding sites of hGlyRα3-100Zn-Des and hGlyRα3-1Zn-Des. (A) Potentiating and inhibitory Zn^{2+} binding site for hGlyRα3-100Zn-Des, map and model. (B) Potentiating and inhibitory binding site for hGlyRα3-1Zn-Des, map and model. (C) Overlay of the hGlyRα3-1Zn-Des and hGlyRα3-100Zn-Des maps at the potentiating and inhibitory Zn^{2+} binding sites.

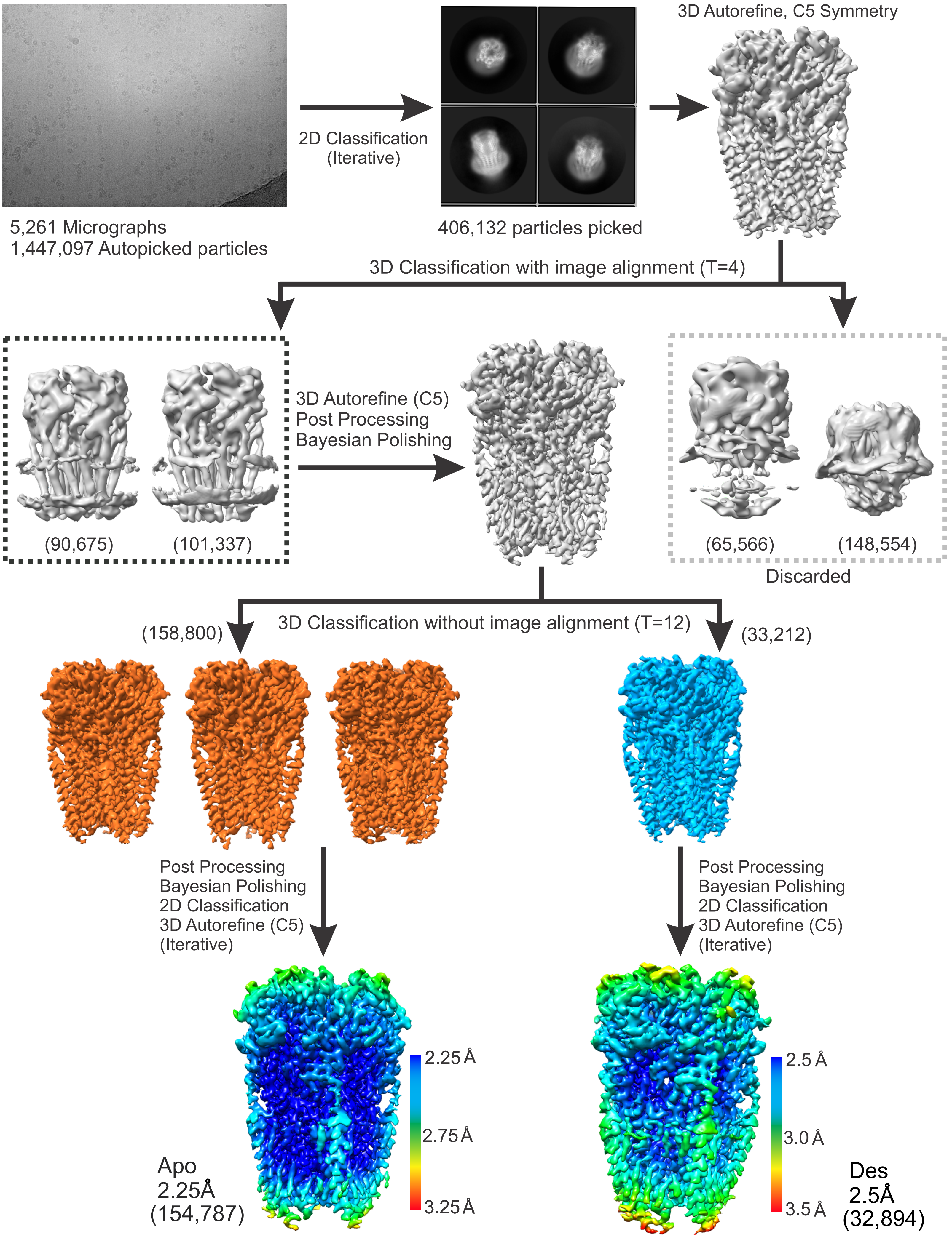

5,261 Micrographs
1,447,097 Autopicked particles
2D Classification
(Iterative)
406,132 particles picked
3D Autorefine, C5 Symmetry
3D Classification with image alignment (T=4)
(90,675)
(101,337)
3D Autorefine (C5)
Post Processing
Bayesian Polishing
(65,566)
(148,554)
Discarded
3D Classification without image alignment (T=12)
(158,800)
(33,212)
Post Processing
Bayesian Polishing
2D Classification
3D Autorefine (C5)
(Iterative)
Post Processing
Bayesian Polishing
2D Classification
3D Autorefine (C5)
(Iterative)
2.25 Å
2.75 Å
3.25 Å
Apo
2.25Å
(154,787)
2.5 Å
3.0 Å
3.5 Å
Des
2.5Å
(32,894)

Figure S10. RELION processing workflow of the hGlyRα3-100Zn dataset.

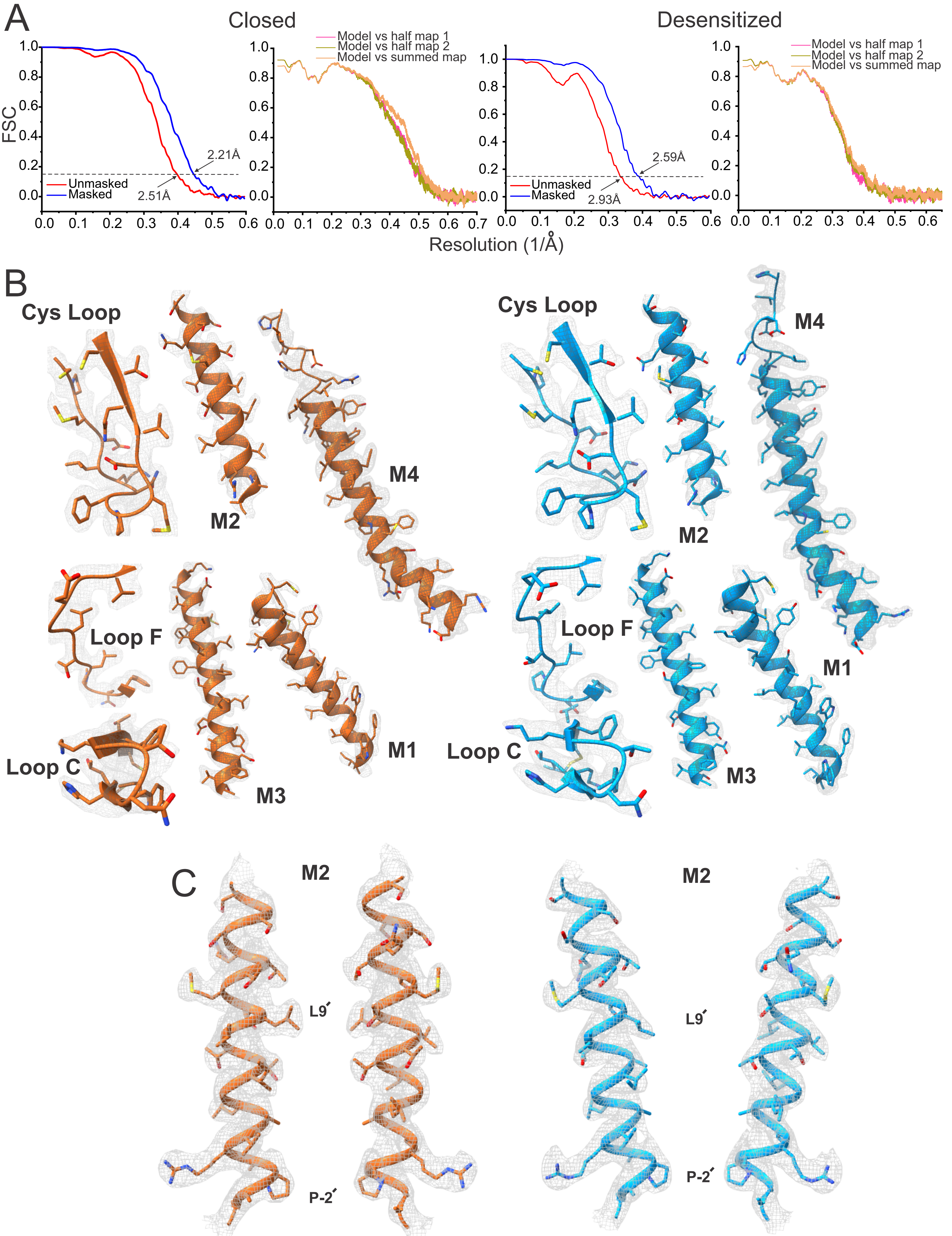

A
Closed
Desensitized
FSC
Unmasked
Masked
2.21Å
2.51Å
Model vs half map 1
Model vs half map 2
Model vs summed map
2.59Å
2.93Å
Resolution (1/Å)
B
Cys Loop
M2
M4
Loop F
Loop C
M3
M1
C
M2
L9′
P-2′

Figure S11. Cryo-EM quality assessment and map/model validation of hGlyRα3-100Zn. (A) Gold standard Fourier shell correlation (FSC) curves of the masked and unmasked maps from RELION 4.0 (*left*). An FSC of 0.143 is indicated by the dashed line. Model/map FSC curves calculated using PHENIX mtriage (*right*). (B) Select model regions with the corresponding density for apo (*left*, *orange*) and desensitized (*right*, *cyan*) states. Threshold levels for both apo and desensitized maps: M1-3 and loop C = 0.018; M4 = 0.0113; all other regions = 0.015. (C) M2 helices and density from two opposing subunits to highlight ion permeation profile.

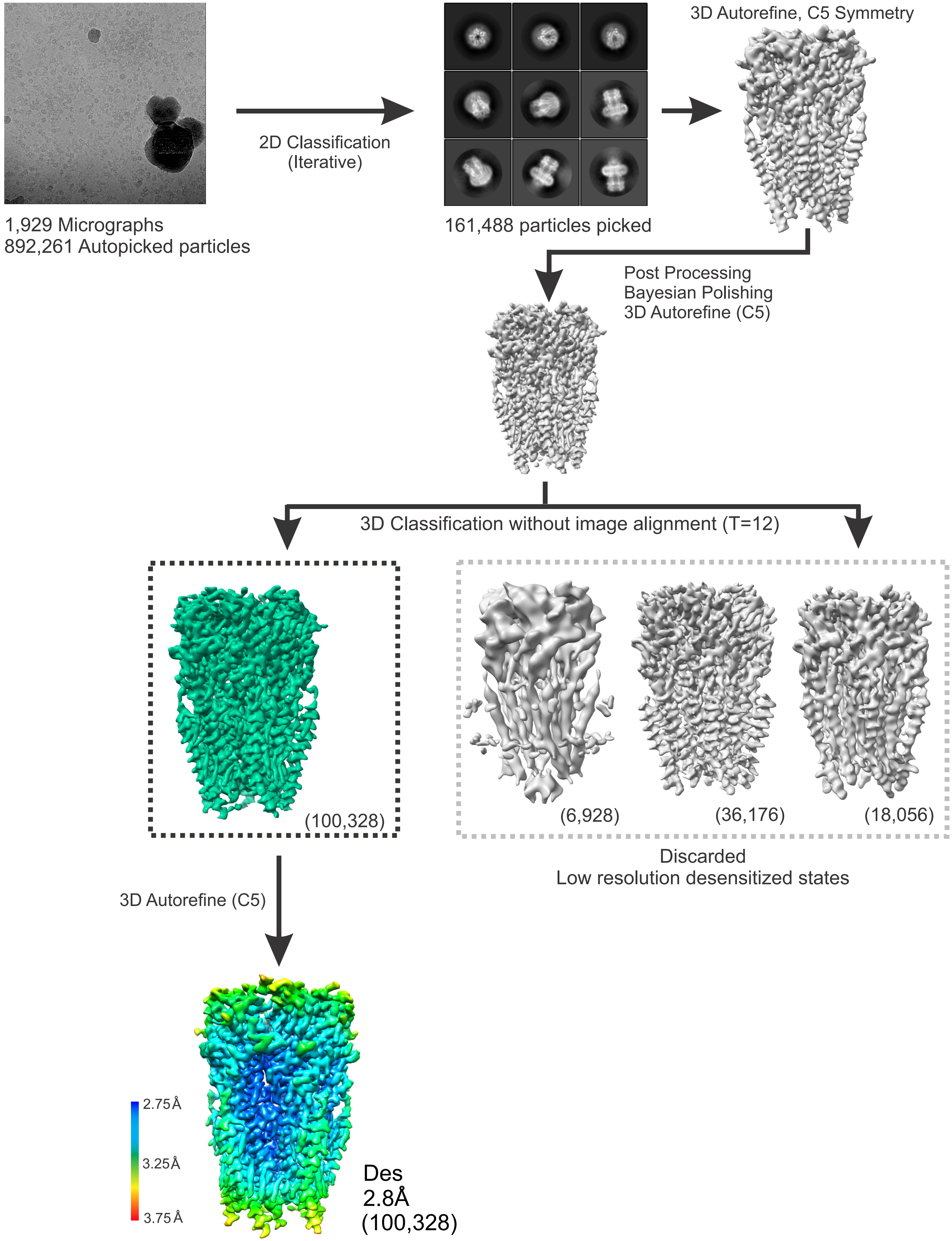

1,929 Micrographs
892,261 Autopicked particles
2D Classification
(Iterative)
161,488 particles picked
3D Autorefine, C5 Symmetry
Post Processing
Bayesian Polishing
3D Autorefine (C5)
3D Classification without image alignment (T=12)
(100,328)
(6,928)
(36,176)
(18,056)
Discarded
Low resolution desensitized states
3D Autorefine (C5)
2.75Å
3.25Å
3.75Å
Des
2.8Å
(100,328)

Figure S12. RELION processing workflow of the 1g-pH6.4 dataset.

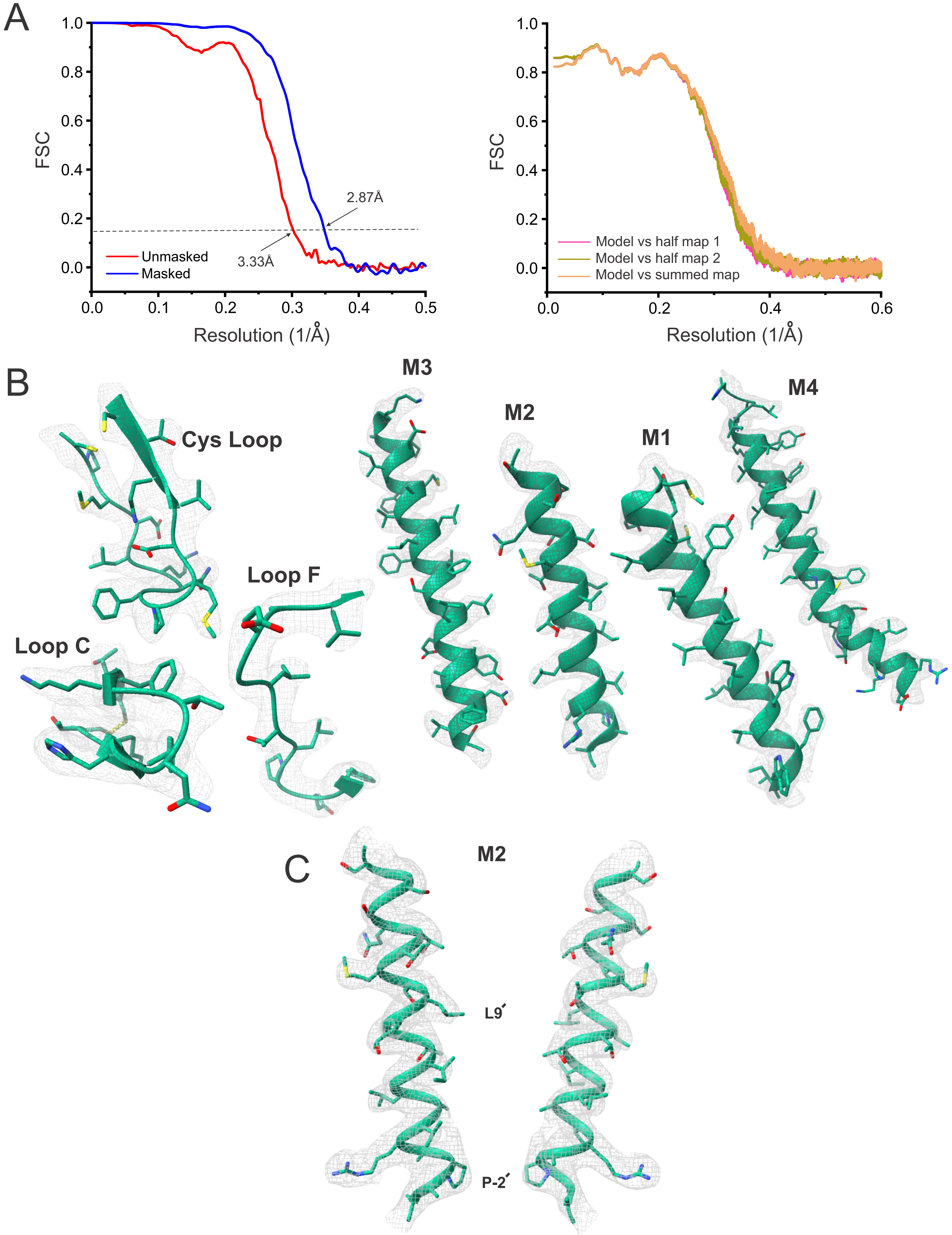
A
1.0
0.8
0.6
0.4
0.2
0.0
FSC
Resolution (1/Å)
0.0
0.1
0.2
0.3
0.4
0.5
2.87Å
3.33Å
Unmasked
Masked
0.6
Model vs half map 1
Model vs half map 2
Model vs summed map
B
Cys Loop
Loop C
Loop F
M3
M2
M1
M4
C
M2
L9´
P-2´

Figure S13. Cryo-EM quality assessment and map/model validation of hGlyRα3-1g-pH6.4. (A) Gold standard Fourier shell correlation (FSC) curves of the masked and unmasked maps from RELION 4.0 (*left*). An FSC of 0.143 is indicated by the dashed line. Model/map FSC curves calculated using PHENIX mtriage (*right*). (B) Select model regions with the corresponding density. Threshold levels for the map: M4 = 0.003; loop C = 0.004; all other regions = 0.0036. (C) M2 helices and density from two opposing subunits to highlight ion permeation profile.

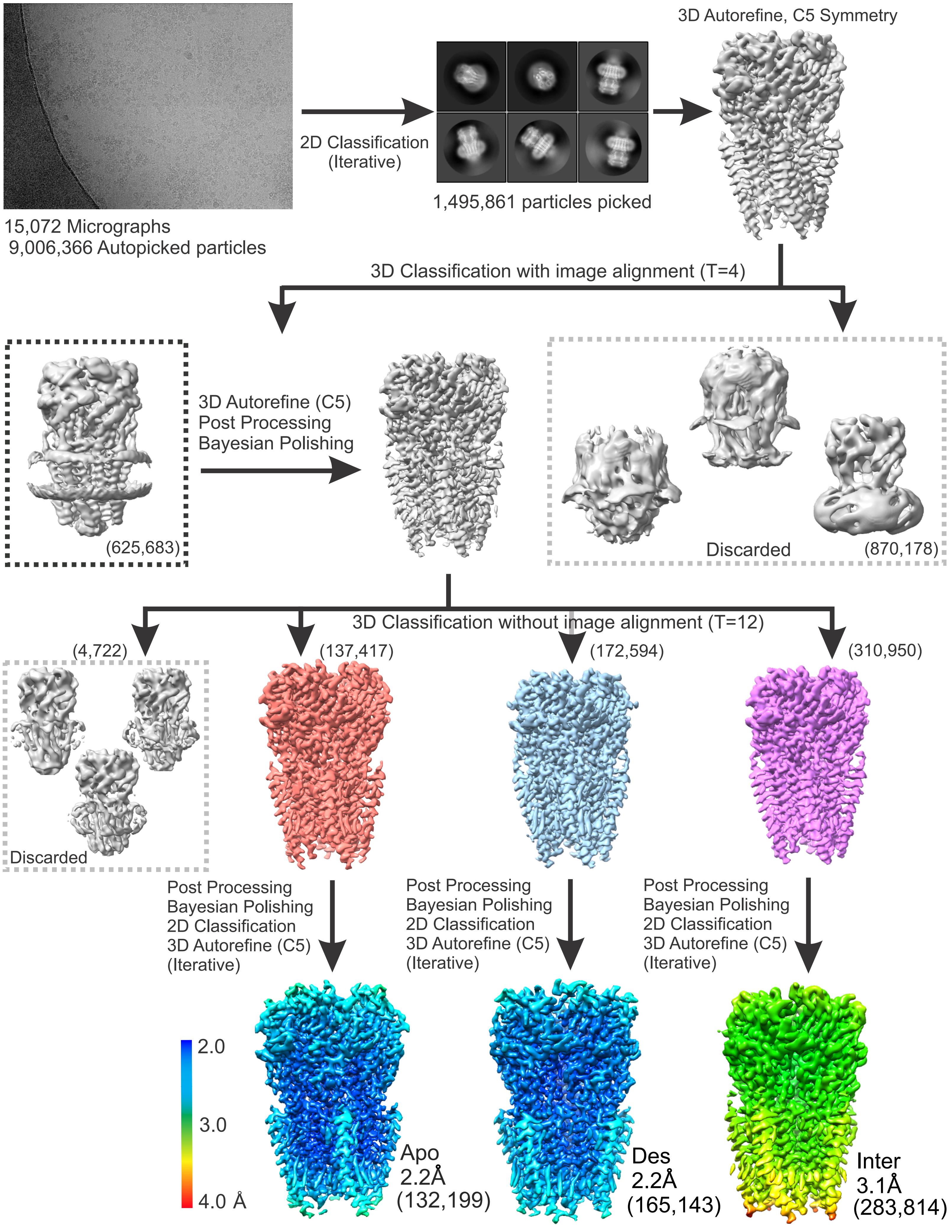
15,072 Micrographs
9,006,366 Autopicked particles
2D Classification
(Iterative)
1,495,861 particles picked
3D Autorefine, C5 Symmetry
3D Classification with image alignment (T=4)
3D Autorefine (C5)
Post Processing
Bayesian Polishing
(625,683)
Discarded
(870,178)
3D Classification without image alignment (T=12)
(4,722)
(137,417)
(172,594)
(310,950)
Discarded
Post Processing
Bayesian Polishing
2D Classification
3D Autorefine (C5)
(Iterative)
Post Processing
Bayesian Polishing
2D Classification
3D Autorefine (C5)
(Iterative)
Post Processing
Bayesian Polishing
2D Classification
3D Autorefine (C5)
(Iterative)
2.0
3.0
4.0 Å
Apo
2.2Å
(132,199)
Des
2.2Å
(165,143)
Inter
3.1Å
(283,814)

Figure S14. RELION processing workflow of the hGlyRα3-0.1g-pH6.4 dataset

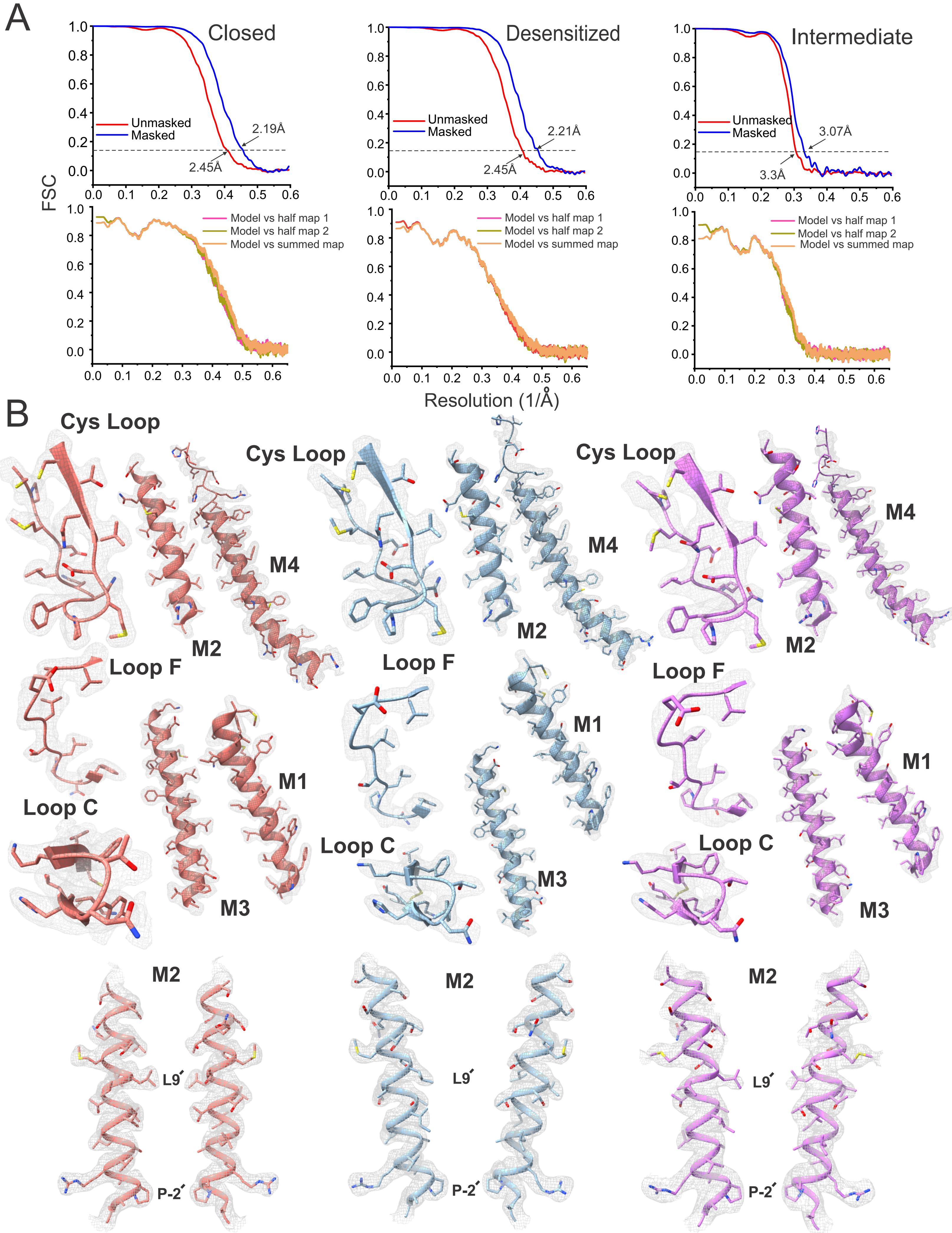
A
Closed
Desensitized
Intermediate
Unmasked
Masked
2.19Å
2.45Å
2.21Å
2.45Å
3.07Å
3.3Å
FSC
Model vs half map 1
Model vs half map 2
Model vs summed map
Resolution (1/Å)
B
Cys Loop
M2
M4
Loop F
M1
M3
Loop C
L9′
P-2′

Figure S15. Cryo-EM quality assessment and map/model validation of hGlyRα3-0.1g-pH6.4. (A) Gold standard Fourier shell correlation (FSC) curves of the masked and unmasked maps from RELION 4.0 (*top*). An FSC of 0.143 is indicated by the dashed line. Model/map FSC curves calculated using PHENIX mtriage (*bottom*). (B) Select model regions with the corresponding density for closed (*left*, *salmon*), desensitized (*middle*, *blue-gray*), and intermediate (*right*, *light purple*) states. Threshold levels for closed map: M1 = 0.02; M2 = 0.018; M4 = 0.0101; all other regions = 0.015. Threshold levels for desensitized map: M1 = 0.02; M2 & M3 = 0.018; M4 = 0.01; loop C = 0.0125; loop F = 0.015; cys loop = 0.018. Threshold levels for intermediate map: M1 = 0.02; M2 = 0.018; M4 = 0.008; loop C = 0.0125; all other regions = 0.015. (C) M2 helices and density from two opposing subunits to highlight ion permeation profile.

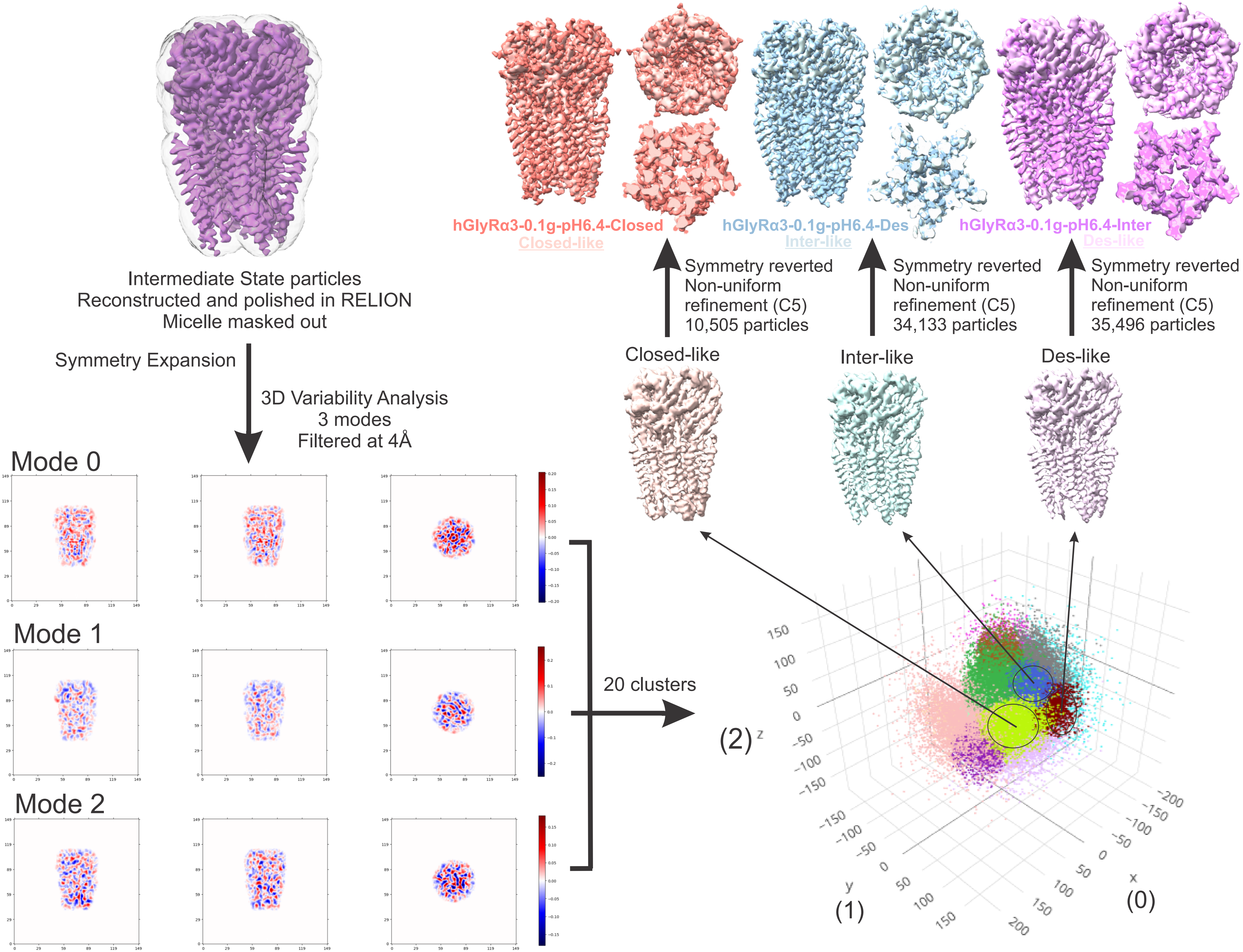

hGlyRα3-0.1g-pH6.4-Closed
Closed-like
hGlyRα3-0.1g-pH6.4-Des
Inter-like
hGlyRα3-0.1g-pH6.4-Inter
Des-like
Intermediate State particles
Reconstructed and polished in RELION
Micelle masked out
Symmetry Expansion
3D Variability Analysis
3 modes
Filtered at 4Å
Symmetry reverted
Non-uniform
refinement (C5)
10,505 particles
Symmetry reverted
Non-uniform
refinement (C5)
34,133 particles
Symmetry reverted
Non-uniform
refinement (C5)
35,496 particles
Closed-like
Inter-like
Des-like
Mode 0
Mode 1
Mode 2
20 clusters
(2) z
y
(1)
x
(0)

Figure S16. CryoSPARC workflow for 3D variability analysis of hGlyRα3-0.1g-pH6.4-Inter. Upper left shows the intermediate state refinement created with RELION and the mask applied for the 3DVA job in CryoSPARC. Three modes of movement were analyzed and split into 20 clusters. From those clusters, a representative was selected that resembled the closed, intermediate, and desensitized state. The unexpanded particles from those clusters were subjected to non-uniform refinement. Upper right shows the alignment of the cluster maps (lighter colors) with the hGlyRα3-0.1g-pH6.4-Closed, Inter, and Des states (darker colors). The resolution of the cluster map refinements was ~3.3 Å for des-like, ~3.3 Å for inter-like, and ~3.4 Å for closed-like.

Data S1. Sequence of plasmids encoding human GlyRα3 for expression in *Xenopus laevis* oocytes.

pCS2-α3:
GCTGCGCAACTGTTGGGAAGGGCGATCGGTGCGGGCCTCTTCGCTATTACGCCAGTC
GACCGCCAATTCAATATGGCGTATATGGACTCATGCCAATTCAATATGGTGGATCTG
GACCTGTGCCAATTCAATATGGCGTATATGGACTCGTGCCAATTCAATATGGTGGAT
CTGGACCCCAGCCAATTCAATATGGCGGACTTGGCACCATGCCAATTCAATATGGCG
GACCTGGCACTGTGCCAACTGGGGAGGGGTCTACTTGGCACGGTGCCAAGTTTGAG
GAGGGGTCTTGGCCCTGTGCCAAGTCCGCCATATTGAATTGGCATGGTGCCAATAAT
GGCGGCCATATTGGCTATATGCCAGGATCAATATATAGGCAATATCCAATATGGCCC
TATGCCAATATGGCTATTGGCCAGGTTCAATACTATGTATTGGCCCTATGCCATATA
GTATTCCATATATGGGTTTTCCTATTGACGTAGATAGCCCCTCCCAATGGGCGGTCCC
ATATACCATATATGGGGCTTCCTAATACCGCCCATAGCCACTCCCCCATTGACGTCA
ATGGTCTCTATATATGGTCTTTCCTATTGACGTCATATGGGCGGTCCTATTGACGTAT
ATGGCGCCTCCCCCATTGACGTCAATTACGGTAAATGGCCCGCCTGGCTCAATGCCC
ATTGACGTCAATAGGACCACCCACCATTGACGTCAATGGGATGGCTCATTGCCCATT
CATATCCGTTCTCACGCCCCCTATTGACGTCAATGACGGTAAATGGCCCACTTGGCA
GTACATCAATATCTATTAATAGTAACTTGGCAAGTACATTACTATTGGAAGTACGCC
AGGGTACATTGGCAGTACTCCCATTGACGTCAATGGCGGTAAATGGCCCGCGATGG
CTGCCAAGTACATCCCCATTGACGTCAATGGGGAGGGGCAATGACGCAAATGGGCG
TTCCATTGACGTAAATGGGCGGTAGGCGTGCCTAATGGGAGGTCTATATAAGCAATG
CTCGTTTAGGGAACCGCCATTCTGCCTGGGGACGTCGGAGCAAGCTTGATTTAGGTG
ACACTATAGAATACAAGCTACTTGTTCTTTTTGCAGGATCCACTAGTGGCGCGCCAT
GGCACATGTAAGACACTTCCGTACTCTAGTGTCTGGGTTCTATTTCTGGGAGGCCGC
TCTATTGCTCAGCCTAGTCGCCACAAAGGAAACAGACAGCGCCCGCTCCAGATCCG
CCCCGATGAGTCCCAGTGATTTCTTAGATAAATTGATGGGCAGAACCTCTGGGTATG
ACGCGCGCATTCGCCCGAACTTTAAGGGACCCCCAGTAAACGTTACTTGTAATATTT
TTATCAATAGCTTTGGTTCAATTGCAGAAACCACTATGGATTACCGGGTGAATATTT
TCTTGCGGCAAAAGTGGAACGACCCTAGACTCGCGTATTCGGAATACCCCGATGACT
CCCTGGATTTAGATCCTTCTATGCTTGATTCAATTTGGAAGCCCGATTTATTTTTCGC
GAACGAGAAAGGTGCTAATTTTCATGAAGTTACAACCGACAACAAACTACTTCGAA
TCTTTAAAAACGGAAATGTTTTATACTCGATACGGCTTACATTAACTTTGAGCTGCCC
CATGGATCTAAAAAATTTCCCAATGGACGTTCAGACGTGTATTATGCAGCTTGAGTC
TTTCGGCTACACTATGAACGATCTTATATTCGAGTGGCAGGATGAAGCTCCAGTACA
GGTCGCCGAGGGCTTGACGCTCCCACAGTTTCTGTTAAAAGAGGAGAAGGATTTGC
GTTACTGTACCAAGCACTATAACACTGGCAAATTTACCTGCATAGAAGTCCGCTTCC
ACCTGGAGCGCCAAATGGGTTATTATTTGATTCAAATGTACATCCCATCGCTACTGA
TAGTTATATTATCATGGGTCTCGTTTTGGATTAATATGGACGCAGCTCCGGCTCGGGT
GGCTCTCGGTATCACTACGGTGTTAACCATGACGACACAATCTTCAGGATCCAGGGC
CTCACTGCCTAAGGTTAGCTACGTGAAAGCAATAGACATATGGATGGCAGTATGCTT

ACTTTTTGTGTTTAGTGCGCTCCTTGAGTACGCCGCTGTGAATTTTGTCAGTCGACAG
CACAAAGAGTTGCTACGATTCCGACGTAAGAGGAAAAACAAGACAGAAGCATTCGC
ACTCGAAAAATTCTATCGTTTCTCCGACATGGACGATGAAGTAAGGGAAAGTAGGTT
TAGTTTTACGGCATATGGTATGGGGCCTTGCCTCCAGGCGAAAGACGGAATGACGCC
GAAGGGGCCTAATCACCCGGTACAAGTAATGCCTAAGTCGCCCGACGAGATGCGAA
AGGTCTTCATAGACCGGGCGAAGAAAATTGATACGATCTCAAGAGCTTGTTTCCCAT
TGGCGTTCCTAATCTTTAACATATTTTATTGGGTTATCTACAAGATCCTCCGTCATGA
AGATATCCATCAACAACAGGACTGAGGTACCTCTAGAACTATAGTGAGTCGTATTAC
GTAGATCCAGACATGATAAGATACATTGATGAGTTTGGACAAACCACAACTAGAAT
GCAGTGAAAAAAATGCTTTATTTGTGAAATTTGTGATGCTATTGCTTTATTTGTAACC
ATTATAAGCTGCAATAAACAAGTTAACAACAACAATTGCATTCATTTTATGTTTCAG
GTTCAGGGGGAGGTGTGGGAGGTTTTTTAATTCGCGGCCGCGGCGCCAATGCATTGG
GCCCGGTACCCAGCTTTTGTTCCCTTTAGTGAGGGTTAATTGCGCGCTTGGCGTAATC
ATGGTCATAGCTGTTTCCTGTGTGAAATTGTTATCCGCTCACAATTCCACACAACATA
CGAGCCGGGAGCATAAAGTGTAAAGCCTGGGGTGCCTAATGAGTGAGCTAACTCAC
ATTAATTGCGTTGCGCTCACTGCCCGCTTTCCAGTCGGGAAACCTGTCGTGCCAGCT
GCATTAATGAATCGGCCAACGCGCGGGGAGAGGCGGTTTGCGTATTGGGCGCTCTTC
CGCTTCCTCGCTCACTGACTCGCTGCGCTCGGTCGTTCGGCTGCGGCGAGCGGTATC
AGCTCACTCAAAGGCGGTAATACGGTTATCCACAGAATCAGGGGATAACGCAGGAA
AGAACATGTGAGCAAAAGGCCAGCAAAAGGCCAGGAACCGTAAAAAGGCCGCGTT
GCTGGCGTTTTTCCATAGGCTCCGCCCCCCTGACGAGCATCACAAAAATCGACGCTC
AAGTCAGAGGTGGCGAAACCCGACAGGACTATAAAGATACCAGGCGTTTCCCCCTG
GAAGCTCCCTCGTGCGCTCTCCTGTTCCGACCCTGCCGCTTACCGGATACCTGTCCGC
CTTTCTCCCTTCGGGAAGCGTGGCGCTTTCTCATAGCTCACGCTGTAGGTATCTCAGT
TCGGTGTAGGTCGTTCGCTCCAAGCTGGGCTGTGTGCACGAACCCCCCGTTCAGCCC
GACCGCTGCGCCTTATCCGGTAACTATCGTCTTGAGTCCAACCCGGTAAGACACGAC
TTATCGCCACTGGCAGCAGCCACTGGTAACAGGATTAGCAGAGCGAGGTATGTAGG
CGGTGCTACAGAGTTCTTGAAGTGGTGGCCTAACTACGGCTACACTAGAAGAACAG
TATTTGGTATCTGCGCTCTGCTGAAGCCAGTTACCTTCGGAAAAAGAGTTGGTAGCT
CTTGATCCGGCAAACAAACCACCGCTGGTAGCGGTGGTTTTTTTGTTTGCAAGCAGC
AGATTACGCGCAGAAAAAAAGGATCTCAAGAAGATCCTTTGATCTTTTCTACGGGGT
CTGACGCTCAGTGGAACGAAAACTCACGTTAAGGGATTTTGGTCATGAGATTATCAA
AAAGGATCTTCACCTAGATCCTTTTAAATTAAAAATGAAGTTTTAAATCAATCTAAA
GTATATATGAGTAAACTTGGTCTGACAGTTACCAATGCTTAATCAGTGAGGCACCTA
TCTCAGCGATCTGTCTATTTCGTTCATCCATAGTTGCCTGACTCCCCGTCGTGTAGAT
AACTACGATACGGGAGGGCTTACCATCTGGCCCCAGTGCTGCAATGATACCGCGAG
ACCCACGCTCACCGGCTCCAGATTTATCAGCAATAAACCAGCCAGCCGGAAGGGCC
GAGCGCAGAAGTGGTCCTGCAACTTTATCCGCCTCCATCCAGTCTATTAATTGTTGC
CGGGAAGCTAGAGTAAGTAGTTCGCCAGTTAATAGTTTGCGCAACGTTGTTGCCATT
GCTACAGGCATCGTGGTGTCACGCTCGTCGTTTGGTATGGCTTCATTCAGCTCCGGTT

CCCAACGATCAAGGCGAGTTACATGATCCCCCATGTTGTGCAAAAAAGCGGTTAGCT
CCTTCGGTCCTCCGATCGTTGTCAGAAGTAAGTTGGCCGCAGTGTTATCACTCATGG
TTATGGCAGCACTGCATAATTCTCTTACTGTCATGCCATCCGTAAGATGCTTTTCTGT
GACTGGTGAGTACTCAACCAAGTCATTCTGAGAATAGTGTATGCGGCGACCGAGTTG
CTCTTGCCCGGCGTCAATACGGGATAATACCGCGCCACATAGCAGAACTTTAAAAGT
GCTCATCATTGGAAAACGTTCTTCGGGGCGAAAACTCTCAAGGATCTTACCGCTGTT
GAGATCCAGTTCGATGTAACCCACTCGTGCACCCAACTGATCTTCAGCATCTTTTACT
TTCACCAGCGTTTCTGGGTGAGCAAAAACAGGAAGGCAAAATGCCGCAAAAAAGGG
AATAAGGGCGACACGGAAATGTTGAATACTCATACTCTTCCTTTTTCAATATTATTG
AAGCATTTATCAGGGTTATTGTCTCATGAGCGGATACATATTTGAATGTATTTAGAA
AAATAAACAAATAGGGGTTCCGCGCACATTTCCCCGAAAAGTGCCACCTAAATTGT
AAGCGTTAATATTTTGTTAAAATTCGCGTTAAATTTTTGTTAAATCAGCTCATTTTTT
AACCAATAGGCCGAAATCGGCAAAATCCCTTATAAATCAAAAGAATAGACCGAGAT
AGGGTTGAGTGTTGTTCCAGTTTGGAACAAGAGTCCACTATTAAAGAACGTGGACTC
CAACGTCAAAGGGCGAAAAACCGTCTATCAGGGCGATGGCCCACTACGTGAACCAT
CACCCTAATCAAGTTTTTTGGGGTCGAGGTGCCGTAAAGCACTAAATCGGAACCCTA
AAGGGAGCCCCCGATTTAGAGCTTGACGGGGAAAGCCGGCGAACGTGGCGAGAAA
GGAAGGGAAGAAAGCGAAAGGAGCGGGCGCTAGGGCGCTGGCAAGTGTAGCGGTC
ACGCTGCGCGTAACCACCACACCCGCCGCGCTTAATGCGCCGCTACAGGGCGCGTC
CCATTCGCCATTCAG

pCS2-α3_E103A:
CGCCATTCTGCCTGGGGACGTCGGAGCAAGCTTGATTTAGGTGACACTATAGAATAC
AAGCTACTTGTTCTTTTTGCAGGATCGGTACCGGGCCCGCCGCCACCATGGCACATG
TAAGACACTTCCGTACTCTAGTGTCTGGGTTCTATTTCTGGGAGGCCGCTCTATTGCT
CAGCCTAGTCGCCACAAAGGAAACAGACAGCGCCCGCTCCAGATCCGCCCCGATGA
GTCCCAGTGATTTCTTAGATAAATTGATGGGCAGAACCTCTGGGTATGACGCGCGCA
TTCGCCCGAACTTTAAGGGACCCCCAGTAAACGTTACTTGTAATATTTTTATCAATA
GCTTTGGTTCAATTGCAGAAACCACTATGGATTACCGGGTGAATATTTTCTTGCGGC
AAAAGTGGAACGACCCTAGACTCGCGTATTCGGAATACCCCGATGACTCCCTGGATT
TAGATCCTTCTATGCTTGATTCAATTTGGAAGCCCGATTTATTTTTCGCGAACGCGAA
AGGTGCTAATTTTCATGAAGTTACAACCGACAACAAACTACTTCGAATCTTTAAAAA
CGGAAATGTTTTATACTCGATACGGCTTACATTAACTTTGAGCTGCCCCATGGATCT
AAAAAATTTCCCAATGGACGTTCAGACGTGTATTATGCAGCTTGAGTCTTTCGGCTA
CACTATGAACGATCTTATATTCGAGTGGCAGGATGAAGCTCCAGTACAGGTCGCCGA
GGGCTTGACGCTCCCACAGTTTCTGTTAAAAGAGGAGAAGGATTTGCGTTACTGTAC
CAAGCACTATAACACTGGCAAATTTACCTGCATAGAAGTCCGCTTCCACCTGGAGCG
CCAAATGGGTTATTATTTGATTCAAATGTACATCCCATCGCTACTGATAGTTATATTA
TCATGGGTCTCGTTTTGGATTAATATGGACGCAGCTCCGGCTCGGGTGGCTCTCGGT
ATCACTACGGTGTTAACCATGACGACACAATCTTCAGGATCCAGGGCCTCACTGCCT
AAGGTTAGCTACGTGAAAGCAATAGACATATGGATGGCAGTATGCTTACTTTTTGTG

TTTAGTGCGCTCCTTGAGTACGCCGCTGTGAATTTTGTCAGTCGACAGCACAAAGAG
TTGCTACGATTCCGACGTAAGAGGAAAAACAAGACAGAAGCATTCGCACTCGAAAA
ATTCTATCGTTTCTCCGACATGGACGATGAAGTAAGGGAAAGTAGGTTTAGTTTTAC
GGCATATGGTATGGGGCCTTGCCTCCAGGCGAAAGACGGAATGACGCCGAAGGGGC
CTAATCACCCGGTACAAGTAATGCCTAAGTCGCCCGACGAGATGCGAAAGGTCTTC
ATAGACCGGGCGAAGAAAATTGATACGATCTCAAGAGCTTGTTTCCCATTGGCGTTC
CTAATCTTTAACATATTTTATTGGGTTATCTACAAGATCCTCCGTCATGAAGATATCC
ATCAACAACAGGACTGAGGTACCTAGCATCGATGGATCCTCGAGCCTCTAGAACTAT
AGTGAGTCGTATTACGTAGATCCAGACATGATAAGATACATTGATGAGTTTGGACAA
ACCACAACTAGAATGCAGTGAAAAAAATGCTTTATTTGTGAAATTTGTGATGCTATT
GCTTTATTTGTAACCATTATAAGCTGCAATAAACAAGTTAACAACAACAATTGCATT
CATTTTATGTTTCAGGTTCAGGGGGAGGTGTGGGAGGTTTTTTAATTCGCGGCCGCG
GCGCCAATGCATTGGGCCCGGTACCCAGCTTTTGTTCCCTTTAGTGAGGGTTAATTG
CGCGCTTGGCGTAATCATGGTCATAGCTGTTTCCTGTGTGAAATTGTTATCCGCTCAC
AATTCCACACAACATACGAGCCGGAAGCATAAAGTGTAAAGCCTGGGGTGCCTAAT
GAGTGAGCTAACTCACATTAATTGCGTTGCGCTCACTGCCCGCTTTCCAGTCGGGAA
ACCTGTCGTGCCAGCTGCATTAATGAATCGGCCAACGCGCGGGGAGAGGCGGTTTG
CGTATTGGGCGCTCTTCCGCTTCCTCGCTCACTGACTCGCTGCGCTCGGTCGTTCGGC
TGCGGCGAGCGGTATCAGCTCACTCAAAGGCGGTAATACGGTTATCCACAGAATCA
GGGGATAACGCAGGAAAGAACATGTGAGCAAAAGGCCAGCAAAAGGCCAGGAACC
GTAAAAAGGCCGCGTTGCTGGCGTTTTTCCATAGGCTCCGCCCCCCTGACGAGCATC
ACAAAAATCGACGCTCAAGTCAGAGGTGGCGAAACCCGACAGGACTATAAAGATAC
CAGGCGTTTCCCCCTGGAAGCTCCCTCGTGCGCTCTCCTGTTCCGACCCTGCCGCTTA
CCGGATACCTGTCCGCCTTTCTCCCTTCGGGAAGCGTGGCGCTTTCTCATAGCTCACG
CTGTAGGTATCTCAGTTCGGTGTAGGTCGTTCGCTCCAAGCTGGGCTGTGTGCACGA
ACCCCCCGTTCAGCCCGACCGCTGCGCCTTATCCGGTAACTATCGTCTTGAGTCCAA
CCCGGTAAGACACGACTTATCGCCACTGGCAGCAGCCACTGGTAACAGGATTAGCA
GAGCGAGGTATGTAGGCGGTGCTACAGAGTTCTTGAAGTGGTGGCCTAACTACGGC
TACACTAGAAGGACAGTATTTGGTATCTGCGCTCTGCTGAAGCCAGTTACCTTCGGA
AAAAGAGTTGGTAGCTCTTGATCCGGCAAACAAACCACCGCTGGTAGCGGTGGTTTT
TTTGTTTGCAAGCAGCAGATTACGCGCAGAAAAAAAGGATCTCAAGAAGATCCTTT
GATCTTTTCTACGGGGTCTGACGCTCAGTGGAACGAAAACTCACGTTAAGGGATTTT
GGTCATGAGATTATCAAAAAGGATCTTCACCTAGATCCTTTTAAATTAAAAATGAAG
TTTTAAATCAATCTAAAGTATATATGAGTAAACTTGGTCTGACAGTTACCAATGCTT
AATCAGTGAGGCACCTATCTCAGCGATCTGTCTATTTCGTTCATCCATAGTTGCCTGA
CTCCCCGTCGTGTAGATAACTACGATACGGGAGGGCTTACCATCTGGCCCCAGTGCT
GCAATGATACCGCGAGACCCACGCTCACCGGCTCCAGATTTATCAGCAATAAACCA
GCCAGCCGGAAGGGCCGAGCGCAGAAGTGGTCCTGCAACTTTATCCGCCTCCATCC
AGTCTATTAATTGTTGCCGGGAAGCTAGAGTAAGTAGTTCGCCAGTTAATAGTTTGC
GCAACGTTGTTGCCATTGCTACAGGCATCGTGGTGTCACGCTCGTCGTTTGGTATGG

CTTCATTCAGCTCCGGTTCCCAACGATCAAGGCGAGTTACATGATCCCCCATGTTGT
GCAAAAAAGCGGTTAGCTCCTTCGGTCCTCCGATCGTTGTCAGAAGTAAGTTGGCCG
CAGTGTTATCACTCATGGTTATGGCAGCACTGCATAATTCTCTTACTGTCATGCCATC
CGTAAGATGCTTTTCTGTGACTGGTGAGTACTCAACCAAGTCATTCTGAGAATAGTG
TATGCGGCGACCGAGTTGCTCTTGCCCGGCGTCAATACGGGATAATACCGCGCCACA
TAGCAGAACTTTAAAAGTGCTCATCATTGGAAAACGTTCTTCGGGGCGAAAACTCTC
AAGGATCTTACCGCTGTTGAGATCCAGTTCGATGTAACCCACTCGTGCACCCAACTG
ATCTTCAGCATCTTTTACTTTCACCAGCGTTTCTGGGTGAGCAAAAACAGGAAGGCA
AAATGCCGCAAAAAAGGGAATAAGGGCGACACGGAAATGTTGAATACTCATACTCT
TCCTTTTTCAATATTATTGAAGCATTTATCAGGGTTATTGTCTCATGAGCGGATACAT
ATTTGAATGTATTTAGAAAAATAAACAAATAGGGGTTCCGCGCACATTTCCCCGAAA
AGTGCCACCTGACGCGCCCTGTAGCGGCGCATTAAGCGCGGCGGGTGTGGTGGTTA
CGCGCAGCGTGACCGCTACACTTGCCAGCGCCCTAGCGCCCGCTCCTTTCGCTTTCTT
CCCTTCCTTTCTCGCCACGTTCGCCGGCTTTCCCCGTCAAGCTCTAAATCGGGGGCTC
CCTTTAGGGTTCCGATTTAGTGCTTTACGGCACCTCGACCCCAAAAAACTTGATTAG
GGTGATGGTTCACGTAGTGGGCCATCGCCCTGATAGACGGTTTTTCGCCCTTTGACG
TTGGAGTCCACGTTCTTTAATAGTGGACTCTTGTTCCAAACTGGAACAACACTCAAC
CCTATCTCGGTCTATTCTTTTGATTTATAAGGGATTTTGCCGATTTCGGCCTATTGGTT
AAAAAATGAGCTGATTTAACAAAAATTTAACGCGAATTTTAACAAAATATTAACGCT
TACAATTTCCATTCGCCATTCAGGCTGCGCAACTGTTGGGAAGGGCGATCGGTGCGG
GCCTCTTCGCTATTACGCCAGTCGACCATAGCCAATTCAATATGGCGTATATGGACT
CATGCCAATTCAATATGGTGGATCTGGACCTGTGCCAATTCAATATGGCGTATATGG
ACTCGTGCCAATTCAATATGGTGGATCTGGACCCCAGCCAATTCAATATGGCGGACT
TGGCACCATGCCAATTCAATATGGCGGACTTGGCACTGTGCCAACTGGGGAGGGGT
CTACTTGGCACGGTGCCAAGTTTGAGGAGGGGTCTTGGCCCTGTGCCAAGTCCGCCA
TATTGAATTGGCATGGTGCCAATAATGGCGGCCATATTGGCTATATGCCAGGATCAA
TATATAGGCAATATCCAATATGGCCCTATGCCAATATGGCTATTGGCCAGGTTCAAT
ACTATGTATTGGCCCTATGCCATATAGTATTCCATATATGGGTTTTCCTATTGACGTA
GATAGCCCCTCCCAATGGGCGGTCCCATATACCATATATGGGGCTTCCTAATACCGC
CCATAGCCACTCCCCCATTGACGTCAATGGTCTCTATATATGGTCTTTCCTATTGACG
TCATATGGGCGGTCCTATTGACGTATATGGCGCCTCCCCCATTGACGTCAATTACGG
TAAATGGCCCGCCTGGCTCAATGCCCATTGACGTCAATAGGACCACCCACCATTGAC
GTCAATGGGATGGCTCATTGCCCATTCATATCCGTTCTCACGCCCCCTATTGACGTCA
ATGACGGTAAATGGCCCACTTGGCAGTACATCAATATCTATTAATAGTAACTTGGCA
AGTACATTACTATTGGAAGGACGCCAGGGTACATTGGCAGTACTCCCATTGACGTCA
ATGGCGGTAAATGGCCCGCGATGGCTGCCAAGTACATCCCCATTGACGTCAATGGG
GAGGGGCAATGACGCAAATGGGCGTTCCATTGACGTAAATGGGCGGTAGGCGTGCC
TAATGGGAGGTCTATATAAGCAATGCTCGTTTAGGGAAC

pCS2-α3_M4Δ8:

CGCCATTCTGCCTGGGGACGTCGGAGCAAGCTTGATTTAGGTGACACTATAGAATAC
AAGCTACTTGTTCTTTTTGCAGGATCGGTACCGGGCCCGCCGCCACCATGGCACATG
TAAGACACTTCCGTACTCTAGTGTCTGGGTTCTATTTCTGGGAGGCCGCTCTATTGCT
CAGCCTAGTCGCCACAAAGGAAACAGACAGCGCCCGCTCCAGATCCGCCCCGATGA
GTCCCAGTGATTTCTTAGATAAATTGATGGGCAGAACCTCTGGGTATGACGCGCGCA
TTCGCCCGAACTTTAAGGGACCCCCAGTAAACGTTACTTGTAATATTTTTATCAATA
GCTTTGGTTCAATTGCAGAAACCACTATGGATTACCGGGTGAATATTTTCTTGCGGC
AAAAGTGGAACGACCCTAGACTCGCGTATTCGGAATACCCCGATGACTCCCTGGATT
TAGATCCTTCTATGCTTGATTCAATTTGGAAGCCCGATTTATTTTTCGCGAACGAGAA
AGGTGCTAATTTTCATGAAGTTACAACCGACAACAAACTACTTCGAATCTTTAAAAA
CGGAAATGTTTTATACTCGATACGGCTTACATTAACTTTGAGCTGCCCCATGGATCT
AAAAAATTTCCCAATGGACGTTCAGACGTGTATTATGCAGCTTGAGTCTTTCGGCTA
CACTATGAACGATCTTATATTCGAGTGGCAGGATGAAGCTCCAGTACAGGTCGCCGA
GGGCTTGACGCTCCCACAGTTTCTGTTAAAAGAGGAGAAGGATTTGCGTTACTGTAC
CAAGCACTATAACACTGGCAAATTTACCTGCATAGAAGTCCGCTTCCACCTGGAGCG
CCAAATGGGTTATTATTTGATTCAAATGTACATCCCATCGCTACTGATAGTTATATTA
TCATGGGTCTCGTTTTGGATTAATATGGACGCAGCTCCGGCTCGGGTGGCTCTCGGT
ATCACTACGGTGTTAACCATGACGACACAATCTTCAGGATCCAGGGCCTCACTGCCT
AAGGTTAGCTACGTGAAAGCAATAGACATATGGATGGCAGTATGCTTACTTTTTGTG
TTTAGTGCGCTCCTTGAGTACGCCGCTGTGAATTTTGTCAGTCGACAGCACAAAGAG
TTGCTACGATTCCGACGTAAGAGGAAAAACAAGACAGAAGCATTCGCACTCGAAAA
ATTCTATCGTTTCTCCGACATGGACGATGAAGTAAGGGAAAGTAGGTTTAGTTTTAC
GGCATATGGTATGGGGCCTTGCCTCCAGGCGAAAGACGGAATGACGCCGAAGGGGC
CTAATCACCCGGTACAAGTAATGCCTAAGTCGCCCGACGAGATGCGAAAGGTCTTC
ATAGACCGGGCGAAGAAAATTGATACGATCTCAAGAGCTTGTTTCCCATTGGCGTTC
CTAATCTTTAACATATTTTATTGGGTTATCTACAAGATCCTCCGTCATTGAGGTACCT
AGCATCGATGGATCCTCGAGCCTCTAGAACTATAGTGAGTCGTATTACGTAGATCCA
GACATGATAAGATACATTGATGAGTTTGGACAAACCACAACTAGAATGCAGTGAAA
AAAATGCTTTATTTGTGAAATTTGTGATGCTATTGCTTTATTTGTAACCATTATAAGC
TGCAATAAACAAGTTAACAACAACAATTGCATTCATTTTATGTTTCAGGTTCAGGGG
GAGGTGTGGGAGGTTTTTTAATTCGCGGCCGCGGCGCCAATGCATTGGGCCCGGTAC
CCAGCTTTTGTTCCCTTTAGTGAGGGTTAATTGCGCGCTTGGCGTAATCATGGTCATA
GCTGTTTCCTGTGTGAAATTGTTATCCGCTCACAATTCCACACAACATACGAGCCGG
AAGCATAAAGTGTAAAGCCTGGGGTGCCTAATGAGTGAGCTAACTCACATTAATTG
CGTTGCGCTCACTGCCCGCTTTCCAGTCGGGAAACCTGTCGTGCCAGCTGCATTAAT
GAATCGGCCAACGCGCGGGGAGAGGCGGTTTGCGTATTGGGCGCTCTTCCGCTTCCT
CGCTCACTGACTCGCTGCGCTCGGTCGTTCGGCTGCGGCGAGCGGTATCAGCTCACT
CAAAGGCGGTAATACGGTTATCCACAGAATCAGGGGATAACGCAGGAAAGAACATG
TGAGCAAAAGGCCAGCAAAAGGCCAGGAACCGTAAAAAGGCCGCGTTGCTGGCGTT
TTTCCATAGGCTCCGCCCCCCTGACGAGCATCACAAAAATCGACGCTCAAGTCAGAG

GTGGCGAAACCCGACAGGACTATAAAGATACCAGGCGTTTCCCCCTGGAAGCTCCC
TCGTGCGCTCTCCTGTTCCGACCCTGCCGCTTACCGGATACCTGTCCGCCTTTCTCCC
TTCGGGAAGCGTGGCGCTTTCTCATAGCTCACGCTGTAGGTATCTCAGTTCGGTGTA
GGTCGTTCGCTCCAAGCTGGGCTGTGTGCACGAACCCCCCGTTCAGCCCGACCGCTG
CGCCTTATCCGGTAACTATCGTCTTGAGTCCAACCCGGTAAGACACGACTTATCGCC
ACTGGCAGCAGCCACTGGTAACAGGATTAGCAGAGCGAGGTATGTAGGCGGTGCTA
CAGAGTTCTTGAAGTGGTGGCCTAACTACGGCTACACTAGAAGGACAGTATTTGGTA
TCTGCGCTCTGCTGAAGCCAGTTACCTTCGGAAAAAGAGTTGGTAGCTCTTGATCCG
GCAAACAAACCACCGCTGGTAGCGGTGGTTTTTTTGTTTGCAAGCAGCAGATTACGC
GCAGAAAAAAAGGATCTCAAGAAGATCCTTTGATCTTTTCTACGGGGTCTGACGCTC
AGTGGAACGAAAACTCACGTTAAGGGATTTTGGTCATGAGATTATCAAAAAGGATC
TTCACCTAGATCCTTTTAAATTAAAAATGAAGTTTTAAATCAATCTAAAGTATATAT
GAGTAAACTTGGTCTGACAGTTACCAATGCTTAATCAGTGAGGCACCTATCTCAGCG
ATCTGTCTATTTCGTTCATCCATAGTTGCCTGACTCCCCGTCGTGTAGATAACTACGA
TACGGGAGGGCTTACCATCTGGCCCCAGTGCTGCAATGATACCGCGAGACCCACGC
TCACCGGCTCCAGATTTATCAGCAATAAACCAGCCAGCCGGAAGGGCCGAGCGCAG
AAGTGGTCCTGCAACTTTATCCGCCTCCATCCAGTCTATTAATTGTTGCCGGGAAGCT
AGAGTAAGTAGTTCGCCAGTTAATAGTTTGCGCAACGTTGTTGCCATTGCTACAGGC
ATCGTGGTGTCACGCTCGTCGTTTGGTATGGCTTCATTCAGCTCCGGTTCCCAACGAT
CAAGGCGAGTTACATGATCCCCCATGTTGTGCAAAAAAGCGGTTAGCTCCTTCGGTC
CTCCGATCGTTGTCAGAAGTAAGTTGGCCGCAGTGTTATCACTCATGGTTATGGCAG
CACTGCATAATTCTCTTACTGTCATGCCATCCGTAAGATGCTTTTCTGTGACTGGTGA
GTACTCAACCAAGTCATTCTGAGAATAGTGTATGCGGCGACCGAGTTGCTCTTGCCC
GGCGTCAATACGGGATAATACCGCGCCACATAGCAGAACTTTAAAAGTGCTCATCA
TTGGAAAACGTTCTTCGGGGCGAAAACTCTCAAGGATCTTACCGCTGTTGAGATCCA
GTTCGATGTAACCCACTCGTGCACCCAACTGATCTTCAGCATCTTTTACTTTCACCAG
CGTTTCTGGGTGAGCAAAAACAGGAAGGCAAAATGCCGCAAAAAAGGGAATAAGG
GCGACACGGAAATGTTGAATACTCATACTCTTCCTTTTTCAATATTATTGAAGCATTT
ATCAGGGTTATTGTCTCATGAGCGGATACATATTTGAATGTATTTAGAAAAATAAAC
AAATAGGGGTTCCGCGCACATTTCCCCGAAAAGTGCCACCTGACGCGCCCTGTAGC
GGCGCATTAAGCGCGGCGGGTGTGGTGGTTACGCGCAGCGTGACCGCTACACTTGC
CAGCGCCCTAGCGCCCGCTCCTTTCGCTTTCTTCCCTTCCTTTCTCGCCACGTTCGCC
GGCTTTCCCCGTCAAGCTCTAAATCGGGGGCTCCCTTTAGGGTTCCGATTTAGTGCTT
TACGGCACCTCGACCCCAAAAAACTTGATTAGGGTGATGGTTCACGTAGTGGGCCAT
CGCCCTGATAGACGGTTTTTCGCCCTTTGACGTTGGAGTCCACGTTCTTTAATAGTGG
ACTCTTGTTCCAAACTGGAACAACACTCAACCCTATCTCGGTCTATTCTTTTGATTTA
TAAGGGATTTTGCCGATTTCGGCCTATTGGTTAAAAAATGAGCTGATTTAACAAAAA
TTTAACGCGAATTTTAACAAAATATTAACGCTTACAATTTCCATTCGCCATTCAGGCT
GCGCAACTGTTGGGAAGGGCGATCGGTGCGGGCCTCTTCGCTATTACGCCAGTCGAC
CATAGCCAATTCAATATGGCGTATATGGACTCATGCCAATTCAATATGGTGGATCTG

GACCTGTGCCAATTCAATATGGCGTATATGGACTCGTGCCAATTCAATATGGTGGAT
CTGGACCCCAGCCAATTCAATATGGCGGACTTGGCACCATGCCAATTCAATATGGCG
GACTTGGCACTGTGCCAACTGGGGAGGGGTCTACTTGGCACGGTGCCAAGTTTGAG
GAGGGGTCTTGGCCCTGTGCCAAGTCCGCCATATTGAATTGGCATGGTGCCAATAAT
GGCGGCCATATTGGCTATATGCCAGGATCAATATATAGGCAATATCCAATATGGCCC
TATGCCAATATGGCTATTGGCCAGGTTCAATACTATGTATTGGCCCTATGCCATATA
GTATTCCATATATGGGTTTTCCTATTGACGTAGATAGCCCCTCCCAATGGGCGGTCCC
ATATACCATATATGGGGCTTCCTAATACCGCCCATAGCCACTCCCCCATTGACGTCA
ATGGTCTCTATATATGGTCTTTCCTATTGACGTCATATGGGCGGTCCTATTGACGTAT
ATGGCGCCTCCCCCATTGACGTCAATTACGGTAAATGGCCCGCCTGGCTCAATGCCC
ATTGACGTCAATAGGACCACCCACCATTGACGTCAATGGGATGGCTCATTGCCCATT
CATATCCGTTCTCACGCCCCCTATTGACGTCAATGACGGTAAATGGCCCACTTGGCA
GTACATCAATATCTATTAATAGTAACTTGGCAAGTACATTACTATTGGAAGGACGCC
AGGGTACATTGGCAGTACTCCCATTGACGTCAATGGCGGTAAATGGCCCGCGATGG
CTGCCAAGTACATCCCCATTGACGTCAATGGGGAGGGGCAATGACGCAAATGGGCG
TTCCATTGACGTAAATGGGCGGTAGGCGTGCCTAATGGGAGGTCTATATAAGCAATG
CTCGTTTAGGGAAC

Data S2. Codon optimized human GlyRα3 sequence used for protein production.

ATGGCACATGTAAGACACTTCCGTACTCTAGTGTCTGGGTTCTATTTCTGGGAGGCC
GCTCTATTGCTCAGCCTAGTCGCCACAAAGGAAACAGACAGCGCCCGCTCCAGATC
CGCCCCGATGAGTCCCAGTGATTTCTTAGATAAATTGATGGGCAGAACCTCTGGGTA
TGACGCGCGCATTCGCCCGAACTTTAAGGGACCCCCAGTAAACGTTACTTGTAATAT
TTTTATCAATAGCTTTGGTTCAATTGCAGAAACCACTATGGATTACCGGGTGAATAT
TTTCTTGCGGCAAAAGTGGAACGACCCTAGACTCGCGTATTCGGAATACCCCGATGA
CTCCCTGGATTTAGATCCTTCTATGCTTGATTCAATTTGGAAGCCCGATTTATTTTTC
GCGAACGAGAAAGGTGCTAATTTTCATGAAGTTACAACCGACAACAAACTACTTCG
AATCTTTAAAAACGGAAATGTTTTATACTCGATACGGCTTACATTAACTTTGAGCTG
CCCCATGGATCTAAAAAATTTCCCAATGGACGTTCAGACGTGTATTATGCAGCTTGA
GTCTTTCGGCTACACTATGAACGATCTTATATTCGAGTGGCAGGATGAAGCTCCAGT
ACAGGTCGCCGAGGGCTTGACGCTCCCACAGTTTCTGTTAAAAGAGGAGAAGGATT
TGCGTTACTGTACCAAGCACTATAACACTGGCAAATTTACCTGCATAGAAGTCCGCT
TCCACCTGGAGCGCCAAATGGGTTATTATTTGATTCAAATGTACATCCCATCGCTAC
TGATAGTTATATTATCATGGGTCTCGTTTTGGATTAATATGGACGCAGCTCCGGCTCG
GGTGGCTCTCGGTATCACTACGGTGTTAACCATGACGACACAATCTTCAGGATCCAG
GGCCTCACTGCCTAAGGTTAGCTACGTGAAAGCAATAGACATATGGATGGCAGTAT
GCTTACTTTTTGTGTTTAGTGCGCTCCTTGAGTACGCCGCTGTGAATTTTGTCAGTCG
ACAGCACAAAGAGTTGCTACGATTCCGACGTAAGAGGAAAAACAAGACAGAAGCA
TTCGCACTCGAAAAATTCTATCGTTTCTCCGACATGGACGATGAAGTAAGGAAAGT
AGGTTTAGTTTTACGGCATATGGTATGGGGCCTTGCCTCCAGGCGAAAGACGGAATG

ACGCCGAAGGGGCCTAATCACCCGGTACAAGTAATGCCTAAGTCGCCCGACGAGAT
GCGAAAGGTCTTCATAGACCGGGCGAAGAAAATTGATACGATCTCAAGAGCTTGTT
TCCCATTGGCGTTCCTAATCTTTAACATATTTTATTGGGTTATCTACAAGATCCTCCG
TCATGAAGATATCCATCAACAACAGGACCTGGTCCCGAGGGGCAGCCATCACCACC
ACCATCATCACCATTGA

Table S1. Cryo-EM data collection, refinement, and validation statistics for glycine datasets.

Sample	hGlyRα3-0.1g		hGlyRα3-1g
PDBid	9BVH	9BU3	9BVJ
EMBD ID	44933	44900	44934
**Data Collection and Processing**	Apo	Des	Des
Microscope and Location	Titan Krios (CWRU)		Titan Krios (NYSBC)
Magnification	81,000		81,000
Voltage	300kV		300kV
Data Collection Mode	Super-resolution		Super-resolution
Camera	K3		K3
Physical Pixel Size	1.1 Å/pixel		1.069 Å/pixel
Defocus Range (μM)	-0.75 to -1.5		-0.75 to -1.75
Number of Micrographs Used	7,957		5,050
Dose per Frame	1.24 e-/Å^2		1.3 e-/Å^2
Number of Frames/Movie	50		50
Initial Particle Number	3,914,212		2,874,967
Final Particle Number	99,442	106,791	435,078
Symmetry	C5	C5	C5
Resolution (unmasked, Å)	2.96	3.03	3.04
Resolution (masked, Å)	2.46	2.58	2.80
Map Resolution Range	2.3-3.3	2.5-3.3	2.5-5.5
Map Sharpening B-factor (Å^2)	-20	-20	-20
**Refinement**			
Initial Model Used	hGlyRα3-0.1g-Des	5TIO	hGlyRα3-0.1g-Des
Composition			
Protein Residues	1710	1715	1715
Non Hydrogen Atoms	14680	14630	14620
Glycan (NAG) (molecule)	10	10	10
Glycine (molecule)	0	5	5
Water	20	15	10
PIO	20	25	25
PX4	20	20	20
CLR/Y01	5	0	0
Bonds (RMSD)			
Length (Å) (# > 4σ)	0	0	0
Angles (º) (# > 4σ)	7	0	0
Ramachandran Plot (%)			
Outliers	0	0	0
Allowed	1.48	1.18	0.89
Favored	98.52	98.82	99.11
Rotamer Outliers (%)	0	0	0
Molprobity Score	1.18	1.09	1.34
Molprobity Clashscore	3.96	2.96	6.23

Table S2. Cryo-EM data collection, refinement and validation statistics for Zn^{2+} datasets.

Sample	hGlyRα3-100Zn		hGlyRα3-1Zn
PDBid	9BZP	9BWG	9BWJ
EMBD ID	45073	44974	44976
**Data Collection and Processing**	Apo	Des	Des
Microscope and Location	Titan Krios (CWRU)		Titan Krios (PNCC)
Magnification	105,000		105,000
Voltage	300kV		300kV
Data Collection Mode	Super-resolution		Super-resolution
Camera	K3		K3
Physical Pixel Size	0.84 Å/pixel		0.8266 Å/pixel
Defocus Range (μM)	-0.75 to -1.5		-0.8 to -2.5
Number of Micrographs Used	5,261		6,246
Dose per Frame	1.22 e-/$Å^2$		1.2 e-/$Å^2$
Number of Frames/Movie	50		50
Initial Particle Number	1,447,097		2,008,979
Final Particle Number	154,787	32,894	21,478
Symmetry	C5	C5	C5
Resolution (unmasked, Å)	2.51	2.93	3.33
Resolution (masked, Å)	2.21	2.59	2.88
Map Resolution Range	2.1-5	2.4-6	2.7-7.4
Map Sharpening B-factor ($Å^2$)	-20	-20	-20
**Refinement**			
Initial Model Used	hGlyRα3-0.1g-Apo	hGlyRα3-0.1g-Des	hGlyRα3-100Zn-Des
Composition			
Protein Residues	1730	1735	1735
Non Hydrogen Atoms	14705	14810	14805
Glycan (NAG) (molecule)	10	10	10
Glycine (molecule)	0	5	5
Zinc (molecule)	10	5	5
Water	15	15	10
PIO	0	25	25
PX4	35	20	20
CLR/Y01	0	0	0
Bonds (RMSD)			
Length (Å) (# > 4σ)	0	0	0
Angles (º) (# > 4σ)	0	25	5
Ramachandran Plot (%)			
Outliers	0	0	0
Allowed	2.05	1.99	1.17
Favored	97.95	98.01	98.83
Rotamer Outliers (%)	0	0	0
Molprobity Score	1.26	1.37	1.28
Molprobity Clashscore	4.81	6.7	5.18

Table S3. Cryo-EM data collection, refinement, and validation statistics for acidic datasets.

Sample	hGlyRα3-0.1g-pH6.4			hGlyRα3-1g-pH6.4
PDBid	9BWC	9BWB	9BWE	9BU2
EMBD ID	44964	44963	44964	44899
**Data Collection and Processing**	Closed	Des	Inter	Des
Microscope and Location	Titan Krios (CWRU)			Glacios (CWRU)
Magnification	105000			130000
Voltage	300kV			200kV
Data Collection Mode	Super-resolution			Super-resolution
Camera	K3			Falcon 4
Physical Pixel Size	0.84 Å/pixel			0.91 Å/pixel
Defocus Range (μM)	-0.75 to -2.0			-0.8 to -2.2
Number of Micrographs Used	15,072			2,020
Dose per Frame	1.24 e-/Å^2			0.96 e-/Å^2
Number of Frames/Movie	50			1498 frames / 26 fractions
Initial Particle Number	8,344,616			1,281,017
Final Particle Number	132,119	165,143	283,814	100,328
Symmetry	C5	C5	C5	C5
Resolution (unmasked, Å)	2.45	2.45	3.3	3.33
Resolution (masked, Å)	2.19	2.21	3.07	2.87
Map Resolution Range	2.1-4.2	2.1-4.2	2.8-5.2	2.7-6.2
Map Sharpening B-factor (Å^2)	-20	-20	-20	-20
**Refinement**				
Initial Model Used	hGlyRα3-100Zn-Apo	hGlyRα3-100Zn-Des	hGlyRα3-0.1g-pH6.4-Des	hGlyRα3-0.1g-pH6.4-Des
Composition				
Protein Residues	1735	1735	1735	1735
Non Hydrogen Atoms	14740	14820	14615	14795
Glycan (NAG) (molecule)	10	10	10	10
Glycine (molecule)	5	5	5	5
Water	35	40	0	5
PIO	5	25	30	25
PX4	25	20	5	20
CLR/Y01	0	0	0	0
Bonds (RMSD)				
Length (Å) (# > 4σ)	0	0	0	0
Angles (º) (# > 4σ)	18	15	0	0
Ramachandran Plot (%)				
Outliers	0	0	0	0
Allowed	2.63	2.11	3.22	1.46
Favored	97.37	97.89	96.78	98.54
Rotamer Outliers (%)	0	0	0	0
Molprobity Score	1.53	1.04	1.56	1.13
Molprobity Clashscore	6.27	7.04	6.59	3.4

Table S4 – Simulation Protocol.

Step	Ensemble	Time Step [ps]	Simulation Time [ps]	Backbone restraints [kJ/mol/nm^2]	Side Chain restraints [kJ/mol/nm^2]	Lipid Phosphate restraints [kJ/mol/nm^2]
1	NVT	0.001	125	4000	2000	1000
2	NVT	0.001	125	2000	1000	400
3	NPT	0.001	125	1000	500	400
4	NPT	0.002	500	500	200	200
5	NPT	0.002	50,000	500	0	0
6<br><br>Water density analysis	NPT	0.002	200,000	0 (except for His311 and Lys386)	0	0
7<br><br>ZN distance analysis	NPT	0.002	1,000,000	0 (except for His311 and Lys386)	0	0

Movie S1. Movement mode 0 from 3D variability analysis of the hGlyRa3-0.1g-pH6.4 intermediate state.
